# *tert*-Butyl Nitrite-Induced Radical Nitrile Oxidation Cycloaddition: Synthesis of Isoxazole/Isoxazoline-Fused Benzo 6/7/8-membered Oxacyclic Ketones

**DOI:** 10.3390/molecules29061202

**Published:** 2024-03-07

**Authors:** Jian-Kang Cao, Tian-Zheng Cao, Qian-Wen Yue, Ying Ma, Chuan-Ming Yang, Hong-Xi Zhang, Ya-Chen Li, Qiao-Ke Dong, Yan-Ping Zhu, Yuan-Yuan Sun

**Affiliations:** 1 Key Laboratory of Molecular Pharmacology and Drug Evaluation, Ministry of Education, Collaborative Innovation Center of Advanced Drug Delivery System and Biotech Drugs in Universities of Shandong, School of Pharmacy, Yantai University, Yantai 264005, China; cjk18954343136@163.com (J.-K.C.); tianzheng202201@163.com (T.-Z.C.); 19553508187@163.com (Q.-W.Y.); 15935574380@163.com (Y.M.); 15254990262@163.com (C.-M.Y.); 13355081055@163.com (H.-X.Z.); a2732586174@163.com (Y.-C.L.); 19558912885@163.com (Q.-K.D.); 2 Anhui Laboratory of Molecule-Based Materials, College of Chemistry and Materials Science, Anhui Normal University, Wuhu 241000, China

**Keywords:** *tert*-butyl nitrite (TBN), polycyclic architectures, isoxazole/isoxazoline, aryl methyl ketones, intramolecular cycloaddition

## Abstract

A practical metal-free and additive-free approach for the synthesis of 6/7/8-membered oxacyclic ketone-fused isoxazoles/isoxazolines tetracyclic or tricyclic structures is reported through C_sp_^3^–H bond radical nitrile oxidation and the intramolecular cycloaddition of alkenyl/alkynyl-substituted aryl methyl ketones. This convenient approach enables the simultaneous formation of isoxazole/isoxazoline and 6/7/8-membered oxacyclic ketones to form polycyclic architectures by using *tert*-butyl nitrite (TBN) as a non-metallic radical initiator and N–O fragment donor.

## 1. Introduction

**Figure 1:**
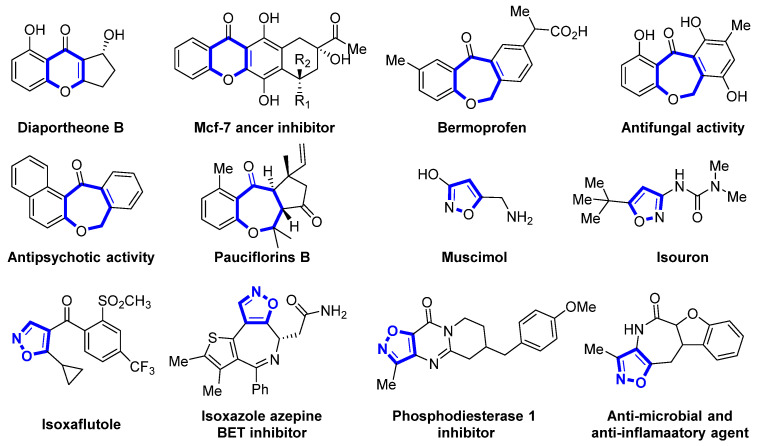
Some drugs with pharmacological activity containing benzo[*b*]oxygenes or isoxazole frameworks.

Polycyclic structures containing heteroatoms are regarded as important structural motifs in the realm of organic chemistry and pharmaceuticals. They are present in various natural products, agrochemicals, and physiologically active molecules and play a significant role in drug synthesis and discovery [1,2]. The benzo oxacyclic ketone skeleton is an important scaffold for multiring structures, such as benzochromones and their derivatives. These structures are found in numerous natural products and pharmaceuticals, playing a pivotal role in the formation of polycyclic systems (Figure 1) [3,4,5,6,7,8].

Isoxazole/isoxazoline, a five-membered heterocyclic ring, is present in numerous biologically significant compounds known for their anti-inflammatory, antifungal, anticancer, and antimicrobial properties. Its ability to interact with the target protein through multiple non-covalent bonds makes it a pivotal drug component in various pharmaceutical formulations [9,10,11,12].

Due to the significant biological activities associated with the benzo oxacyclic ketone and isoxazole/isoxazoline skeletons, the development of efficient methods to merge these two entities is highly significant and desirable in the realms of medicinal and synthetic chemistry. Fusing two or more heterocycles to form a tricyclic or tetracyclic fused heterocycle is of interest to access polycyclic architectures. These polycyclic architectures demonstrate enhanced biological activity [13,14].

Numerous methods have been reported for synthesizing small ring (3–6 membered) and large ring (≥12 membered) compounds, including the Diels Alder reaction, Corey Nicolaou macrocycle esterification reaction, Keck macrocycle esterification reaction, and olefin metathesis reaction. Advancements in transition-metal-catalyzed closed-loop metathesis, olefin reactions, small ring cycloaddition, and hydrogenation acylation have led to progress in synthesizing medium-sized ring (7–11 membered) compounds. The intermolecular cycloaddition reaction is also effective for the formation of medium-sized rings [15]. However, predicting the reactivity of these compounds is challenging due to unfavorable cross-ring tension and entropy effects, making their synthesis both difficult and intriguing. Medium-sized rings, particularly seven- and eight-membered ones, pose significant challenges in synthesis [16,17,18,19].

**Scheme 1:**
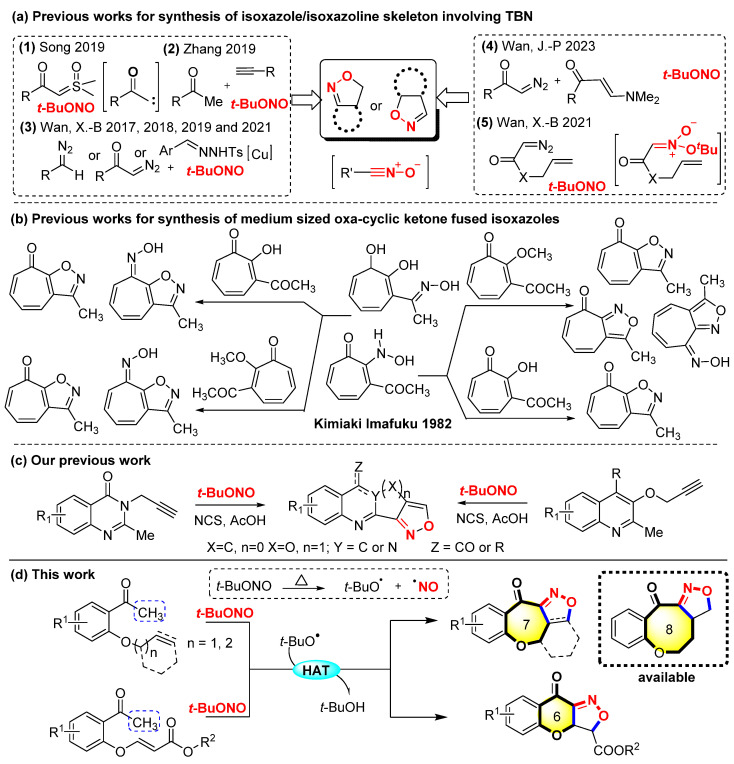
Strategy for the synthesis of skeleton-fused isoxazole/isoxazoline. (HAT: Hydrogen Atom Transfer) [27,28,29,30,31,32,33,34].

Reactions employing *tert*-butyl nitrite (TBN) as both a free radical initiator and N–O fragment donor have emerged as an important tool for isoxazole/isoxazoline synthesis over the past few years [20,21,22,23,24,25,26]. Song et al. developed a new [2 + 1 + 1 + 1] annulation reaction of sulfoxonium ylides with TBN for the first time to synthesize furoxans and isoxazoles [27]. Zhang et al. reported the graceful synthesis of isoxazoles from methyl ketones, terminal alkynes, and TBN under catalyst-free conditions [28]. Wan, X.-B. et al. reported the graceful cycloaddition reactions for the synthesis of isoxazoles from diazo compounds or *N*-tosylhydrazones with alkenes or *β*-keto esters activated by *tert*-butyl nitrite [29,30,31]. These approaches are robust and can deliver fully substituted isoxazoles. In a recent study, Wan, J.-P. et al. reported a refined metal-catalyzed strategy for the synthesis of isomeric isoxazoles through the reactions of enaminones, diazo compounds, and TBN under different Cu- and Ag-catalyzed conditions [32]. The synthesis of isoxazoline-fused bicyclic compounds poses challenges, particularly under transition-metal-free conditions. Instead, Wan, X.-B. et al. used intramolecular acyclic nitronate olefin cycloaddition reactions via the in situ generated acyclic nitronates combined with cascade [3 + 2] cycloaddition and *tert*-butyloxy group elimination to enable the formation of diverse γ-lactone-fused isoxazolines and even tricyclic isoxazolines (Scheme 1a) [33]. A metal-free method had already been used to synthesize 3-methyl-1,8-dihydrocycloheptapyrazol-8-one derivatives and isoxazole-fused seven-membered oxacyclic ketones by Imafuku in 1982 (Scheme 1b) [34].

Recently, our group successfully demonstrated an efficient synthetic method to synthesize diverse isoxazole-fused tricyclic quinazoline alkaloids and their derivatives (Scheme 1c) [35]. We gained inspiration from the synthesis of the 3-acyl-isoxazoles and Δ^2^-isoxazolines series compounds reported by Zhang et al. [34] based on their previous research. Drawing inspiration from these investigations, a metal-free and additive-free method for C_sp_^3^–H bond radical nitrile oxidation and the intramolecular cycloaddition of alkenyl/alkynyl-substituted aryl methyl ketones to synthesize 6/7/8-membered oxacyclic ketone-fused isoxazoles/isoxazolines tetracyclic or tricyclic structures is reported. This convenient approach enables the simultaneous formation of the isoxazole/isoxazoline and 6/7/8-membered oxacyclic ketone, thereby leading to the formation of the polycyclic architectures using TBN as a non-metallic radical initiator and N−O fragment donor. (Scheme 1d).

## 2. Results and Discussion

**Figure 2:**
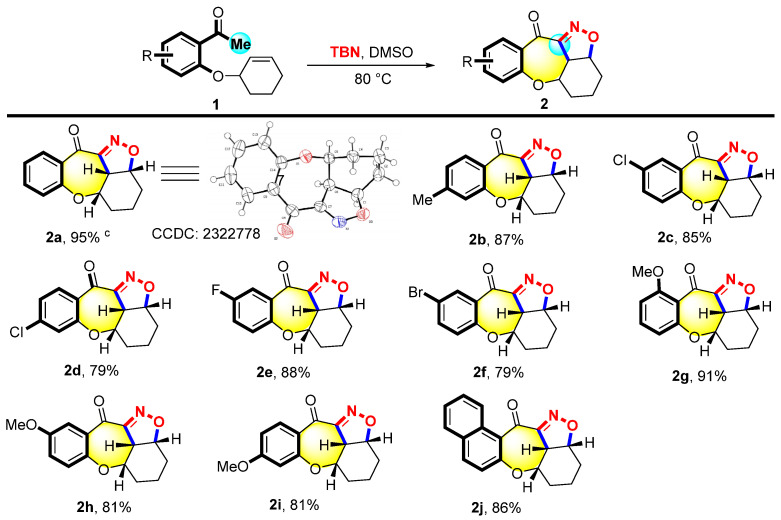
Scope of 3-(2-acetophenoxy) cyclohexene ^a,b^. ^a^ Reaction condition: **1** (0.1 mmol) and TBN (0.4 mmol) were heated in DMSO (2 mL) at 80 °C for 10 h. ^b^ Isolated yields. ^c^ The molecular structure of **2a** with ellipsoids at the 50% probability level.

Firstly, the reaction conditions were optimized, and the results are summarized in Tables S2–S4 (supporting information). The substrate scope of **2** was investigated under optimized conditions. As shown in Figure 2, the method displayed excellent tolerance for structure **1**, substituted with electron-donating groups, and can yield the desired products **2b**, **2g–2i**. A series of substrates with a methyl group at the C4 (**1b**) and the methoxyl group at the C4 (**1i**), C5 (**1h**), and C6 (**1g**) positions led to the corresponding products with yields ranging from 81% to 91%. On the other hand, structure **1** substituted with electron-withdrawing groups such as Cl, Br, and F at the C4 or C5 position performed the reaction smoothly to give the desired products **2c–2f** in good yields (79–88%). The naphthalenyl-substituted substrate **1j** was also suitable for this reaction to deliver the desired product **2j** with an 86% yield. X-ray single crystal diffraction was employed to determine the crystal structure of product **2a**.

**Figure 3:**
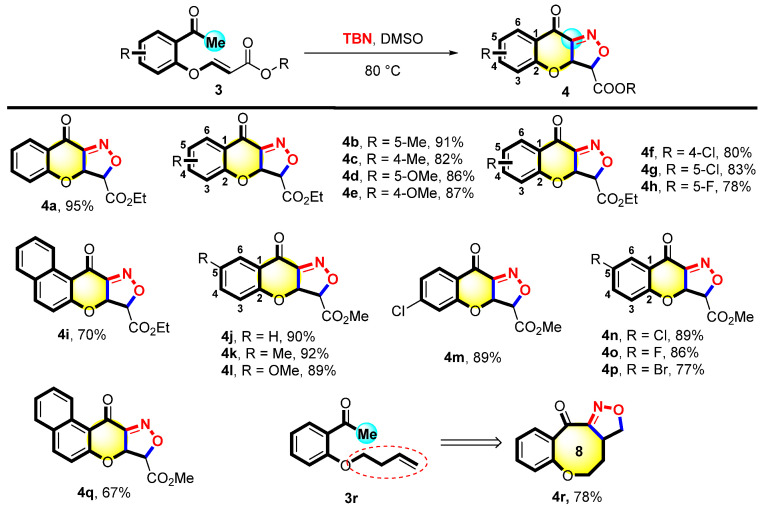
Scope of 3-(2-acetylphenoxy)acrylates ^a,b^. ^a^ Reaction condition: **3** (0.1 mmol) and TBN (0.7 mmol) were heated in DMSO (2 mL) at 80 °C for 10 h. ^b^ Isolated yields.

Moreover, the reaction between various acrylates **3** with TBN was explored. It is evident from Figure 3 that **3a** was successfully converted into the expected product **4a** with a 95% yield. Surprisingly, different acetophenones **3b–3e** with electron-donating substituents (such as 4-Me, 5-Me, 4-OMe, and 5-OMe) reacted analogously, yielding the corresponding products **4b–4e** with 82–91% yields. Halogen–halogen atom substrates formed the corresponding products (**4f–4h**) with 78–83% yields. Furthermore, the side chain ethyl ester was converted to methyl ester and proceeded under standard conditions, yielding the desired products (**4j–4q**) within 67–92% yields. When the benzene ring of the template substrate **3** became a naphthalene ring, **3i** and **3q** yielded the corresponding products **4i** and **4q** in 70% and 67% yields, respectively. We synthesized the raw material *O*-acetylphenoxybutene (**3r**). Subsequently, **3r** performed the reaction under the optimal conditions to give a polycyclic compound containing an eight-membered ring (**4r**) with a 78% yield.

**Figure 4:**
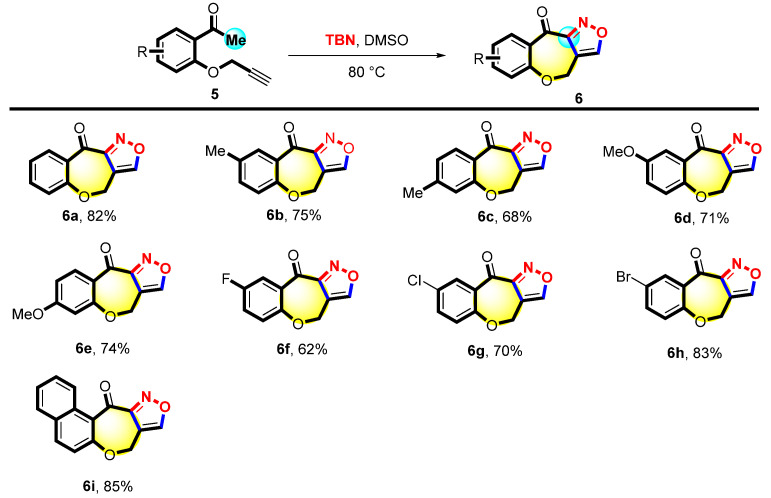
Scope of 3-(2-acetophenoxy) propyne ^a,b^. ^a^ Reaction condition: **5** (0.1 mmol) and TBN (0.5 mmol) were heated in DMSO (2 mL) at 80 °C for 10 h. ^b^ Isolated yields.

Next, substrate **5** was explored to obtain a series of derivatives with an isoxazole structure, and the reaction conditions were further optimized for the synthesis (Table S4). The scope of **6** was studied under the optimal conditions. As shown in Figure 4, substrate **5a** was smoothly transformed into the corresponding product with a yield of 82%. A series of **5** with different substitutions (4-Me, 5-Me 4-OMe, and 4-OMe) was investigated. The desired products **6b–6e** were obtained with 68%-75% yields. The 1-(2-(prop-2-yn-1-yloxy)phenyl)ethan-1-ones (**5f–5h**) attached with halogen atoms (e.g., 5-F, 5-Cl, and 5-Br) were also tolerated in the reaction, yielding the corresponding products **6f–6h** with 62%-83% yields. Substrate **5i** was also found to be suitable for this reaction, giving the desired product **6i** with an 85% yield.

**Scheme 2:**
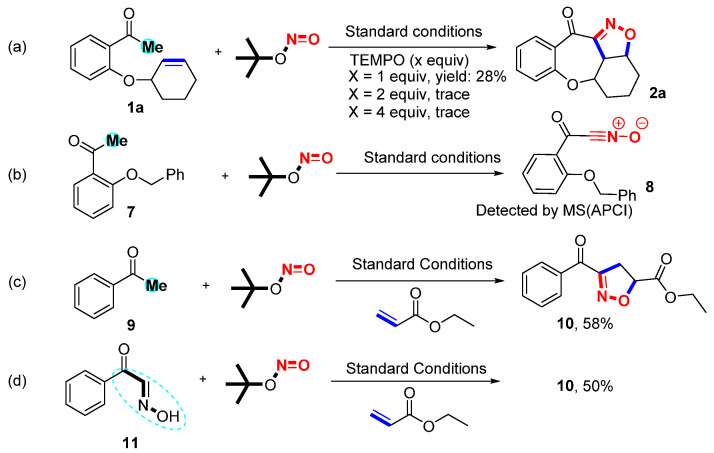
Control experiments. (**a**) Radical capture. (**b**) Formation of intermediate **8**. (**c**) Formation of 4,5-dihydroisoxazole **8b** via intermolecular cycloaddition under standard conditions. (**d**) Formation of **10** from intermediate **11** under standard conditions.

Several control experiments were carried out to investigate the reaction mechanism (Scheme 2) [36,37,38]. The reaction was restrained completely and trace amounts of **2a** were observed when a 2.0-equivalent radical scavenger 2,2,6,6-tetramethyl-1-piperidinyl (TEMPO) was added to the standard reaction. This result revealed that the reaction proceeded through a radical pathway. Next, **1a** and TBN were reacted under standard conditions for 20 min to identify the possible intermediates. However, only **2a** was detected by MS (APCI) because the intermediate nitrile oxide **E** shares the same relative molecular mass as **2a**. The result of MS is ambiguous because the masses **2a** and **E** are the same. Alternative approaches were performed to confirm this by subjecting substrates **7** to standard conditions for 20 min to detect **8** nitrile oxides via MS (APCI). A group of intermolecular reactions was used to further explore the reaction mechanism by using **9**, **11**, and ethyl acrylate. Under the optimal conditions, the desired product **10** was produced with yields of 58% and 50% from **9** and **11**. These results disclosed that nitrile oxide was the potential intermediate for this protocol.

**Scheme 3:**
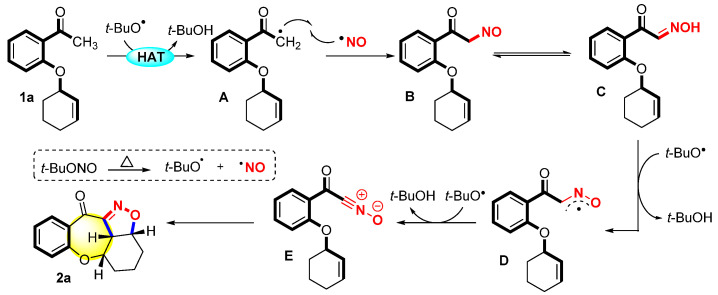
Proposed mechanism.

Based on the evidence presented above and the related literature [28,35,36,39,40,41], a plausible reaction pathway was proposed (Scheme 3). First, TBN was transformed into NO and *^t^*BuO radicals through thermal homolysis. The substrate **1a** underwent hydrogen abstraction with the *^t^*BuO radical to afford intermediate **A**. Then, intermediate **A** and **the** NO radical performed radical cross-coupling to produce intermediate **B**. Intermediate **B** underwent tautomerization to generate oxime **C**, which was further conducted two times via hydrogen abstraction with the *^t^*BuO radical to generate the nitrile oxide intermediate **E**. Finally, the nitrile oxide intermediate **E** underwent 1,3-dipolar cycloaddition with an intramolecular alkene to produce the final product **2a**.

## 3. Materials and Methods

### 3.1. General Information

Analytical thin layer chromatography (TLC) was performed by using pre-coated silica gel HF254 glass plates. Column chromatography was performed by using silica gel (200–300 mesh). The ^1^H NMR and ^13^C NMR spectra were recorded on a Bruker Advance 500 MHz instrument at 500 MHz (^1^H NMR) and 126 MHz (^13^C NMR). We used the residual solvent peak in CDCl_3_ as an internal reference (*δ* = 7.26 for ^1^H and *δ* = 77.0 for ^13^C{^1^H}). Chemical shifts (*δ*) are reported in ppm relative to the internal standard of tetramethylsilane (TMS). The coupling constants (*J*) are quoted in Hz (hertz). Resonances are described as s (singlet), d (doublet), t (triplet), q (quartet), m (multiplet), br (broad), or combinations thereof. High-resolution mass spectra (HRMS) were obtained on Thermo Scientific Q-Exactive (ESI mode, Q-Exactive Orbitrap MS system). The melting points were measured with the SGW X-4 apparatus. Data collection for the crystal structure was performed by using Mo Kα radiation on a Bruker Smart APEX CCD area-detector diffractometer.

### 3.2. Synthetic Procedures

**Scheme 4:**
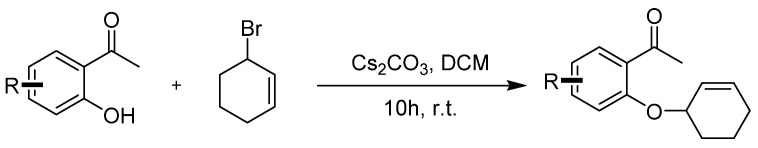
General procedure for synthesis of 1-(2-(cyclohex-2-en-1-yloxy)phenyl)ethan-1-one **1a–1j**.

Compounds **1a–1j** were prepared according to the referenced literature [42]. To a solution of 1-(2-hydroxyphenyl)ethan-1-one) (1.0 equiv.) and Cs_2_CO_3_ (3.0 equiv.) in CH_2_Cl_2_ (0.1 M), a solution of 3-bromocyclohex-1-ene (2.0 equiv.) in CH_2_Cl_2_ (0.5 M) was added dropwise at room temperature and stirred for 10 h. After the reaction was completed, 50 mL of water was added to the mixture and then extracted with DCM 3 times (3 × 50 mL). The extract was dried over anhydrous Na_2_SO_4_ and concentrated under reduced pressure. The crude residues were purified by column chromatography using an ethyl acetate/petroleum ether mixture to obtain the desired products (Scheme 4).

**Scheme 5:**
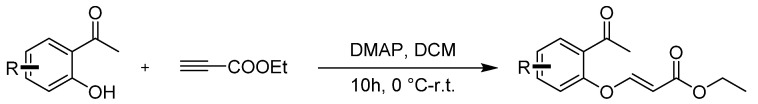
General procedure for synthesis of ethyl (E)-3-(2-acetylphenoxy)acrylate **3a–3q**.

Compounds **3a–3q** were prepared according to the referenced literature [39,40]. To a solution of 1-(2-hydroxyphenyl)ethan-1-one) (1.0 equiv.) and DMAP (0.1 equiv.) in CH_2_Cl_2_ (0.1 M), a solution of ethyl acetylenecarboxylate (2.0 equiv.) was added dropwise at room temperature and stirred for 10 h. After the reaction was completed, 50 mL of water was added to the mixture and then extracted with DCM 3 times (3 × 50 mL). The extract was dried over anhydrous Na_2_SO_4_ and concentrated under reduced pressure. The crude residues were purified by column chromatography using an ethyl acetate/petroleum ether mixture to obtain the desired products (Scheme 5).

**Scheme 6:**
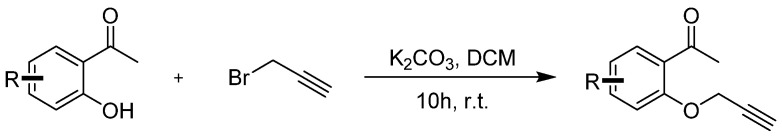
General procedure for synthesis of 1-(2-(prop-2-yn-1-yloxy)phenyl)ethan-1-one **5g–5i**.

Compounds **5a–5i** were prepared according to the referenced literature [43,44,45]. To a solution of 1-(2-hydroxyphenyl)ethan-1-one) (1.0 equiv.) and K_2_CO_3_ (3.0 equiv.) in CH_2_Cl_2_ (0.1 M), a solution of 3-bromoprop-1-yne (2.0 equiv.) in CH_2_Cl_2_ (0.5 M) was added dropwise at room temperature and stirred for 10 h. After the reaction was completed, 50 mL of water was added to the mixture and then extracted with DCM 3 times (3 × 50 mL). The extract was dried over anhydrous Na_2_SO_4_ and concentrated under reduced pressure. The crude residues were purified by column chromatography using an ethyl acetate/petroleum ether mixture to obtain the desired products (Scheme 6).

**Scheme 7:**
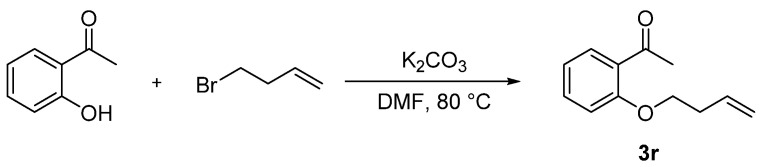
General procedure for synthesis of 1-(2-(but-3-en-1-yloxy)phenyl)ethan-1-one (**3r**).

Compound **3r** was prepared according to the referenced literature [46]. To a solution of 1-(2-(but-3-en-1-yloxy)phenyl)ethan-1-one (1.0 equiv.) and K_2_CO_3_ (1.0 equiv.), a solution of 4-bromo-1-butene (1.2 equiv.) in DMF (4 mL) was added dropwise at 80 °C and stirred for 24 h. After the reaction was completed, 50 mL of water was added to the mixture and then extracted with DCM 3 times (3 × 50 mL). The extract was dried over anhydrous Na_2_SO_4_ and concentrated under reduced pressure. The crude residues were purified by column chromatography using an ethyl acetate/petroleum ether mixture to obtain the desired products (Scheme 7).

**Scheme 8:**
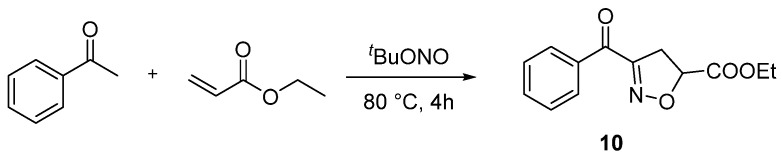
General procedure for synthesis of ethyl 3-benzoyl-4,5-dihydroisoxazole-5-carboxylate (**10**).

Compound **10** was prepared according to the referenced literature [28]. A mixture of acetophenone (1 equiv.), ethyl acrylate (3 equiv.), and *^t^*BuONO (3 equiv.) was dissolved in DMSO (2.0 mL). Then, the mixture was reacted under 80 °C for 4 h. After the reaction was completed, 50 mL of water was added to the mixture and then extracted with EtOAc 3 times (3 × 50 mL). The extract was dried over anhydrous Na_2_SO_4_ and concentrated under reduced pressure. The crude residues were purified by column chromatography by using an ethyl acetate/petroleum ether mixture to obtain the desired product (Scheme 8).

**Scheme 9:**
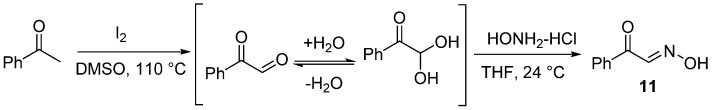
General procedure for synthesis of (*E*)-2-oxo-2-phenylacetaldehyde oxime (**11**).

Compound **11** was prepared according to the referenced literature [36,41]. A mixture of acetophenone (1.0 equiv.) and I_2_ (1.6 equiv.) was reacted under 110 °C for 10 h. Phenyl glyoxal was afforded without further purification. Then, hydroxylamine hydrochloride (1.0 equiv.) was added to a solution of phenyl glyoxal in THF (40 mL), and the reaction mixture was reacted under 24 °C for 12 h. After the reaction was completed, 50 mL of water was added to the mixture and then extracted with EtOAc 3 times (3 × 50 mL). The extract was dried over anhydrous Na_2_SO_4_ and concentrated under reduced pressure. The crude residues were purified by column chromatography using an ethyl acetate/petroleum ether mixture to obtain the desired product (Scheme 9).

### 3.3. Characterization of Products

*2a,2a^1^,3,4,5,5a-Hexahydro-11H-2,6-dioxa-1-azadibenzo[cd,g]azulen-11-one* (**2a**), 38 mg, 95%, white solid, m.p.: 118–119 °C. ^1^H NMR (500 MHz, CDCl_3_) *δ* (ppm) 7.99 (dd, *J* = 7.9, 1.8 Hz, 1H), 7.57 (td, *J* = 7.7, 1.8 Hz, 1H), 7.31–7.21 (m, 1H), 7.12 (dd, *J* = 8.1, 1.1 Hz, 1H), 4.87 (dt, *J* = 10.4, 4.4 Hz, 1H), 4.31 (td, *J* = 6.7, 4.1 Hz, 1H), 3.70 (dd, *J* = 10.4, 7.2 Hz, 1H), 2.26–2.00 (m, 2H), 1.96–1.80 (m, 2H), 1.79–1.54 (m, 2H). ^13^C NMR (126 MHz, CDCl_3_) *δ* (ppm) 184.9, 160.7, 158.4, 136.1, 129.9, 129.8, 124.9, 123.3, 81.3, 78.0, 49.2, 27.8, 23.7, 14.8. HRMS (ESI): *m*/*z* [M + H]^+^ calcd for C_14_H_14_NO_3_: 244.0968; found: 244.0966.
*8-Methyl-2a,2a^1^,3,4,5,5a-hexahydro-11H-2,6-dioxa-1-azadibenzo[cd,g]azulen-11-one* (**2b**), (silica gel: 200–300 mesh, solvent system: petroleum ether/ethyl acetate = 10:1–5:1), 31 mg, 87%, light yellow solid, m.p.: 135–136 °C. ^1^H NMR (500 MHz, CDCl_3_) *δ* (ppm) 7.90 (d, *J* = 8.0 Hz, 1H), 7.05 (dd, *J* = 8.0, 1.6 Hz, 1H), 6.92 (d, *J* = 1.6 Hz, 1H), 4.83 (dt, *J* = 10.3, 4.4 Hz, 1H), 4.27 (ddd, *J* = 7.1, 5.8, 4.0 Hz, 1H), 3.69 (dd, *J* = 10.4, 7.1 Hz, 1H), 2.38 (s, 3H), 2.14 (dddd, *J* = 37.4, 13.8, 10.9, 5.7 Hz, 2H), 1.95–1.75 (m, 3H), 1.74–1.53 (m, 2H). ^13^C NMR (126 MHz, CDCl_3_) *δ* (ppm) 184.5, 161.1, 158.5, 147.9, 129.8, 126.9, 125.9, 123.6, 80.9, 77.8, 49.6, 27.9, 23.9, 21.7, 14.7. HRMS (ESI): *m*/*z* [M + H]^+^ calcd for C_15_H_16_NO_3_: 258.1125; found: 258.1123.
*9-Chloro-2a,2a^1^,3,4,5,5a-hexahydro-11H-2,6-dioxa-1-azadibenzo[cd,g]azulen-11-one* (**2c**), (silica gel: 200–300 mesh, solvent system: petroleum ether/ethyl acetate = 10:1–5:1), 24 mg, 85%, light brown solid, m.p.: 135–136 °C. ^1^H NMR (500 MHz, CDCl_3_) *δ* (ppm) 7.89 (d, *J* = 2.7 Hz, 1H), 7.48 (dd, *J* = 8.6, 2.6 Hz, 1H), 7.06 (d, *J* = 8.6 Hz, 1H), 4.86 (dt, *J* = 10.3, 4.4 Hz, 1H), 4.29 (td, *J* = 6.7, 4.1 Hz, 1H), 3.70 (dd, *J* = 10.4, 7.2 Hz, 1H), 2.23–1.97 (m, 2H), 1.84 (ddq, *J* = 23.1, 9.2, 4.5, 3.9 Hz, 2H), 1.77–1.54 (m, 2H). ^13^C NMR (126 MHz, CDCl_3_) *δ* (ppm) 183.6, 158.9, 157.9, 135.7, 130.7, 130.5, 129.2, 125.0, 81.6, 78.4, 48.9, 27.6, 23.5, 14.7. HRMS (ESI): *m*/*z* [M + Na]^+^ calcd for C_14_H_12_ClNO_3_Na: 300.0398; found: 300.0398.
*8-Chloro-2a,2a^1^,3,4,5,5a-hexahydro-11H-2,6-dioxa-1-azadibenzo[cd,g]azulen-11-one* (**2d**), (silica gel: 200–300 mesh, solvent system: petroleum ether/ethyl acetate = 10:1–5:1), 24 mg, 79%, white solid, m.p.: 179–181 °C. ^1^H NMR (500 MHz, CDCl_3_) *δ* (ppm) 7.94 (d, *J* = 8.5 Hz, 1H), 7.24 (dd, *J* = 8.5, 2.0 Hz, 1H), 7.15 (d, *J* = 2.0 Hz, 1H), 4.87 (dt, *J* = 10.5, 4.4 Hz, 1H), 4.33 (ddd, *J* = 7.1, 6.1, 4.0 Hz, 1H), 3.71 (dd, *J* = 10.4, 7.1 Hz, 1H), 2.2 –2.04 (m, 2H), 1.93–1.79 (m, 2H), 1.78–1.60 (m, 2H). ^13^C NMR (101 MHz, CDCl_3_) *δ* (ppm) 183.6, 161.2, 158.0, 141.9, 131.0, 128.1, 125.5, 123.7, 81.3, 78.5, 49.3, 27.7, 23.7, 14.7. HRMS (ESI): *m*/*z* [M + H]^+^ calcd for C_14_H_13_ClNO_3_: 278.0578; found: 278.0580.
*9-Fluoro-2a,2a^1^,3,4,5,5a-hexahydro-11H-2,6-dioxa-1-azadibenzo[cd,g]azulen-11-one* (**2e**), (silica gel: 200–300 mesh, solvent system: petroleum ether/ethyl acetate = 10:1–5:1), 33 mg, 88%, white solid, m.p.: 132–133 °C. ^1^H NMR (500 MHz, CDCl_3_) *δ* (ppm) 7.62 (dd, *J* = 8.3, 3.2 Hz, 1H), 7.30–7.22 (m, 1H), 7.11 (dd, *J* = 8.9, 4.4 Hz, 1H), 4.88 (dt, *J* = 10.4, 4.3 Hz, 1H), 4.29 (td, *J* = 6.7, 4.0 Hz, 1H), 3.70 (dd, *J* = 10.4, 7.3 Hz, 1H), 2.23–2.00 (m, 2H), 1.93–1.78 (m, 3H), 1.81–1.54 (m, 3H). ^13^C NMR (126 MHz, CDCl_3_) *δ* (ppm) 183.9, 159.2 (d, *J*_c–f_ = 246.8 Hz), 158.0, 156.7 (d, *J*_c–f_ = 2.7 Hz), 130.8 (d, *J*_c–f_ = 7.2 Hz), 125.1 (d, *J*_c–f_ = 8.1 Hz), 123.0 (d, *J*_c–f_ = 23.3 Hz), 115.4 (d, *J*_c–f_ = 24.1 Hz), 81.5, 78.3, 48.8, 27.7, 23.6, 14.7. HRMS (ESI): *m*/*z* [M + H]^+^ calcd for C_14_H_13_FNO_3_: 262.0874; found: 262.0873.
*9-Bromo-2a,2a^1^,3,4,5,5a-hexahydro-11H-2,6-dioxa-1-azadibenzo[cd,g]azulen-11-one* (**2f**), (silica gel: 200–300 mesh, solvent system: petroleum ether/ethyl acetate = 10:1–5:1), 25 mg, 79%, white solid, m.p.: 134–135 °C. ^1^H NMR (500 MHz, CDCl_3_) *δ* (ppm) 8.07 (d, *J* = 2.6 Hz, 1H), 7.64 (dd, *J* = 8.6, 2.6 Hz, 1H), 7.01 (d, *J* = 8.6 Hz, 1H), 4.88 (dt, *J* = 10.4, 4.4 Hz, 1H), 4.30 (td, *J* = 6.8, 4.0 Hz, 1H), 3.70 (dd, *J* = 10.4, 7.2 Hz, 1H), 2.21–2.00 (m, 2H), 1.93–1.80 (m, 2H), 1.79–1.57 (m, 3H). ^13^C NMR (126 MHz, CDCl_3_) *δ* (ppm) 183.5, 159.4, 157.8, 138.7, 132.4, 131.1, 125.3, 118.0, 81.6, 78.3, 49.0, 27.6, 23.6, 14.8. HRMS (ESI): *m*/*z* [M + H]^+^ calcd for C_14_H_13_BrNO_3_: 322.0073; found: 322.0073.
*10-Methoxy-2a,2a^1^,3,4,5,5a-hexahydro-11H-2,6-dioxa-1-azadibenzo[cd,g]azulen-11-one* (**2g**), (silica gel: 200–300 mesh, solvent system: petroleum ether/ethyl acetate = 10:1–5:1), 38 mg, 91%, white solid, m.p.: 194–195 °C. ^1^H NMR (500 MHz, CDCl_3_) *δ* (ppm) 7.43 (t, *J* = 8.3 Hz, 1H), 6.80 (dd, *J* = 8.5, 0.9 Hz, 1H), 6.73 (dd, *J* = 8.1, 0.8 Hz, 1H), 4.92 (dt, *J* = 10.5, 4.4 Hz, 1H), 4.25 (ddd, *J* = 10.7, 7.6, 4.9 Hz, 1H), 3.86 (s, 3H), 3.64 (dd, *J* = 10.5, 7.6 Hz, 1H), 2.10–1.96 (m, 2H), 1.95–1.78 (m, 2H), 1.77–1.52 (m, 2H). ^13^C NMR (126 MHz, CDCl_3_) *δ* (ppm) 183.0, 159.2, 158.2, 157.4, 134.6, 122.0, 115.0, 108.7, 83.4, 78.0, 56.3, 47.4, 26.4, 22.5, 16.2. HRMS (ESI): *m*/*z* [M + H]^+^ calcd for C_15_H_16_NO_4_: 274.1074; found: 274.1074.
*9-Methoxy-2a,2a^1^,3,4,5,5a-hexahydro-11H-2,6-dioxa-1-azadibenzo[cd,g]azulen-11-one* (**2h**), (silica gel: 200–300 mesh, solvent system: petroleum ether/ethyl acetate = 10:1–5:1), 29 mg, 81%, white solid, m.p.: 137–139 °C. ^1^H NMR (500 MHz, CDCl_3_) *δ* (ppm) 7.41 (d, *J* = 3.2 Hz, 1H), 7.11 (dd, *J* = 8.8, 3.2 Hz, 1H), 7.04 (d, *J* = 8.8 Hz, 1H), 4.85 (dt, *J* = 10.3, 4.3 Hz, 1H), 4.23 (ddd, *J* = 7.3, 6.1, 4.1 Hz, 1H), 3.83 (s, 3H), 3.66 (dd, *J* = 10.4, 7.3 Hz, 1H), 2.23–2.01 (m, 2H), 1.93–1.78 (m, 2H), 1.75–1.55 (m, 2H). ^13^C NMR (101 MHz, CDCl_3_) *δ* (ppm) 184.9, 158.4, 156.5, 154.9, 130.0, 124.4, 123.8, 111.2, 81.3, 78.1, 55.8, 49.1, 27.9, 23.7, 14.8. HRMS (ESI): *m*/*z* [M + H]^+^ calcd for C_15_H_16_NO_4_: 274.1074; found: 274.1072.
*8-Methoxy-2a,2a^1^,3,4,5,5a-hexahydro-11H-2,6-dioxa-1-azadibenzo[cd,g]azulen-11-one* (**2i**), (silica gel: 200–300 mesh, solvent system: petroleum ether/ethyl acetate = 10:1–5:1), 26 mg, 81%, light yellow solid, m.p.: 98–100 °C. ^1^H NMR (500 MHz, CDCl_3_) *δ* (ppm) 7.99 (d, *J* = 8.8 Hz, 1H), 6.77 (dd, *J* = 8.9, 2.5 Hz, 1H), 6.56 (d, *J* = 2.4 Hz, 1H), 4.80 (dt, *J* = 10.4, 4.5 Hz, 1H), 4.28 (ddd, *J* = 7.0, 5.0, 3.9 Hz, 1H), 3.85 (s, 3H), 3.70 (dd, *J* = 10.4, 7.0 Hz, 1H), 2.16 (ddq, *J* = 18.8, 14.5, 4.9 Hz, 2H), 1.92–1.73 (m, 2H), 1.72–1.56 (m, 2H). ^13^C NMR (126 MHz, CDCl_3_) *δ* (ppm) 183.3, 166.2, 163.7, 158.6, 131.6, 122.3, 111.9, 107.1, 80.3, 77.9, 55.8, 50.0, 28.1, 24.1, 14.4. HRMS (ESI): *m*/*z* [M + H]^+^ calcd for C_15_H_16_NO_4_: 274.1074; found: 274.1074.
*2a,2a^1^,3,4,5,5a-Hexahydro-13H-2,6-dioxa-1-azabenzo[cd]naphtho[2,1-g]azulen-13-one* (**2j**), (silica gel: 200–300 mesh, solvent system: petroleum ether/ethyl acetate = 10:1–5:1), 29 mg, 86%, brown solid, m.p.: 147–150 °C. ^1^H NMR (500 MHz, CDCl_3_) *δ* (ppm) 8.65 (d, *J* = 8.7 Hz, 1H), 7.99 (d, *J* = 8.8 Hz, 1H), 7.82 (dd, *J* = 8.2, 1.3 Hz, 1H), 7.60 (ddd, *J* = 8.5, 6.7, 1.4 Hz, 1H), 7.49 (ddd, *J* = 8.0, 6.8, 1.2 Hz, 1H), 7.23 (d, *J* = 8.8 Hz, 1H), 4.91 (dt, *J* = 10.5, 4.2 Hz, 1H), 4.34 (ddd, *J* = 9.0, 7.6, 4.8 Hz, 1H), 3.63 (dd, *J* = 10.5, 7.6 Hz, 1H), 2.07 (dddd, *J* = 26.6, 13.3, 10.1, 5.4 Hz, 2H), 1.91 (dddd, *J* = 15.2, 13.3, 7.4, 3.8 Hz, 2H), 1.85–1.54 (m, 2H). ^13^C NMR (126 MHz, CDCl_3_) *δ* (ppm) 186.1, 158.3, 158.0, 136.0, 131.3, 131.0, 129.0, 128.3, 126.0, 126.0, 124.9, 122.0, 83.1, 78.0, 47.5, 26.9, 22.7, 15.9. HRMS (ESI): *m*/*z* [M + H]^+^ calcd for C_18_H_16_NO_3_: 294.1124; found: 294.1123.
*Ethyl 9-oxo-3,3a-dihydro-9H-chromeno[3,2-c]isoxazole-3-carboxylate* (**4a**), (silica gel: 200–300 mesh, solvent system: petroleum ether/ethyl acetate = 5:1–2:1), 35 mg, 95%, yellow oil. ^1^H NMR (500 MHz, CDCl_3_) *δ* (ppm) 8.04 (dd, *J* = 7.9, 1.8 Hz, 1H), 7.61 (ddd, *J* = 8.7, 7.2, 1.8 Hz, 1H), 7.19 (ddd, *J* = 8.1, 7.2, 1.0 Hz, 1H), 7.09 (dd, *J* = 8.4, 1.0 Hz, 1H), 6.03 (d, *J* = 6.9 Hz, 1H), 5.35 (d, *J* = 6.9 Hz, 1H), 4.36 (q, *J* = 7.1 Hz, 2H), 1.37 (t, *J* = 7.1 Hz, 3H). ^13^C NMR (126 MHz, CDCl_3_) *δ* 174.4, 166.2, 159.2, 151.4, 137.6, 128.0, 123.6, 123.4, 118.7, 86.7, 85.3, 63.1, 14.0. HRMS (ESI): *m*/*z* [M + H]^+^ calcd for C_13_H_12_NO_5_: 274.0710; found: 274.0712.
*Ethyl 7-methyl-9-oxo-3,3a-dihydro-9H-chromeno[3,2-c]isoxazole-3-carboxylate* (**4b**), (silica gel: 200–300 mesh, solvent system: petroleum ether/ethyl acetate = 5:1–2:1), 31 mg, 91%, yellow solid, m.p.: 129–130 °C. ^1^H NMR (500 MHz, CDCl_3_) *δ* (ppm) 7.83–7.77 (m, 1H), 7.41 (dd, *J* = 8.5, 2.4 Hz, 1H), 6.98 (d, *J* = 8.4 Hz, 1H), 5.98 (d, *J* = 7.0 Hz, 1H), 5.32 (d, *J* = 7.0 Hz, 1H), 4.35 (q, *J* = 7.1 Hz, 2H), 2.35 (s, 3H), 1.37 (t, *J* = 7.1 Hz, 3H). ^13^C NMR (126 MHz, CDCl_3_) *δ* 174.5, 166.3, 157.3, 151.6, 138.7, 133.4, 127.4, 123.0, 118.4, 86.7, 85.2, 63.0, 20.4, 14.0. HRMS (ESI): *m*/*z* [M + H]^+^ calcd for C_14_H_14_NO_5_: 276.0867; found: 276.0867.
*Ethyl 6-methyl-9-oxo-3,3a-dihydro-9H-chromeno[3,2-c]isoxazole-3-carboxylate* (**4c**), (silica gel: 200–300 mesh, solvent system: petroleum ether/ethyl acetate = 5:1–2:1), 26 mg, 82%, white solid, m.p.: 95–97 °C. ^1^H NMR (500 MHz, CDCl_3_) *δ* (ppm) 7.88 (d, *J* = 8.1 Hz, 1H), 6.98 (dd, *J* = 8.2, 1.5 Hz, 1H), 6.86 (s, 1H), 5.97 (d, *J* = 7.0 Hz, 1H), 5.30 (d, *J* = 7.0 Hz, 1H), 4.34 (q, *J* = 7.1 Hz, 2H), 2.39 (s, 3H), 1.36 (t, *J* = 7.2 Hz, 3H). ^13^C NMR (126 MHz, CDCl_3_)) *δ* (ppm) 174.0, 166.3, 159.2, 151.5, 149.8, 127.7, 124.9, 121.1, 118.6, 86.7, 85.1, 63.0, 22.0, 14.0. HRMS (ESI): *m*/*z* [M + H]^+^ calcd for C_14_H_14_NO_5_: 276.0867; found: 274.0867.
*Ethyl 7-methoxy-9-oxo-3,3a-dihydro-9H-chromeno[3,2-c]isoxazole-3-carboxylate* (**4d**), 30 mg, 86%, yellow solid, m.p.: 167–168 °C. ^1^H NMR (500 MHz, CDCl_3_) *δ* (ppm) 7.40 (d, *J* = 3.1 Hz, 1H), 7.19 (dd, *J* = 9.1, 3.2 Hz, 1H), 7.01 (d, *J* = 9.1 Hz, 1H), 5.97 (d, *J* = 7.0 Hz, 1H), 5.31 (d, *J* = 7.1 Hz, 1H), 4.35 (q, *J* = 7.1 Hz, 2H), 3.83 (s, 3H), 1.37 (t, *J* = 7.1 Hz, 3H). ^13^C NMR (126 MHz, CDCl_3_) *δ* (ppm) 174.3, 166.3, 155.6, 153.9, 151.7, 126.9, 123.5, 120.0, 107.8, 86.8, 85.2, 63.1, 55.9, 14.1. HRMS (ESI): *m*/*z* [M + H]^+^ calcd for C_14_H_14_NO_6_: 292.0816; found: 292.0816.
*Ethyl 6-methoxy-9-oxo-3,3a-dihydro-9H-chromeno[3,2-c]isoxazole-3-carboxylate* (**4e**), 29 mg, 87%, white solid, m.p.: 120–121 °C. ^1^H NMR (500 MHz, CDCl_3_) *δ* (ppm) 7.93 (d, *J* = 8.9 Hz, 1H), 6.71 (dd, *J* = 8.9, 2.4 Hz, 1H), 6.49 (d, *J* = 2.3 Hz, 1H), 5.98 (d, *J* = 7.1 Hz, 1H), 5.28 (d, *J* = 7.2 Hz, 1H), 4.34 (q, *J* = 7.1 Hz, 2H), 3.86 (s, 3H), 1.36 (t, *J* = 7.2 Hz, 3H). ^13^C NMR (126 MHz, CDCl_3_) *δ* (ppm) 172.9, 167.3, 166.3, 161.4, 151.4, 129.6, 117.1, 111.9, 101.7, 86.9, 84.9, 63.0, 55.9, 14.0. HRMS (ESI): *m*/*z* [M + H]^+^ calcd for C_14_H_14_NO_6_: 292.0816; found: 292.0817.
*Ethyl 7-bromo-9-oxo-3,3a-dihydro-9H-chromeno[3,2-c]isoxazole-3-carboxylate* (**4f**), 24 mg, 80%, yellow solid, m.p.: 139–142 °C. ^1^H NMR (500 MHz, CDCl_3_) *δ* (ppm) 11.76 (s, 1H), 8.72 (d, *J* = 2.5 Hz, 1H), 7.66 (d, *J* = 11.4 Hz, 1H), 7.41 (s, 1H), 6.98 (d, *J* = 8.9 Hz, 1H), 4.49 (q, *J* = 7.1 Hz, 2H), 1.45 (t, *J* = 7.1 Hz, 3H). ^13^C NMR (126 MHz, CDCl_3_) δ (ppm) 187.3, 163.0, 161.8, 161.5, 156.0, 140.7, 135.3, 120.5, 119.5, 111.4, 110.0, 62.9, 14.1. HRMS (ESI): *m*/*z* [M + H]^+^ calcd for C_13_H_11_BrNO_5_: 339.9815; found: 339.9817.
*Ethyl 7-chloro-9-oxo-3,3a-dihydro-9H-chromeno[3,2-c]isoxazole-3-carboxylate* (**4g**), 27 mg, 83%, yellow solid, m.p.: 142–144 °C. ^1^H NMR (500 MHz, CDCl_3_) *δ* (ppm) 7.97 (d, *J* = 2.7 Hz, 1H), 7.54 (dd, *J* = 8.9, 2.7 Hz, 1H), 7.05 (d, *J* = 8.9 Hz, 1H), 6.03 (d, *J* = 6.9 Hz, 1H), 5.35 (d, *J* = 6.9 Hz, 1H), 4.36 (q, *J* = 7.2 Hz, 2H), 1.37 (t, *J* = 7.2 Hz, 3H). ^13^C NMR (126 MHz, CDCl_3_) *δ* (ppm) 173.3, 166.0, 157.6, 150.8, 137.4, 129.3, 127.1, 124.1, 120.4, 86.7, 85.4, 63.2, 14.0. HRMS (ESI): *m*/*z* [M + H]^+^ calcd for C_13_H_11_ClNO_5_: 296.0320; found: 296.0320.
*Ethyl 7-fluoro-9-oxo-3,3a-dihydro-9H-chromeno[3,2-c]isoxazole-3-carboxylate* (**4h**), 20 mg, 78%, white solid, m.p.: 149–151 °C. ^1^H NMR (500 MHz, CDCl_3_) *δ* (ppm) 7.66 (dd, *J* = 8.0, 3.1 Hz, 1H), 7.32 (ddd, *J* = 9.0, 7.4, 3.2 Hz, 1H), 7.08 (dd, *J* = 9.1, 4.1 Hz, 1H), 6.02 (d, *J* = 6.9 Hz, 1H), 5.34 (d, *J* = 6.9 Hz, 1H), 4.35 (q, *J* = 7.1 Hz, 2H), 1.37 (t, *J* = 7.1 Hz, 3H). ^13^C NMR (126 MHz, CDCl_3_) *δ* (ppm) 173.6, 166.0, 159.2 (d, *J*_c–f_ = 246.1 Hz), 155.4, 151.0, 125.1 (d, *J*_c–f_ = 24.8 Hz), 124.0 (d, *J*_c–f_ = 7.3 Hz), 120.5 (d, *J*_c–f_ = 7.4 Hz), 113.1 (d, *J*_c–f_ = 24.5 Hz), 86.8, 85.3, 63.1, 14.0. HRMS (ESI): *m*/*z* [M + H]^+^ calcd for C_13_H_11_FNO_5_: 280.0616; found: 280.0616.
*Ethyl 11-oxo-7a,8-dihydro-11H-benzo[5,6]chromeno[3,2-c]isoxazole-8-carboxylate* (**4i**), 14 mg, 70%, yellow solid, m.p.: 194–195 °C. ^1^H NMR (500 MHz, CDCl_3_) *δ* (ppm) 9.47 (d, *J* = 8.7 Hz, 1H), 8.04 (d, *J* = 9.0 Hz, 1H), 7.81 (d, *J* = 8.1 Hz, 1H), 7.76–7.70 (m, 1H), 7.53 (t, *J* = 7.5 Hz, 1H), 7.16 (d, *J* = 9.0 Hz, 1H), 6.09 (d, *J* = 6.9 Hz, 1H), 5.40 (d, *J* = 6.9 Hz, 1H), 4.38 (q, *J* = 7.1 Hz, 2H), 1.39 (t, *J* = 7.1 Hz, 3H). ^13^C NMR (126 MHz, CDCl_3_) *δ* (ppm) 175.0, 166.3, 161.8, 152.1, 139.5, 131.4, 130.7, 129.9, 128.7, 126.3, 126.2, 118.5, 115.7, 86.4, 85.3, 63.1, 14.1. HRMS (ESI): *m*/*z* [M + H]^+^ calcd for C_17_H_14_NO_5_: 312.0866; found: 312.0866.
*Methyl 9-oxo-3,3a-dihydro-9H-chromeno[3,2-c]isoxazole-3-carboxylate* (**4j**), 29 mg, 90%, white solid, m.p.: 138–139 °C. ^1^H NMR (400 MHz, CDCl_3_) *δ* (ppm) 8.04 (dd, *J* = 7.9, 1.8 Hz, 1H), 7.61 (ddd, *J* = 8.4, 7.2, 1.8 Hz, 1H), 7.19 (ddd, *J* = 8.1, 7.2, 1.1 Hz, 1H), 7.08 (dd, *J* = 8.4, 1.1 Hz, 1H), 6.03 (d, *J* = 6.9 Hz, 1H), 5.37 (d, *J* = 7.0 Hz, 1H), 3.91 (s, 3H). ^13^C NMR (101 MHz, CDCl_3_) *δ* 174.3, 166.6, 159.1, 151.4, 137.6, 1287.0, 123.6, 123.4, 118.7, 86.7, 85.1, 53.6. HRMS (ESI): *m*/*z* [M + H]^+^ calcd for C_12_H_10_NO_5_: 248.0554; found: 248.0553.
*Methyl 7-methyl-9-oxo-3,3a-dihydro-9H-chromeno[3,2-c]isoxazole-3-carboxylate* (**4k**), 32 mg, 92%, yellow solid, m.p.: 160–162 °C. ^1^H NMR (400 MHz, CDCl_3_) *δ* (ppm) 7.80 (d, *J* = 2.4 Hz, 1H), 7.40 (dd, *J* = 8.5, 2.4 Hz, 1H), 6.97 (d, *J* = 8.5 Hz, 1H), 5.98 (d, *J* = 7.2 Hz, 1H), 5.34 (d, *J* = 7.1 Hz, 1H), 3.90 (s, 3H), 2.35 (s, 3H). ^13^C NMR (101 MHz, CDCl_3_) *δ* (ppm) 174.4, 166.8, 157.3, 151.6, 138.7, 133.4, 127.4, 123.0, 118.4, 86.7, 85.0, 53.5, 20.4. HRMS (ESI): *m*/*z* [M + H]^+^ calcd for C_13_H_12_NO_5_: 262.0710; found: 262.0710.
*Methyl 7-methoxy-9-oxo-3,3a-dihydro-9H-chromeno[3,2-c]isoxazole-3-carboxylate* (**4l**), 28 mg, 89%, yellow solid, m.p.: 193–194 °C. ^1^H NMR (500 MHz, CDCl_3_) *δ* (ppm) 7.41 (d, *J* = 3.2 Hz, 1H), 7.20 (dd, *J* = 9.1, 3.2 Hz, 1H), 7.01 (d, *J* = 9.0 Hz, 1H), 5.98 (d, *J* = 7.2 Hz, 1H), 5.34 (d, *J* = 7.2 Hz, 1H), 3.91 (s, 3H), 3.84 (s, 3H). ^13^C NMR (126 MHz, CDCl_3_) *δ* (ppm) 174.2, 166.8, 155.6, 153.9, 151.7, 126.9, 1235, 120.0, 107.8, 86.8, 85.0, 55.9, 53.6. HRMS (ESI): *m*/*z* [M + H]^+^ calcd for C_13_H_12_NO_6_: 278.0659; found: 278.0657.
*Methyl 6-chloro-9-oxo-3,3a-dihydro-9H-chromeno[3,2-c]isoxazole-3-carboxylate* (**4m**), 29 mg, 89%, yellow solid, m.p.: 121–123 °C. ^1^H NMR (500 MHz, CDCl_3_) *δ* (ppm) 11.97 (d, *J* = 1.5 Hz, 1H), 8.55 (dd, *J* = 8.8, 1.5 Hz, 1H), 7.43 (d, *J* = 1.4 Hz, 1H), 7.08 (t, *J* = 1.8 Hz, 1H), 6.98 (dd, *J* = 8.8, 2.0 Hz, 1H), 4.03 (d, *J* = 1.5 Hz, 3H). ^13^C NMR (126 MHz, CDCl_3_) *δ* (ppm) 187.2, 164.6, 162.1, 161.0, 156.5, 144.4, 134.4, 120.5, 118.5, 117.0, 110.3, 53.3. HRMS (ESI): *m*/*z* [M + H]^+^ calcd for C_12_H_9_ClNO_5_: 282.0164; found: 282.0164.
*Methyl 7-chloro-9-oxo-3,3a-dihydro-9H-chromeno[3,2-c]isoxazole-3-carboxylate* (**4n**), 29 mg, 89%, yellow solid, m.p.: 176–178 °C. ^1^H NMR (500 MHz, CDCl_3_) *δ* (ppm) 7.98 (d, *J* = 2.7 Hz, 1H), 7.55 (dd, *J* = 8.9, 2.6 Hz, 1H), 7.05 (d, *J* = 8.8 Hz, 1H), 6.03 (d, *J* = 7.0 Hz, 1H), 5.38 (d, *J* = 6.9 Hz, 1H), 3.91 (s, 3H). ^13^C NMR (126 MHz, CDCl_3_) *δ* (ppm) 173.3, 166.5, 157.6, 150.9, 137.4, 129.4, 127.2, 124.1, 120.4, 86.8, 85.2, 53.7. HRMS (ESI): *m*/*z* [M + H]^+^ calcd for C_12_H_9_ClNO_5_: 282.0164; found: 282.0164.
*Methyl 11-oxo-7a,8-dihydro-11H-benzo[5,6]chromeno[3,2-c]isoxazole-8-carboxylate* (**4o**), 30 mg, 86%, light yellow solid, m.p.: 145–146 °C. ^1^H NMR (500 MHz, CDCl_3_) *δ* (ppm) 7.68 (dd, *J* = 7.9, 3.1 Hz, 1H), 7.33 (ddd, *J* = 9.1, 7.4, 3.2 Hz, 1H), 7.09 (dd, *J* = 9.1, 4.1 Hz, 1H), 6.03 (d, *J* = 6.9 Hz, 1H), 5.38 (d, *J* = 6.9 Hz, 1H), 3.92 (s, 3H). ^13^C NMR (126 MHz, CDCl_3_) *δ* (ppm) 173.5, 166.5, 159.3, 155.4, 151.0, 125.3, 124.1, 120.56, 113.2, 86.8, 85.2, 53.7. HRMS (ESI): *m*/*z* [M + H]^+^ calcd for C_12_H_9_FNO_5_: 266.0459; found: 266.0459.
*Methyl 7-bromo-9-oxo-3,3a-dihydro-9H-chromeno[3,2-c]isoxazole-3-carboxylate* (**4p**), 18 mg, 77%, yellow solid, m.p.: 173–174 °C. ^1^H NMR (500 MHz, CDCl_3_) *δ* (ppm) 8.13 (d, *J* = 2.6 Hz, 1H), 7.68 (dd, *J* = 8.8, 2.6 Hz, 1H), 6.99 (d, *J* = 8.8 Hz, 1H), 6.03 (d, *J* = 6.9 Hz, 1H), 5.38 (d, *J* = 6.9 Hz, 1H), 3.91 (s, 3H). ^13^C NMR (126 MHz, CDCl_3_) *δ* (ppm) 173.2, 166.4, 158.0, 150.8, 140.2, 130.3, 124.5, 120.6, 116.5, 86.7, 85.2, 53.7. HRMS (ESI): *m*/*z* [M + H]^+^ calcd for C_12_H_9_BrNO_5_: 325.9658; found: 325.9658.
*Methyl 11-oxo-7a,8-dihydro-11H-benzo[5,6]chromeno[3,2-c]isoxazole-8-carboxylate* (**4q**), 20 mg, 67%, yellow solid, m.p.: 183–184 °C. ^1^H NMR (500 MHz, CDCl_3_) *δ* 9.48 (d, *J* = 8.7 Hz, 1H), 8.05 (d, *J* = 8.9 Hz, 1H), 7.82 (d, *J* = 8.1 Hz, 1H), 7.73 (ddd, *J* = 8.6, 6.9, 1.5 Hz, 1H), 7.54 (t, *J* = 7.5 Hz, 1H), 7.17 (d, *J* = 9.2 Hz, 1H), 6.10 (d, *J* = 6.9 Hz, 1H), 5.43 (d, *J* = 6.8 Hz, 1H), 3.93 (s, 3H). ^13^C NMR (126 MHz, Chloroform-*d*) *δ* 174.9, 166.8, 161.8, 152.1, 139.5, 131.4, 130.8, 129.9, 128.7, 126.3, 126.2, 118.4, 115.8, 86.42, 9.12, 53.6. HRMS (ESI): *m*/*z* [M + H]^+^ calcd for C_16_H_12_NO_5_: 298.07100; found:298.07100.
*3,3a,4,5-Tetrahydro-11H-benzo[7,8]oxocino[5,4-c]isoxazol-11-one* (**4r**), 22 mg, 78%, light brown solid, m.p.: 178–179 °C. 1H NMR (400 MHz, CDCl3) δ (ppm) 7.78 (dd, J = 7.8, 1.7 Hz, 1H), 7.55 (ddd, J = 8.1, 7.4, 1.8 Hz, 1H), 7.21 (td, J = 7.5, 1.0 Hz, 1H), 7.11 (dd, J = 8.2, 1.0 Hz, 1H), 5.08 (dd, J = 8.5, 4.7 Hz, 1H), 4.57 (dt, J = 9.4, 2.7 Hz, 1H), 3.83 (ddd, J = 12.0, 9.7, 2.3 Hz, 1H), 3.45 (ddd, J = 14.7, 8.5, 1.2 Hz, 1H), 3.32 (dd, J = 14.5, 0.9 Hz, 1H), 2.03–1.78 (m, 2H). 13C NMR (101 MHz, CDCl3) δ (ppm) 190.4, 161.2, 159.0, 135.5, 132.4, 128.7, 124.2, 121.4, 79.4, 70.3, 41.8, 34.5. HRMS (ESI): *m*/*z* [M + H]+ calcd for C_12_H_12_NO_3_: 218.0811; found: 218.0813.
*4H,10H-Benzo[6,7]oxepino[4,3-c]isoxazol-10-one* (**6a**), 22 mg, 82%, white solid, m.p.: 166–167 °C. ^1^H NMR (500 MHz, CDCl_3_) *δ* (ppm) 8.57 (s, 1H), 8.26 (dd, *J* = 8.1, 1.8 Hz, 1H), 7.58 (ddd, *J* = 8.5, 7.2, 1.8 Hz, 1H), 7.31–7.23 (m, 1H), 7.16 (dd, *J* = 8.1, 1.2 Hz, 1H), 5.15 (s, 2H). ^13^C NMR (126 MHz, CDCl_3_) *δ* (ppm) 180.2, 160.1, 155.6, 136.3, 132.1, 127.7, 124.5, 122.8, 117.5, 63.6. HRMS (ESI): *m*/*z* [M + H]^+^ calcd for C_11_H_8_NO_3_: 202.0498; found: 202.0498.
*Methyl-4H,10H-benzo[6,7]oxepino[4,3-c]isoxazol-10-one* (**6b**), 15 mg, 75%, light yellow solid, m.p.: 193–196 °C. ^1^H NMR (500 MHz, CDCl_3_) *δ* (ppm) 8.56 (s, 1H), 8.02 (d, *J* = 2.5 Hz, 1H), 7.37 (dd, *J* = 8.3, 2.4 Hz, 1H), 7.05 (d, *J* = 8.2 Hz, 1H), 5.10 (s, 2H), 2.37 (s, 3H). ^13^C NMR (126 MHz, CDCl_3_) *δ* (ppm) 180.4, 160.0, 158.0, 155.6, 137.2, 134.2, 131.8, 127.4, 122.6, 117.7, 63.6, 20.5. HRMS (ESI): *m*/*z* [M + H]^+^ calcd for C_12_H_10_NO_3_: 216.0655; found: 216.0655.
*7-Methyl-4H,10H-benzo[6,7]oxepino[4,3-c]isoxazol-10-one* (**6c**), 10 mg, 68%, white solid, m.p.: 196–197 °C. ^1^H NMR (500 MHz, CDCl_3_) *δ* (ppm) 8.56 (s, 1H), 8.02 (d, *J* = 2.5 Hz, 1H), 7.37 (dd, *J* = 8.3, 2.4 Hz, 1H), 7.05 (d, *J* = 8.2 Hz, 1H), 5.10 (s, 2H), 2.37 (s, 3H). ^13^C NMR (126 MHz, CDCl_3_) *δ* (ppm) 179.7, 160.2, 160.1, 155.5, 148.2, 132.2, 125.6, 125.1, 122.9, 117.4, 63.4, 21.5. HRMS (ESI): *m*/*z* [M + H]^+^ calcd for C_12_H_10_NO_3_: 216.0655; found: 216.0654.
*8-Methoxy-4H,10H-benzo[6,7]oxepino[4,3-c]isoxazol-10-one* (**6d**), 32 mg, 71%, yellow solid, m.p.: 157–159 °C. ^1^H NMR (500 MHz, CDCl_3_) *δ* (ppm) 8.56 (s, 1H), 7.66 (d, *J* = 3.2 Hz, 1H), 7.14 (dd, *J* = 8.9, 3.1 Hz, 1H), 7.09 (d, *J* = 8.9 Hz, 1H), 5.15–5.04 (m, 2H), 3.86 (s, 3H). ^13^C NMR (126 MHz, CDCl_3_) *δ* (ppm) 180.1, 159.7, 156.1, 155.6 154.3, 128.3, 124.4, 124.1, 117.8, 113.1, 63.8, 55.9. HRMS (ESI): *m*/*z* [M + H]^+^ calcd for C_12_H_10_NO_4_: 232.0604; found: 232.0605.
*7-Methoxy-4H,10H-benzo[6,7]oxepino[4,3-c]isoxazol-10-one* (**6e**), 20 mg, 74%, white solid, m.p.: 206–207 °C. ^1^H NMR (500 MHz, CDCl_3_) *δ* (ppm) 8.56 (s, 1H), 8.27 (d, *J* = 9.0 Hz, 1H), 6.80 (dd, *J* = 9.1, 2.5 Hz, 1H), 6.59 (d, *J* = 2.5 Hz, 1H), 5.12 (d, *J* = 0.7 Hz, 2H), 3.88 (s, 3H). ^13^C NMR (126 MHz, CDCl_3_) *δ* (ppm) 178.5, 166.2, 162.3, 160.4, 155.5, 134.3, 120.7, 117.0, 111.8, 106.1, 63.3, 55.8. HRMS (ESI): *m*/*z* [M + H]^+^ calcd for C_12_H_10_NO_4_: 232.0604; found: 232.0604.
*Fluoro-4H,10H-benzo[6,7]oxepino[4,3-c]isoxazol-10-one* (**6f**), 15 mg, 62%, white solid, m.p.: 187–189 °C. ^1^H NMR (500 MHz, CDCl_3_) *δ* (ppm) 8.59 (s, 1H), 7.91 (dd, *J* = 9.2, 3.3 Hz, 1H), 7.31–7.26 (m, 1H), 7.16 (dd, *J* = 8.9, 4.6 Hz, 1H), 5.13 (s, 2H). ^13^C NMR (126 MHz, CDCl_3_) *δ* (ppm) 179.1, 159.4, 159.4 (d, *J*_c–f_ = 245.3 Hz), 156.2 (d, *J*_c–f_ = 2.6 Hz), 155.8, 128.9 (d, *J*_c–f_ = 7.3 Hz), 124.7 (d, *J*_c–f_ = 7.4 Hz), 123.5 (d, *J*_c–f_ = 23.3 Hz), 117.5 (d, *J*_c–f_ = 24.9 Hz), 63.8. HRMS (ESI): *m*/*z* [M + H]^+^ calcd for C_11_H_7_FNO_3_: 220.0405; found: 220.0404.
*8-Chloro-4H,10H-benzo[6,7]oxepino[4,3-c]isoxazol-10-one* (**6g**), 20 mg, 70%, white solid, m.p.: 202–203 °C. ^1^H NMR (500 MHz, CDCl_3_) *δ* (ppm) 8.59 (s, 1H), 8.22 (d, *J* = 2.8 Hz, 1H), 7.51 (dd, *J* = 8.7, 2.8 Hz, 1H), 7.13 (d, *J* = 8.7 Hz, 1H), 5.14 (s, 2H). ^13^C NMR (126 MHz, CDCl_3_) *δ* (ppm) 178.9, 159.6, 158.6, 155.9, 136.1, 131.4, 130.2, 128.5, 124.6, 117.2, 63.7. HRMS (ESI): *m*/*z* [M + H]^+^ calcd for C_11_H_7_ClNO_3_: 236.0109; found: 236.0109.
*8-Bromo-4H,10H-benzo[6,7]oxepino[4,3-c]isoxazol-10-one* (**6h**), 31 mg, 83%, white solid, m.p.: 193–194 °C. ^1^H NMR (500 MHz, CDCl_3_) *δ* (ppm) 8.59 (s, 1H), 8.36 (d, *J* = 2.6 Hz, 1H), 7.65 (dd, *J* = 8.7, 2.6 Hz, 1H), 7.06 (d, *J* = 8.6 Hz, 1H), 5.14 (s, 2H). ^13^C NMR (126 MHz, CDCl_3_) *δ* (ppm) 178.8, 159.6, 159.1, 155.9, 139.9, 134.4, 128.8, 124.8, 117.5, 117.2, 63.6. HRMS (ESI): *m*/*z* [M + H]^+^ calcd for C_11_H_7_BrNO_3_: 279.9603; found: 279.9602.
*8H,12H-Naphtho[1′,2′:6,7]oxepino[4,3-c]isoxazol-12-one* (**6i**), 27 mg, 85%, light brown solid, m.p.: 156–158 °C. ^1^H NMR (500 MHz, CDCl_3_) *δ* (ppm) 8.53–8.42 (m, 2H), 7.98 (d, *J* = 8.8 Hz, 1H), 7.82 (dd, *J* = 8.1, 1.4 Hz, 1H), 7.59 (ddd, *J* = 8.7, 6.9, 1.5 Hz, 1H), 7.49 (ddd, *J* = 8.1, 6.9, 1.2 Hz, 1H), 7.28 (d, *J* = 8.8 Hz, 1H), 5.18 (d, *J* = 1.2 Hz, 2H). ^13^C NMR (126 MHz, CDCl_3_) *δ* (ppm) 184.2, 159.9, 158.1, 154.8, 135.9, 131.6, 131.5, 128.8, 128.3, 126.5, 126.1, 125.5, 121.6, 117.8, 65.2. HRMS (ESI): *m*/*z* [M + H]^+^ calcd for C_15_H_10_NO_3_: 252.0655; found: 252.0653.
*1-(2-(But-3-en-1-yloxy)phenyl)ethan-1-one* (**3r**), 24 mg, 67%, light yellow oil. ^1^H NMR (400 MHz, CDCl_3_) *δ* (ppm) 7.73 (dd, *J* = 7.7, 1.9 Hz, 1H), 7.43 (ddd, *J* = 8.3, 7.3, 1.9 Hz, 1H), 7.05–6.84 (m, 2H), 5.90 (ddt, *J* = 16.9, 10.2, 6.6 Hz, 1H), 5.30–5.02 (m, 2H), 4.12 (t, *J* = 6.4 Hz, 2H), 2.63–2.57 (m, 5H). ^13^C NMR (101 MHz, CDCl_3_) *δ* (ppm) 199.8, 158.2, 134.3, 133.6, 130.4, 128.2, 120.5, 117.4, 112.1, 67.6, 33.6, 32.1. HRMS (ESI): *m*/*z* [M + H]^+^ calcd for C_12_H_15_NO_2_: 191.1066; found: 191.1066.
*Ethyl 3-benzoyl-4,5-dihydroisoxazole-5-carboxylate* (**10**), 27 mg, 58%, light yellow oil, m.p.: 156–158 °C. ^1^H NMR (400 MHz, CDCl_3_) *δ* (ppm) 8.25–8.16 (m, 2H), 7.60 (td, *J* = 7.3, 1.5 Hz, 1H), 7.47 (td, *J* = 7.9, 1.7 Hz, 2H), 5.22–5.14 (m, 1H), 4.32–4.22 (m, 2H), 3.73–3.56 (m, 2H), 1.32 (td, *J* = 7.1, 1.6 Hz, 3H). ^13^C NMR (101 MHz, CDCl_3_) *δ* (ppm) 185.4, 169.1, 156.8, 135.4, 133.8, 130.3, 128.4, 78.9, 62.2, 38.4, 14.0. HRMS (ESI): *m*/*z* [M + H]^+^ calcd for C_13_H_14_NO_4_: 248.0917; found: 248.0918.
*(E)-2-Oxo-2-phenylacetaldehyde oxime* (**11**), 24 mg, 67%, light yellow solid, m.p.: 150–151 °C. ^1^H NMR (400 MHz, CDCl_3_) *δ* (ppm) 8.68 (s, 1H), 8.07–8.04 (m, 2H), 8.03 (d, *J* = 1.4 Hz, 1H), 7.64–7.58 (m, 1H), 7.48 (dd, *J* = 8.4, 7.2 Hz, 2H). ^13^C NMR (101 MHz, CDCl_3_) *δ* (ppm) 188.6, 148.7, 135.8, 133.6, 129.9, 128.5. HRMS (ESI): *m*/*z* [M + H]^+^ calcd for C_8_H_8_NO_2_: 150.0550; found: 150.0550.

## 4. Conclusions

In summary, an effective and metal-free method for the synthesis of 6/7/8-membered ketone-fused isoxazoles/isoxazolines tetra- or tricyclic compounds was developed while employing TBN as a radical initiator and NO source. In this protocol, TBN activated the C_sp_^3^–H bond of aryl methyl ketones to produce α-carbonyl nitrile oxide intermediates in situ through cascade Hydrogen Atom Transfer (HAT) and the radical coupling process, which then underwent [3 + 2] cycloaddition with alkenyl/alkynyl groups. The present approach overcomes the entropic effects and ring strain associated with the conventional synthesis of densely fused polycyclic compounds. The protocol has a wide substrate scope and diverse possible products, with the additional merits of being metal-catalyst-free and additive-free.

## Data Availability

The data presented in this study are available in article and Supplementary Materials.

## Supplementary Materials

The following supporting information can be downloaded at https://www.mdpi.com/article/10.3390/molecules29061202/s1. X-ray crystal structure and crystallographic data; optimization of reaction conditions; and characterization data for product **2a–2j**, **4a–4r**, and **6a–6i**, including ^1^H-NMR and ^13^C-NMR.

## References

[1] Morita T., Fukuhara S., Fuse S., Nakamura H. (2018). Gold(I)-Catalyzed Intramolecular SEAr Reaction: Efficienct Synthesis of Isoxazole-Containing Fused Heterocycles. Org. Lett..

[2] Ibrahim K.T., Neetha M., Anilkumar G. (2022). Advancements in the synthesis of oxazolines. Monatsh. Chem..

[3] Jiang B., Dai M. (2023). Concise Total Syntheses of the 6–7–5 Hamigeran Natural Products. J. Am. Chem. Soc..

[4] Jiang B., Dai M. (2020). Synthetic Studies toward the Hamigerans with a 6–7–5 Tricyclic Core. Org. Lett..

[5] Guo Y., Xiang Y., Wei L., Wan J.-P. (2018). Thermoinduced Free-Radical C–H Acyloxylation of Tertiary Enaminones: Catalyst-Free Synthesis of Acyloxyl Chromones and Enaminones. Org. Lett..

[6] Lin Y., Wan J.-P., Liu Y. (2023). Cascade in Situ Iodination, Chromone Annulation, and Cyanation for Site-Selective Synthesis of 2-Cyanochromones. J. Org. Chem..

[7] Luo T., Wan J.-P., Liu Y. (2020). Toward C2-nitrogenated chromones by copper-catalyzed β-C(sp2)–H N-heteroarylation of enaminones. Org. Chem. Front..

[8] Yu Q., Liu Y., Wan J.-P. (2020). Transition metal-free synthesis of 3-trifluoromethyl chromones via tandem C–H trifluoromethylation and chromone annulation of enaminones. Org. Chem. Front..

[9] Haji M., Hosseinzadeh M. (2022). Cyclohepta[b]pyran: An important scaffold in biologically active natural products. Med. Chem. Res..

[10] Liu Y.-W., Wang M.-M., Zhang Y.-Q., Xu H., Dai H.-X. (2023). Construction of Indole-Fused Seven- and Eight-Membered Azaheterocycles via a Tandem Pd/NBE-Catalyzed Decarbonylation and Dual C–H Activation Sequence. Org. Lett..

[11] Osipyan A., Sapegin A., Novikov A.S., Krasavin M. (2018). Rare Medium-Sized Rings Prepared via Hydrolytic Imidazoline Ring Expansion (HIRE). J. Org. Chem..

[12] Gao M., Gan Y., Xu B. (2019). From Alkenes to Isoxazolines via Copper-Mediated Alkene Cleavage and Dipolar Cycloaddition. Org. Lett..

[13] Meng L., Liu H., Lin Z., Wang J. (2022). Synthetic and Computational Study of the Enantioselective [3 + 2]-Cycloaddition of Chromones with MBH Carbonates. Org. Lett..

[14] Krstić G., Saidu M.B., Bombicz P., De S., Ali H., Zupkó I., Berkecz R., Gallah U.S., Rédei D., Hohmann J. (2023). Pauciflorins A–E, Unexpected Chromone–Monoterpene-Derived Meroterpenoids from Centrapalus pauciflorus. J. Nat. Prod..

[15] Rao B., Tang J., Wei Y., Zeng X. (2016). Ring Expansion via Cleavage of Benzylic C−C Bonds Enabling Direct Synthesis of Medium Ring-Fused Benzocarbocycles. Chem. Asian J..

[16] Wang J., Qi T., He S., Huang W., Peng C., Zhan G., Han B. (2023). Catalyst-Controlled Switchable (5 + 4)/(3 + 4) Cycloadditions for the Divergent Synthesis of Pyrazole-Fused Seven- and Nine-Membered Heterocycles. ACS Catal..

[17] Reyes R.L., Iwai T., Sawamura M. (2021). Construction of Medium-Sized Rings by Gold Catalysis. Chem. Rev..

[18] Pan G., Lu L., Zhuang W., Huang Q. (2021). Synthesis of Indole-Fused Six-, Seven-, or Eight-Membered N,O-Heterocycles via Rhodium-Catalyzed NH-Indole-Directed C–H Acetoxylation/Hydrolysis/Annulation. J. Org. Chem..

[19] Li Q.-Z., Guan Y.-L., Huang Q.-W., Qi T., Xiang P., Zhang X., Leng H.-J., Li J.-L. (2023). Temperature-Controlled Divergent Asymmetric Synthesis of Indole-Based Medium-Sized Heterocycles through Palladium Catalysis. ACS Catal..

[20] Li P., Jia X. (2018). *tert*-Butyl Nitrite (TBN) as a Versatile Reagent in Organic Synthesis. Synthesis.

[21] Khaligh G.N. (2020). Recent Advances and Applications of *tert*-Butyl Nitrite (TBN) in Organic Synthesis. Mini-Rev. Org. Chem..

[22] Dahiya A., Sahoo A.K., Alam T., Patel B.K. (2019). *tert*-Butyl Nitrite (TBN), a Multitasking Reagent in Organic Synthesis. Chem. Asian J..

[23] Guo X., Xu G., Zhou L., Yan H., Hao X.-Q., Wang Q. (2020). Synthesis and application of α-carbonyl nitrile oxides. Org. Chem. Front..

[24] Pan J., Li X., Lin F., Liu J., Jiao N. (2018). Chemoselective Nitrosylation of Anilines and Alkynes via Fragmentary or Complete NO Incorporation. Chem.

[25] Wang X.-D., Zhu L.-H., Liu P., Wang X.-Y., Yuan H.-Y., Zhao Y.-L. (2019). Copper-Catalyzed Cascade Cyclization Reactions of Diazo Compounds with *tert*-Butyl Nitrite and Alkynes: One-Pot Synthesis of Isoxazoles. J. Org. Chem..

[26] Xiong M., Liang X., Gao Z., Lei A., Pan Y. (2019). Synthesis of Isoxazolines and Oxazines by Electrochemical Intermolecular [2 + 1 + n] Annulation: Diazo Compounds Act as Radical Acceptors. Org. Lett..

[27] Tang Z., Zhou Y., Song Q. (2019). Synthesis of Furoxans and Isoxazoles via Divergent [2 + 1 + 1 + 1] Annulations of Sulfoxonium Ylides and tBuONO. Org. Lett..

[28] Dai P., Tan X., Luo Q., Yu X., Zhang S., Liu F., Zhang W.-H. (2019). Synthesis of 3-Acyl-isoxazoles and Δ^2^-Isoxazolines from Methyl Ketones, Alkynes or Alkenes, and *tert*-Butyl Nitrite via a Csp3–H Radical Functionalization/Cycloaddition Cascade. Org. Lett..

[29] Ma L., Jin F., Cheng X., Tao S., Jiang G., Li X., Yang J., Bao X., Wan X. (2021). [2 + 2 + 1] Cycloaddition of N-tosylhydrazones, *tert*-butyl nitrite and alkenes: A general and practical access to isoxazolines. Chem. Sci..

[30] Chen R., Zhao Y., Fang S., Long W., Sun H., Wan X. (2017). Coupling Reaction of Cu-Based Carbene and Nitroso Radical: A Tandem Reaction to Construct Isoxazolines. Org. Lett..

[31] Chen R., Ogunlana A.A., Fang S., Long W., Sun H., Bao X., Wan X. (2018). In situ generation of nitrile oxides from copper carbene and *tert*-butyl nitrite: Synthesis of fully substituted isoxazoles. Org. Biomol. Chem..

[32] Song W., Liu Y., Yan N., Wan J.-P. (2023). Tunable Key [3 + 2] and [2 + 1] Cycloaddition of Enaminones and α-Diazo Compounds for the Synthesis of Isomeric Isoxazoles: Metal-Controlled Selectivity. Org. Lett..

[33] Ma L., Kou L., Jin F., Cheng X., Tao S., Jiang G., Bao X., Wan X. (2021). Acyclic nitronate olefin cycloaddition (ANOC): Regio- and stereospecific synthesis of isoxazolines. Chem. Sci..

[34] Sudoh Y., Jin Z.-T., Imafuku K., Matsumura H. (1982). Reactions of 3-acetyltropolone and its methyl ethers with hydroxylamine. Formation of 8H-cyclohept[d]isoxazol-8-one and 8H-cyclohept[c]isoxazol-8-one. J. Heterocycl. Chem..

[35] Wang Z., Zhao Y., Chen J., Chen M., Li X., Jiang T., Liu F., Yang X., Sun Y., Zhu Y. (2023). One-Pot Synthesis of Isoxazole-Fused Tricyclic Quinazoline Alkaloid Derivatives via Intramolecular Cycloaddition of Propargyl-Substituted Methyl Azaarenes under Metal-Free Conditions. Molecules.

[36] Zhao P., Wu X., Zhou Y., Geng X., Wang C., Wu Y.-d., Wu A.-X. (2019). Direct Synthesis of 2,3-Diaroyl Quinolines and Pyridazino[4,5-b]quinolines via an I2-Promoted One-Pot Multicomponent Reaction. Org. Lett..

[37] Huang H.-M., Bellotti P., Daniliuc C.G., Glorius F. (2021). Radical Carbonyl Propargylation by Dual Catalysis. Angew. Chem. Int. Ed..

[38] Choudhury A.R., Manna M.S., Mukherjee S. (2017). Nitro-enabled catalytic enantioselective formal umpolung alkenylation of β-ketoesters. Chem. Sci..

[39] Zhang X.-J., Wang Z., Zhang H., Gao J.-J., Yang K.-R., Fan W.-Y., Wu R.-X., Feng M.-L., Zhu W., Zhu Y.-P. (2022). Iodine-Mediated Domino Cyclization for One-Pot Synthesis of Indolizine-Fused Chromones via Metal-Free sp3 C–H Functionalization. J. Org. Chem..

[40] Shang Z.-H., Zhang X.-J., Li Y.-M., Wu R.-X., Zhang H.-R., Qin L.-Y., Ni X., Yan Y., Wu A.-X., Zhu Y.-P. (2021). One-Pot Synthesis of Chromone-Fused Pyrrolo[2,1-a]isoquinolines and Indolizino[8,7-b]indoles: Iodine-Promoted Oxidative [2 + 2 + 1] Annulation of O-Acetylphenoxyacrylates with Tetrahydroisoquinolines and Noreleagnines. J. Org. Chem..

[41] Ma X., Dang H., Rose J.A., Rablen P., Herzon S.B. (2017). Hydroheteroarylation of Unactivated Alkenes Using N-Methoxyheteroarenium Salts. J. Am. Chem. Soc..

[42] Sutariya T.R., Labana B.M., Parmar B.D., Parmar N.J., Kant R., Gupta V.K. (2015). A domino synthetic approach for new, angular pyrazol- and isoxazol-heterocycles using [DBU][Ac] as an effective reaction medium. RSC Adv..

[43] Shaikh M.H., Subhedar D.D., Khedkar V.M., Jha P.C., Khan F.A.K., Sangshetti J.N., Shingate B.B. (2016). 1,2,3-Triazole tethered acetophenones: Synthesis, bioevaluation and molecular docking study. Chin. Chem. Lett..

[44] Purushothaman S., Prasanna R., Raghunathan R. (2013). Regioselective synthesis of spiropyrrolidine/spiropyrrolizidine/spirothiazolidine-grafted macrocycles through 1,3-dipolar cycloaddition methodology. Tetrahedron.

[45] Dimirjian C.A., Castiñeira Reis M., Balmond E.I., Turman N.C., Rodriguez E.P., Di Maso M.J., Fettinger J.C., Tantillo D.J., Shaw J.T. (2019). Synthesis of Spirobicyclic Pyrazoles by Intramolecular Dipolar Cycloadditions/[1s, 5s] Sigmatropic Rearrangements. Org. Lett..

[46] James M.J., Schwarz J.L., Strieth-Kalthoff F., Wibbeling B., Glorius F. (2018). Dearomative Cascade Photocatalysis: Divergent Synthesis through Catalyst Selective Energy Transfer. J. Am. Chem. Soc..

