# Supplementary material for: tert-Butyl Nitrite-Induced Radical Nitrile Oxidation Cycloaddition: Synthesis of Isoxazole/Isoxazoline-Fused Benzo 6/7/8-membered Oxacyclic Ketones
Source: Molecules. 2024 Mar 7;29(6):1202. doi: 10.3390/molecules29061202 (PMC10974807; doi:10.3390/molecules29061202)
Supplement: Supplementary file 1 [file molecules-29-01202-s001.zip › molecules-2883519-supplementary.pdf]

Supporting Information

tert-Butyl Nitrite-Induced Radical Nitrile Oxidation Cycloaddition: Synthesis of Isoxazole/Isoxazoline-Fused Benzo 6/7/8-membered Oxacyclic Ketones

Jian-Kang Cao [1], Tian-Zheng Cao [1], Qian-Wen Yue [1], Ying Ma [1], Chuan-Ming Yang [1], Hong-Xi Zhang [1], Ya-Chen Li [1], Qiao-Ke Dong [1], Yan-Ping Zhu [1,2,*] and Yuan-Yuan Sun [1,*]

[1] Key Laboratory of Molecular Pharmacology and Drug Evaluation, Ministry of Education, Collaborative Innovation Center of Advanced Drug Delivery System and Biotech Drugs in Universities of Shandong, School of Pharmacy, Yantai University, Yantai 264005, China; cjk18954343136@163.com (J.-K. C.); tianzheng202201@163.com (T.-Z. C.); 19553508187@163.com (Q.-W.Y.); 15935574380@163.com (Y.M.); 15254990262@163.com (C.-M. Y.); 13355081055@163.com (H.-X. Z.); a2732586174@163.com (Y.-C. L.); 19558912885@163.com (Q.-K. D.)

[2] Anhui Laboratory of Molecule-Based Materials, College of Chemistry and Materials Science, Anhui Normal University, Wuhu 241000, China

* Correspondence: chemzyp@ytu.edu.cn (Y.-P. Z.); graceyuanyuan2022@163.com (Y.-Y. S.)

Table of Contents

1. Evidence in support of the hypothetic mechanism 3

2. X-ray crystal structure and crystallographic data 3

3. Experimental Procedures 5

4. Copy of ^{1}H and ^{13}C NMR Spectra of Products 8

1. Evidence in support of the hypothetic mechanism

To gain further insight into the reaction mechanism, some control experiments were performed as illustrated in **Scheme S1**. To address the possible reaction intermediate, 1-(2-(benzyloxy)phenyl)ethan-1-one (**7**) was reacted under the standard conditions for 20 mins, nitrile oxides (**8**) could be detected by MS (APCI) (Schemes S1 and S2).

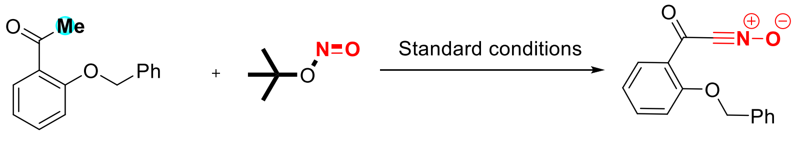

Scheme S1. Control experiment of **7** with TBN.

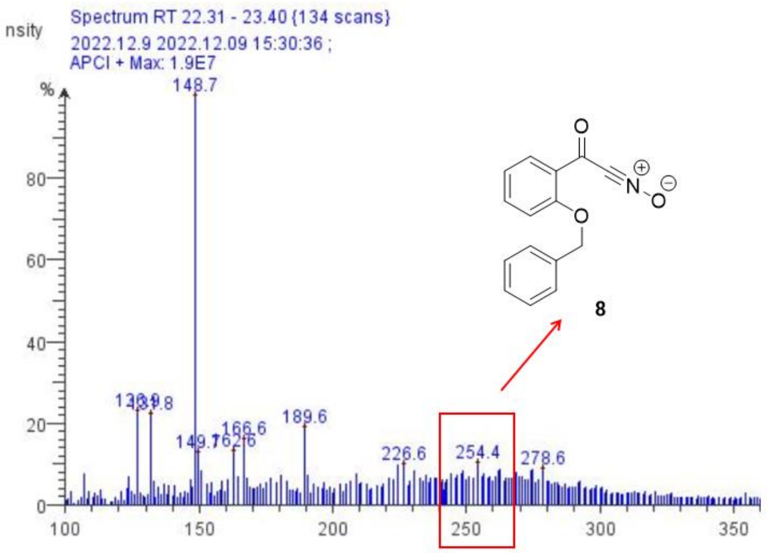

Scheme S2. The MS(APCI) results for the reaction of **7** was reacted under the standard conditions for 20 mins.

2. X-ray crystal structure and crystallographic data

The purified compound **2a** is dissolved in chloroform and petroleum ether, and placed in a dark cabinet to slowly evaporate. After several days, a white bulk crystal is obtained. The X-ray crystal-structure determinations were obtained on a Bruker Smart APEX CCD area detector diffra ctometer at 296(2) K.

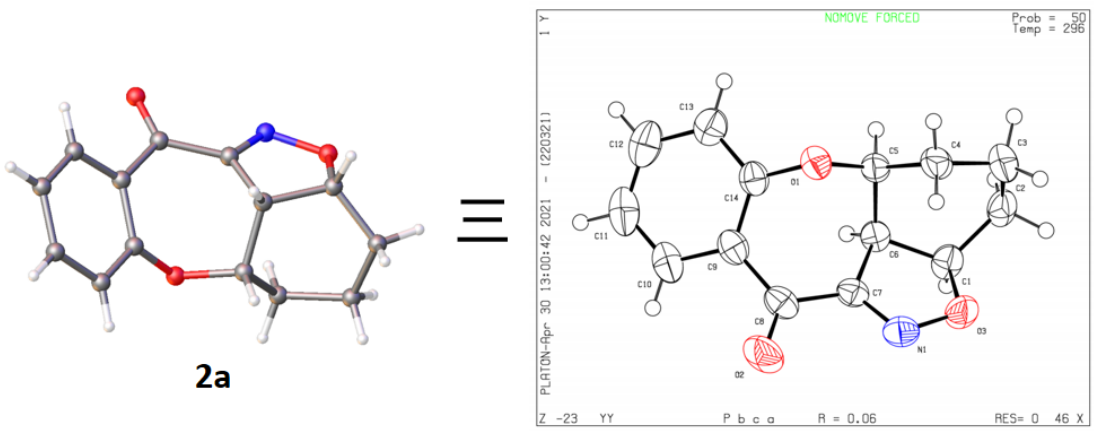

Figure S1. X-ray crystal structure of compound **2a** (displacement ellipsoids are drawn at the 50% probability level).

Table S1. Crystal data and structure refinement for compound **2a**

Identification code	CCDC: 2322778
Empirical formula	$C_{14}H_{13}NO_3$
Formula weight	243.09
Temperature/K	296 (2)
Crystal system	monoclinic
Space group	C2/c
a/Å	10.959 (6)
b/Å	10.915 (7)
c/Å	19.007 (11)
α/°	90
β/°	90
γ/°	90
Volume/Å^3	2274 (2)
Z	8
ρ_{calc}g/cm^3	1.421
μ/mm^{-1}	0.101
F(000)	1024.0

3. Experimental Procedures

3.1 Optimization of reaction conditions for the synthesis of ethyl 9-oxo-3,3a-dihydro-9*H*-chromeno[3,2-*c*]isoxazole-3-carboxylate

To evaluate our proposed assumption, **1a** was selected as the starting material for the optimization of the reaction conditions (**Table S2**). We initiated our investigation by performing the reaction of **1a** with TBN. At first, several different solvents were selected to optimize the reaction. The results showed that only when DMSO was used as solvent to participate in the reaction, the target product was generated. To our delight, DMSO as the solvent at 80 °C for 10 h led to a 95% yield (Table S2, entry 7). Subsequently, different temperature were screened for further increasing the reaction conversion. When the temperature was screened below 80 °C, the yield was significantly reduced (entries 8-10). As a further investigation, we screened the effect of reaction time for this annulation reaction (entries 11-13). After a series of screening, it was found that this reaction is best achieved using **1a** (0.2 mmol), TBN (0.8 mmol) at 80 °C for 10 h (entry 7).

Table S2. Optimization of the reaction conditions for **2a**[a].

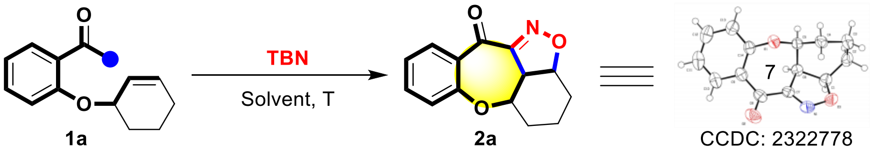

Entry	Solvent	T (°C)	*t*-BuONO	Yield[b] (%)
1	DMSO	80	3.0 equiv	82
2	Toluene	80	3.0 equiv	N.P.
3	Dioxane	80	3.0 equiv	N.P.
4	DEC	80	3.0 equiv	N.P.
5	MeCN	80	3.0 equiv	N.P.
6	DMSO	80	2.0 equiv	78
**7**	**DMSO**	**80**	**4.0 equiv**	**95**
8	DMSO	70	4.0 equiv	83
9	DMSO	60	4.0 equiv	79
10	DMSO	50	4.0 equiv	73
11[c]	DMSO	80	4.0 equiv	78
12[d]	DMSO	80	4.0 equiv	85
13[e]	DMSO	80	4.0 equiv	88

[a] Reaction conditions: **1a** (0.2 mmol) and TBN (0.8 mmol) were heated in DMSO (2

mL) at 80 °C for 10 h. [b] Isolated yields. [c] 6 h. [d] 8 h. [e] 12 h.

3.2 Optimization of reaction conditions for the synthesis of ethyl 9-oxo-3,3a-dihydro-9*H*-chromeno[3,2-*c*]isoxazole-3-carboxylate

Table S3. Optimization of the reaction conditions for **4a**[a].

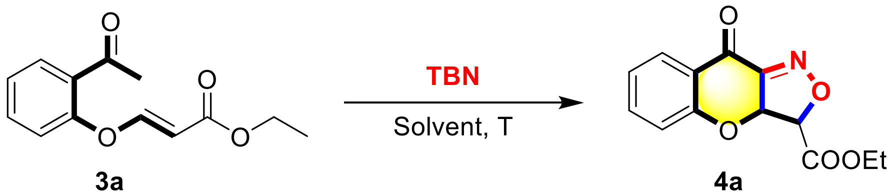

entry	solvent	T (°C)	*t*-BuONO	Yield[b](%)
1	DMSO	100	4.0 equiv.	50
2	DMSO	100	5.0 equiv.	63
3	DMSO	100	6.0 equiv.	65
4	DMSO	100	7.0 equiv.	76
5	DMSO	90	7.0 equiv.	82
**6**	**DMSO**	**80**	**7.0 equiv.**	**95**
7	DMSO	70	7.0 equiv.	87
8[c]	DMSO	80	7.0 equiv.	83
9[d]	DMSO	80	7.0 equiv.	86
10[e]	DMSO	80	7.0 equiv.	89

[a] Reaction conditions: **1a** (0.2 mmol) and TBN (1.4 mmol) were heated in DMSO (2 mL) at 80 °C for 10 h. [b] Isolated yields.[c] 6 h. [d] 8 h. [e]12 h.

We initiated our investigation by performing the reaction of ethyl (*E*)-3-(2-acetylphenoxy)acrylate **3a** with TBN. To our delight, DMSO as the solvent at 80 °C for 10 h led to 50% yield (Table S3, entry 1). Firstly, we optimized the equivalent of TBN in the reaction, and the results showed that increasing the amount of TBN can significantly improve the yield. When TBN reached 7 equiv, the yield of **4a** reached 76%. (Table S3, entry 2-4). To further improve the reactivity, different temperature were screened for further increasing the reaction conversion. When the reaction temperature is 80°C , the yield can reach a maximum of 95% (Table S3, entry 5-7). As a further investigation, we screened the effect of reaction time for this

annulation reaction (Table S3, entries 8-10).After a series of screening, it was found that this reaction is best achieved using **1a** (0.2 mmol), TBN (0.8 mmol) at 80 °C for 10 h (entry 6).

3.3 Optimization of reaction conditions for the synthesis of ethyl 4*H*,10*H*-benzo[6,7]oxepino[4,3-*c*]isoxazol-10-one

Table S4. Optimization of the reaction conditions for **6a**[a].

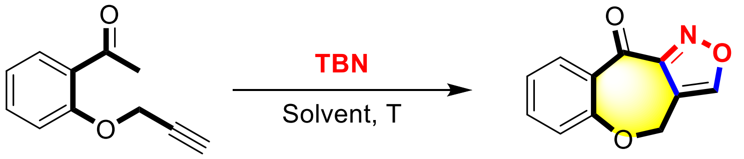

5a 6a

entry	solvent	*T* (°C)	*t*-BuONO	Yield[b](%)
1	DMSO	80	2.0 equiv.	63
2	DMSO	80	3.0 equiv.	70
3	DMSO	80	4.0 equiv.	74
**4**	**DMSO**	**80**	**5.0 equiv.**	**82**
5	DMSO	90	5.0 equiv.	77
6	DMSO	100	5.0 equiv.	72
7[c]	DMSO	80	5.0 equiv.	68
8[d]	DMSO	80	5.0 equiv.	75
9[e]	DMSO	80	5.0 equiv.	76

[a] Reaction conditions: **1a** (0.2 mmol) and TBN (1.0 mmol) were heated in DMSO (2 mL) at 80 °C for 10 h. [b] Isolated yields. [c]6 h. [d] 8 h. [e] 12 h.

We used 1-(2-(prop-2-yn-1-yloxy)phenyl)ethan-1-one **5a** as the starting material to react with TBN in DMSO. Fortunately, the reaction achieved a yield of 63% at 80 °C for 10 h. (Table S4, entry 1). Firstly, we optimized the equivalent of TBN in the reaction, and the results showed that increasing the amount of TBN can significantly improve the yield. When TBN reached 5 equiv, the yield of **6a** reached 82%. (Table S4, entry 2-4). As a further investigation, we screened the effect of reaction temperature and reaction time for this annulation reaction (Table S4, entries 5-9). After a series of screening, it was found that this reaction is best achieved using **1a** (0.2 mmol), TBN (0.8 mmol) at 80 °C for 10 h (entry 4).

4. Copy of 1H and 13C NMR Spectra of Products

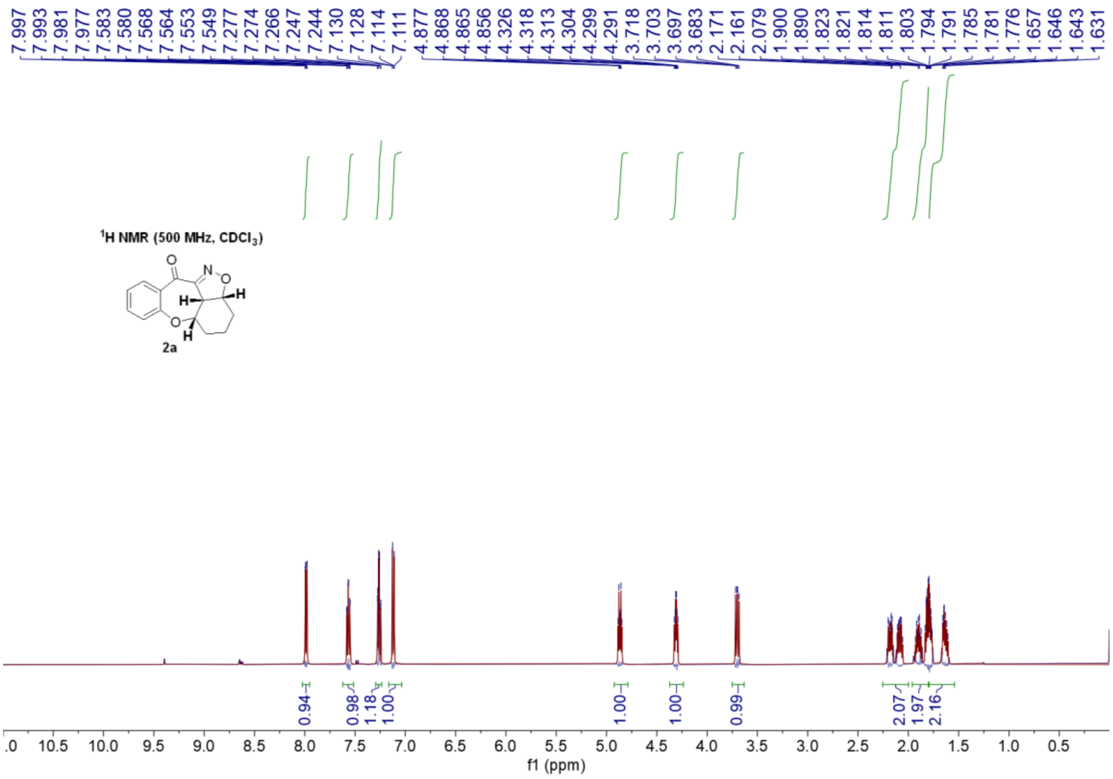

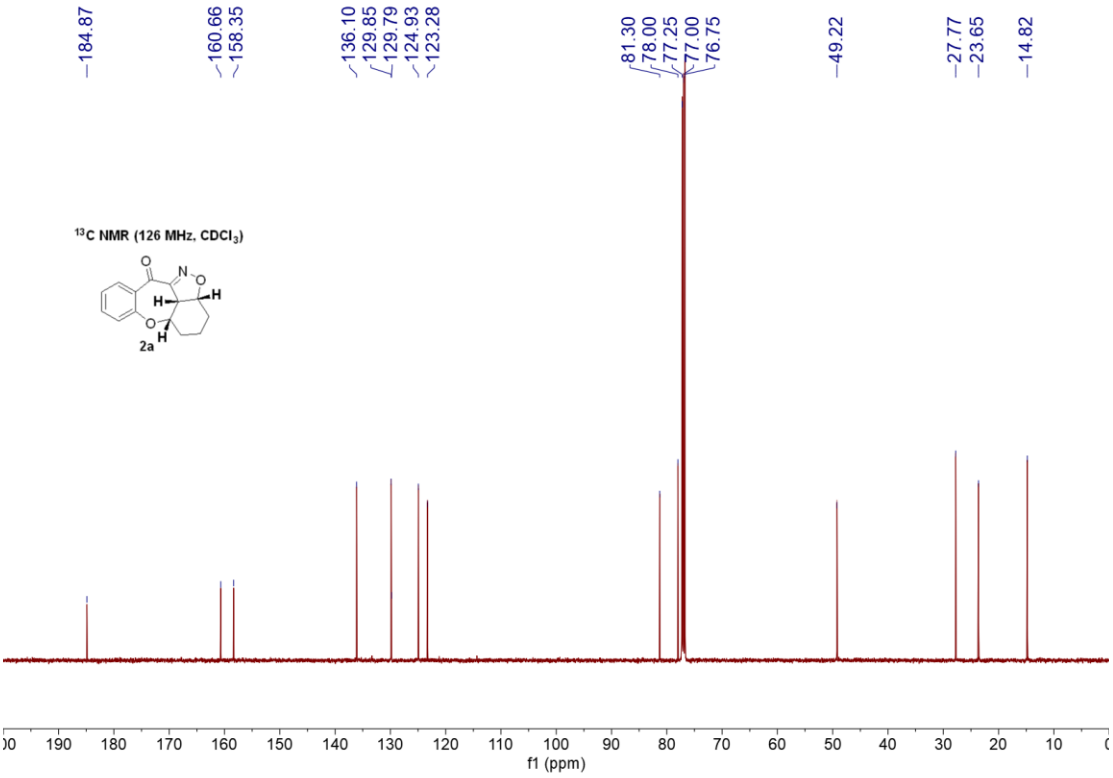

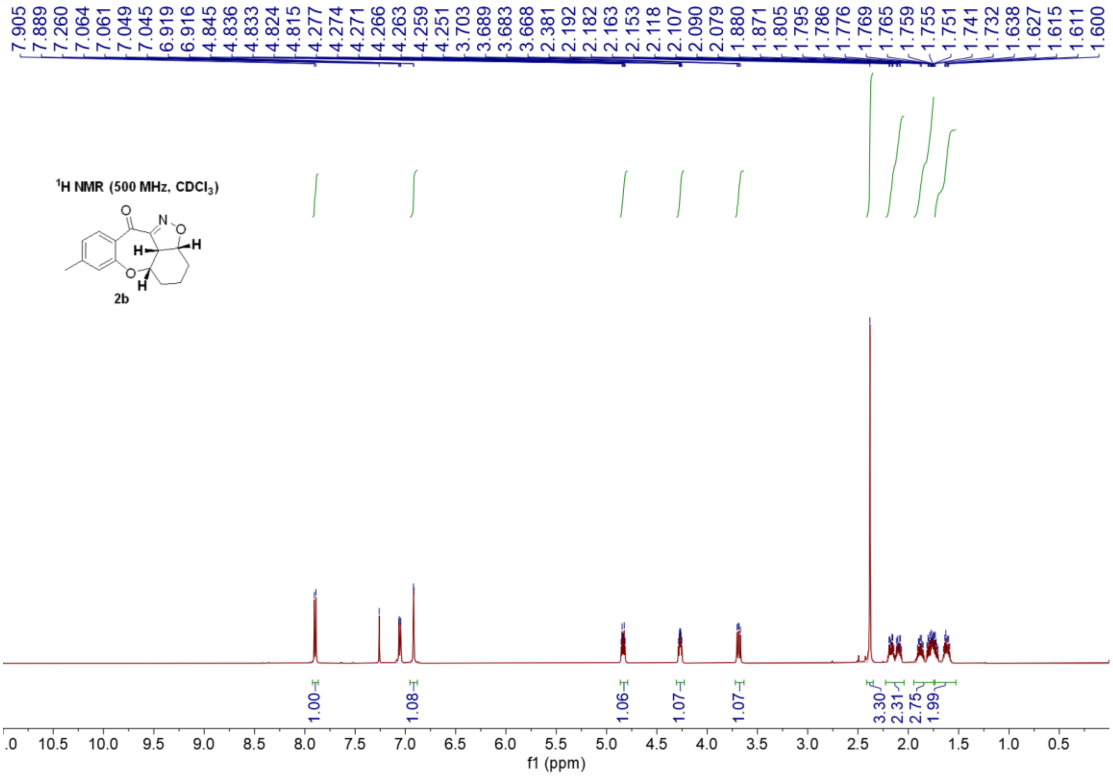

1H NMR (500 MHz, CDCl3)
2b
7.905
7.889
7.260
7.064
7.061
7.049
7.045
6.919
6.916
4.845
4.836
4.833
4.824
4.815
4.277
4.274
4.271
4.266
4.263
4.259
4.251
3.703
3.689
3.683
3.668
2.381
2.192
2.182
2.163
2.153
2.118
2.107
2.090
2.079
1.880
1.871
1.805
1.795
1.786
1.776
1.769
1.765
1.759
1.755
1.751
1.741
1.732
1.638
1.627
1.615
1.611
1.600
1.00
1.08
1.06
1.07
1.07
3.30
2.31
2.75
1.99
f1 (ppm)

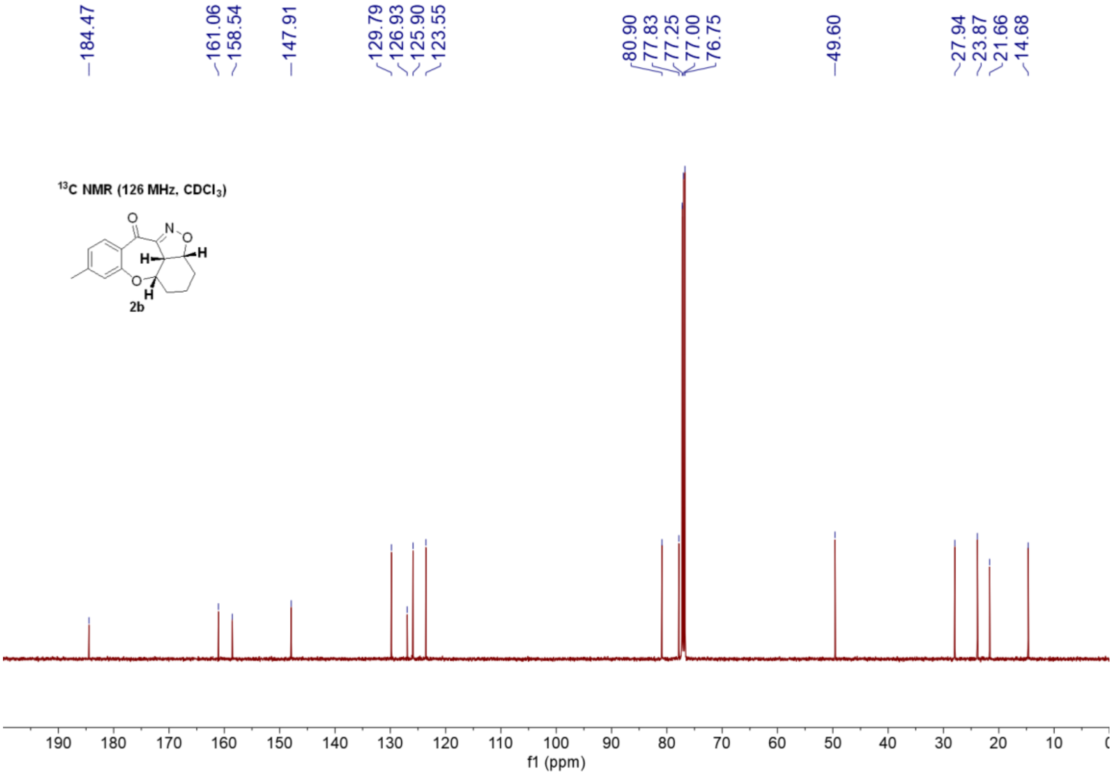

13C NMR (126 MHz, CDCl3)
2b
184.47
161.06
158.54
147.91
129.79
126.93
125.90
123.55
80.90
77.83
77.25
77.00
76.75
49.60
27.94
23.87
21.66
14.68
f1 (ppm)

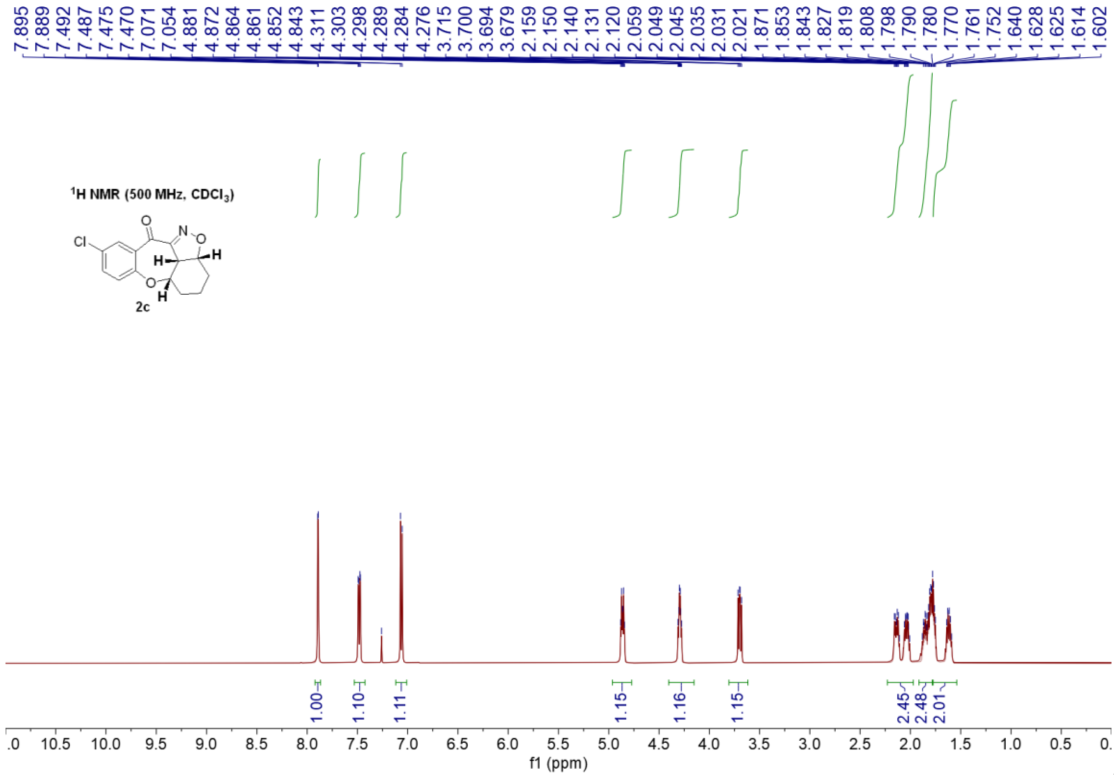

7.895 7.889 7.492 7.487 7.475 7.470 7.071 7.054 4.881 4.872 4.864 4.861 4.852 4.843 4.311 4.303 4.298 4.289 4.284 4.276 3.715 3.700 3.694 3.679 2.159 2.150 2.140 2.131 2.120 2.059 2.049 2.045 2.035 2.031 2.021 1.871 1.853 1.843 1.827 1.819 1.808 1.798 1.790 1.780 1.770 1.761 1.752 1.640 1.628 1.625 1.614 1.602
1H NMR (500 MHz, CDCl3)
2c
1.00 1.10 1.11 1.15 1.16 1.15 2.45 2.48 2.01
f1 (ppm)

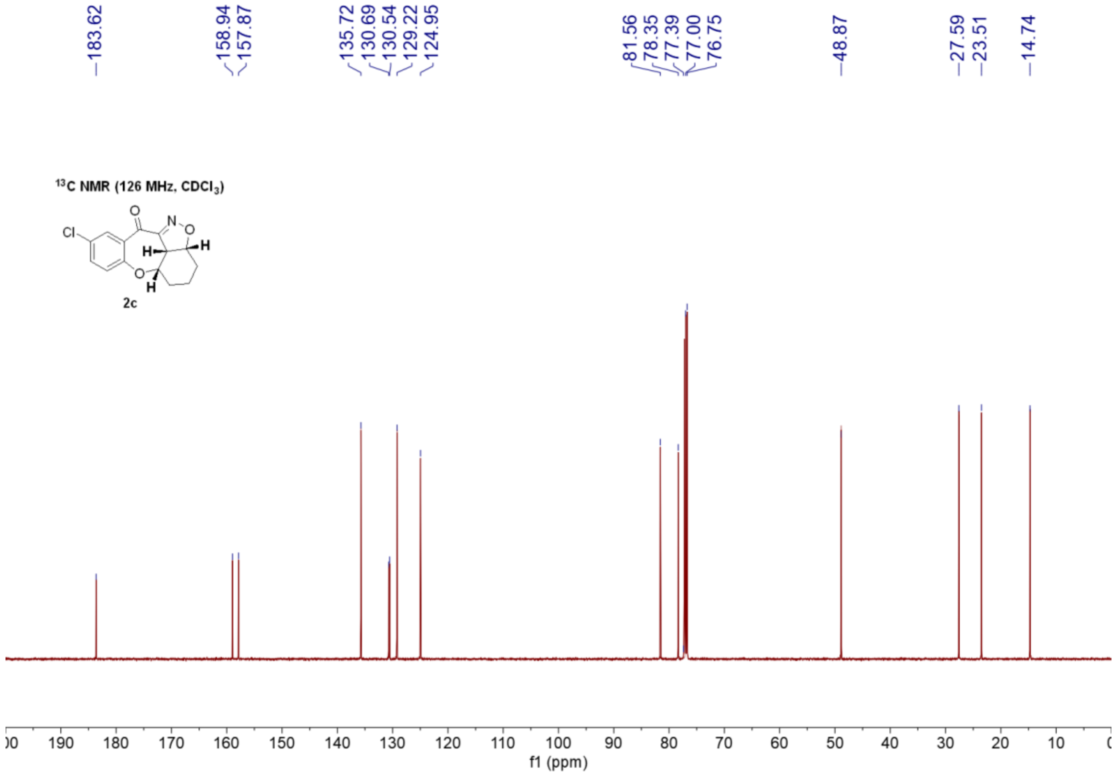

183.62 158.94 157.87 135.72 130.69 130.54 129.22 124.95 81.56 78.35 77.39 77.00 76.75 48.87 27.59 23.51 14.74
13C NMR (126 MHz, CDCl3)
2c
f1 (ppm)

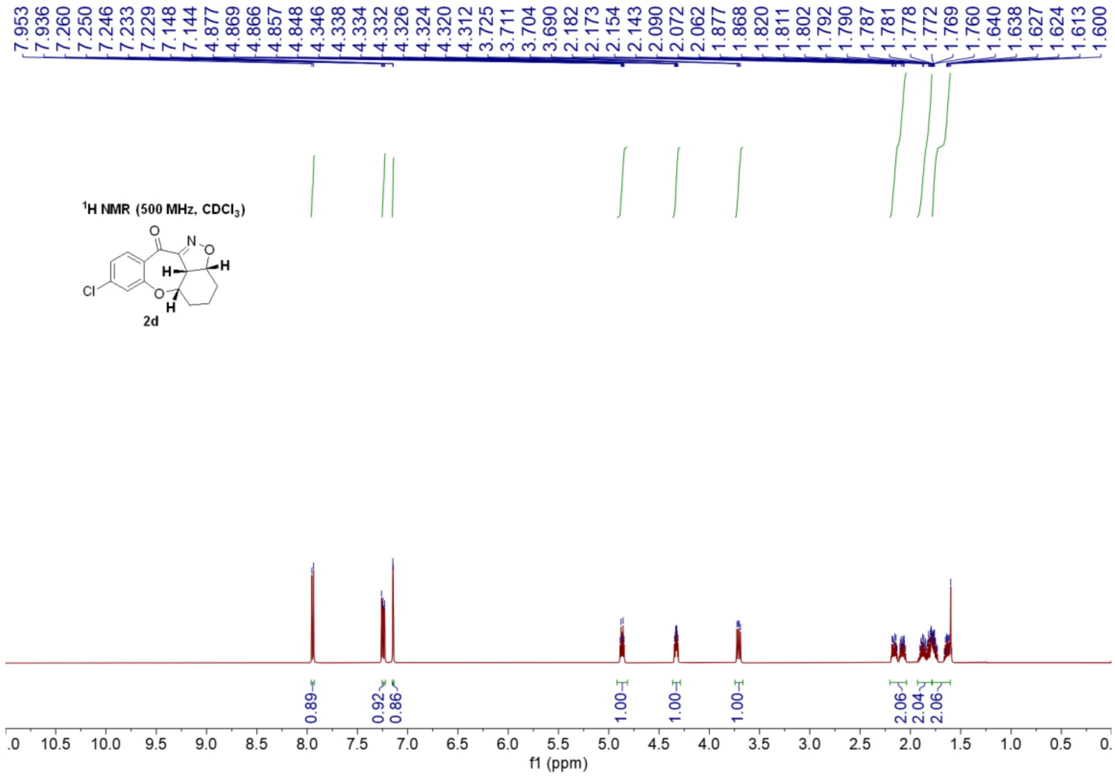

1H NMR (500 MHz, CDCl3)
2d
f1 (ppm)

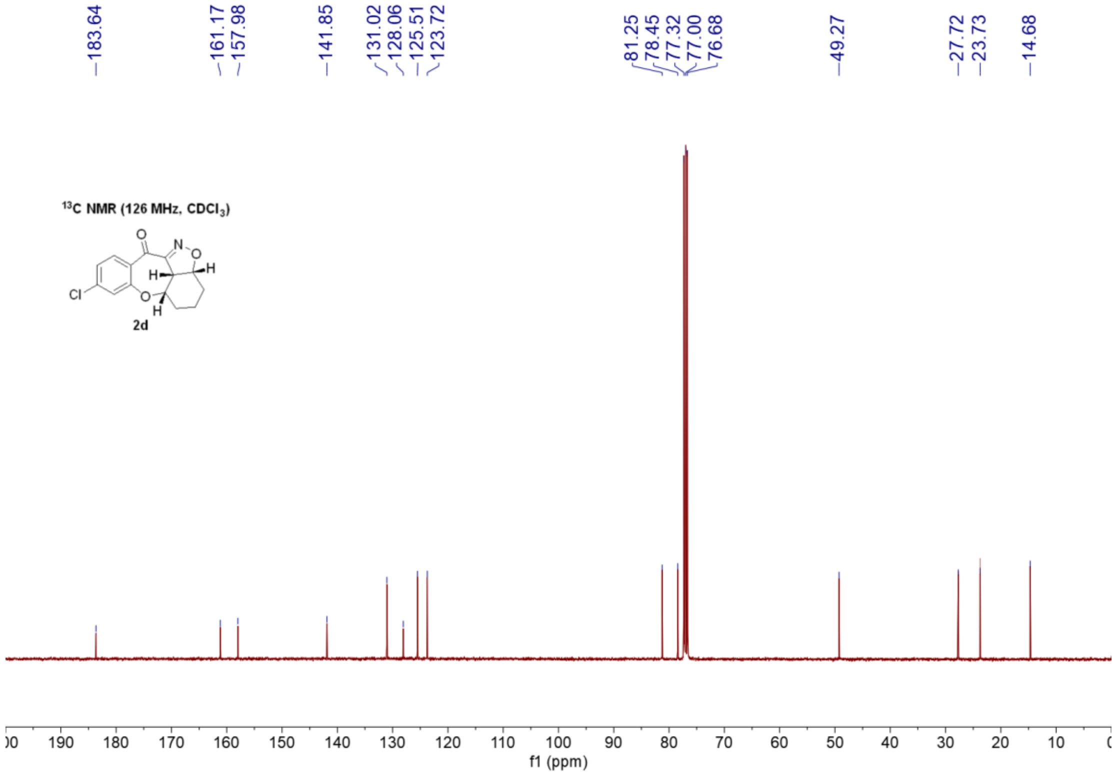

13C NMR (126 MHz, CDCl3)
2d
183.64
161.17
157.98
141.85
131.02
128.06
125.51
123.72
81.25
78.45
77.32
77.00
76.68
49.27
27.72
23.73
14.68
f1 (ppm)

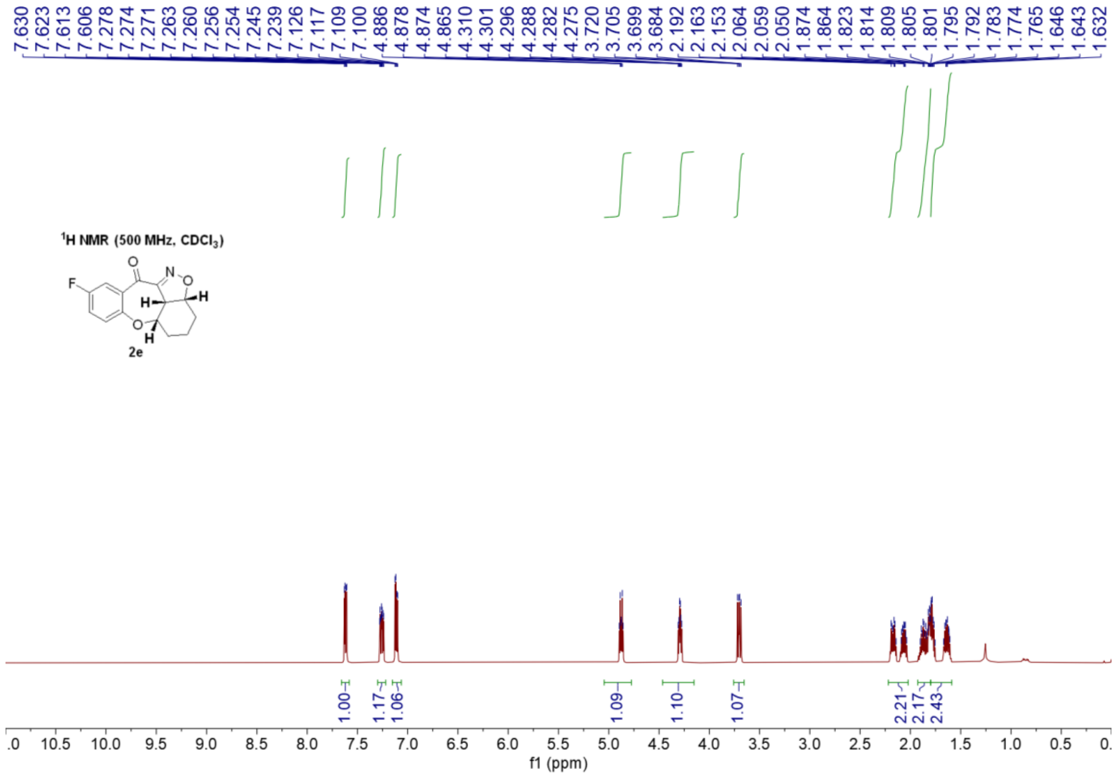
7.630 7.623 7.613 7.606 7.278 7.274 7.271 7.263 7.260 7.256 7.254 7.245 7.239 7.126 7.117 7.109 7.100 4.886 4.878 4.874 4.865 4.310 4.301 4.296 4.288 4.282 4.275 3.720 3.705 3.699 3.684 2.192 2.163 2.153 2.064 2.059 2.050 1.874 1.864 1.823 1.814 1.809 1.805 1.801 1.795 1.792 1.783 1.774 1.765 1.646 1.643 1.632
1H NMR (500 MHz, CDCl3)
2e
1.00 1.17 1.06 1.09 1.10 1.07 2.21 2.17 2.43
f1 (ppm)

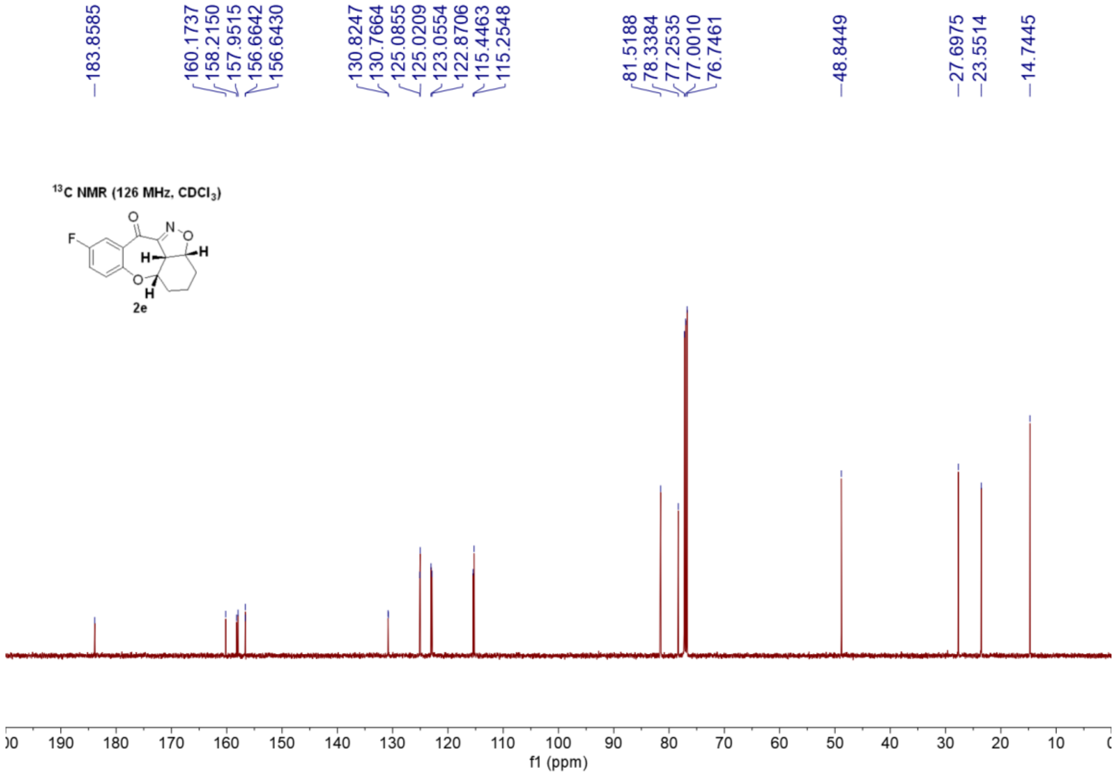
183.8585 160.1737 158.2150 157.9515 156.6642 156.6430 130.8247 130.7664 125.0855 125.0209 123.0554 122.8706 115.4463 115.2548 81.5188 78.3384 77.2535 77.0010 76.7461 48.8449 27.6975 23.5514 14.7445
13C NMR (126 MHz, CDCl3)
2e
f1 (ppm)

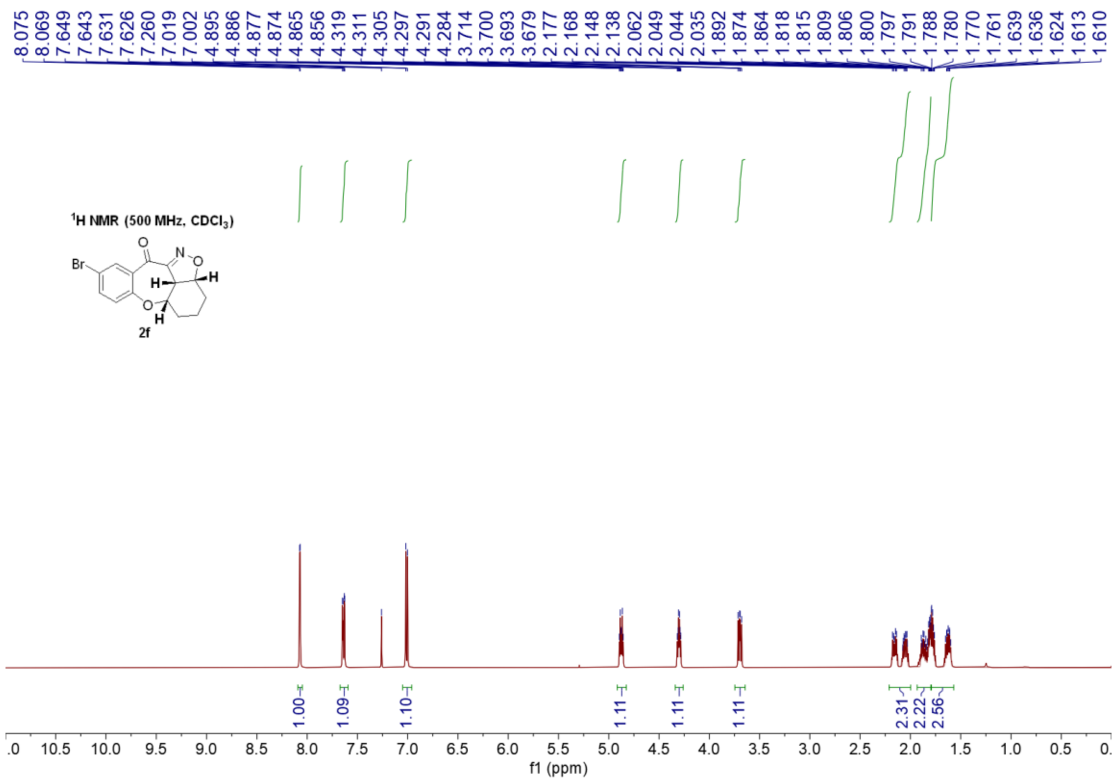

8.075
8.069
7.649
7.643
7.631
7.626
7.260
7.019
7.002
4.895
4.886
4.877
4.874
4.865
4.856
4.319
4.311
4.305
4.297
4.291
4.284
3.714
3.700
3.693
3.679
2.177
2.168
2.148
2.138
2.062
2.049
2.044
2.035
1.892
1.874
1.864
1.818
1.815
1.809
1.806
1.800
1.797
1.791
1.788
1.780
1.770
1.761
1.639
1.636
1.624
1.613
1.610
1H NMR (500 MHz, CDCl3)
Br
O
N
O
H
H
O
H
2f
1.00
1.09
1.10
1.11
1.11
1.11
2.31
2.22
2.56
f1 (ppm)

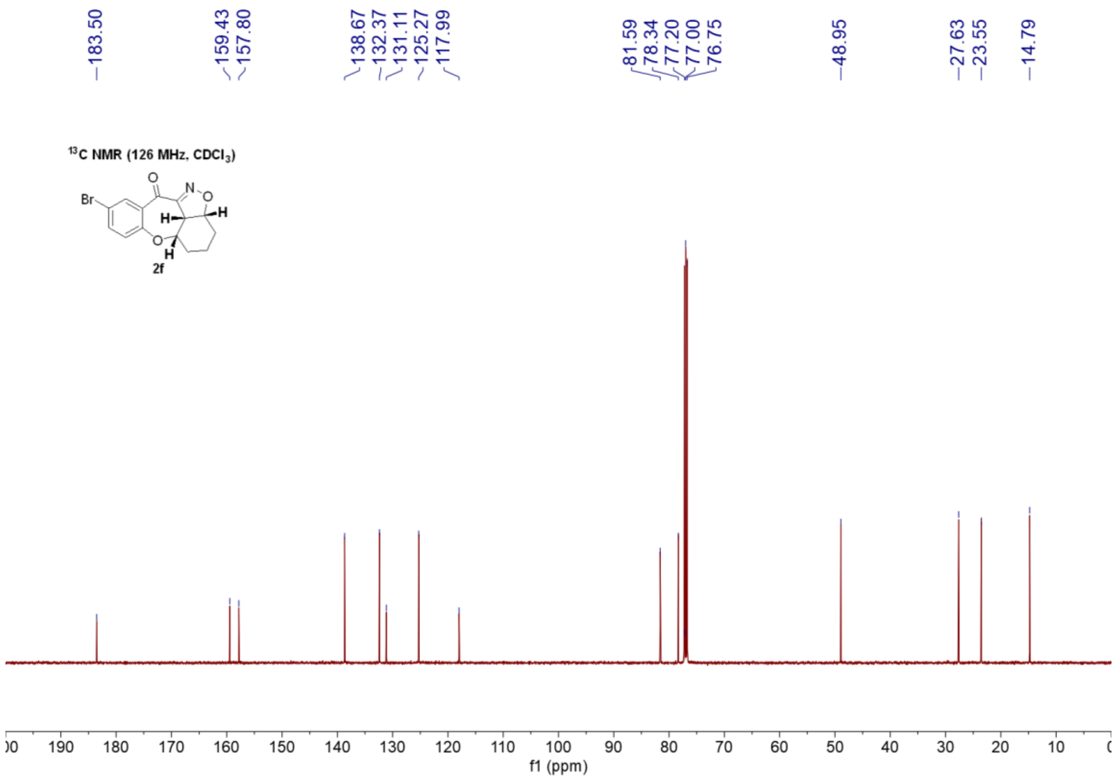

183.50
159.43
157.80
138.67
132.37
131.11
125.27
117.99
81.59
78.34
77.20
77.00
76.75
48.95
27.63
23.55
14.79
13C NMR (126 MHz, CDCl3)
Br
O
N
O
H
H
O
H
2f
f1 (ppm)

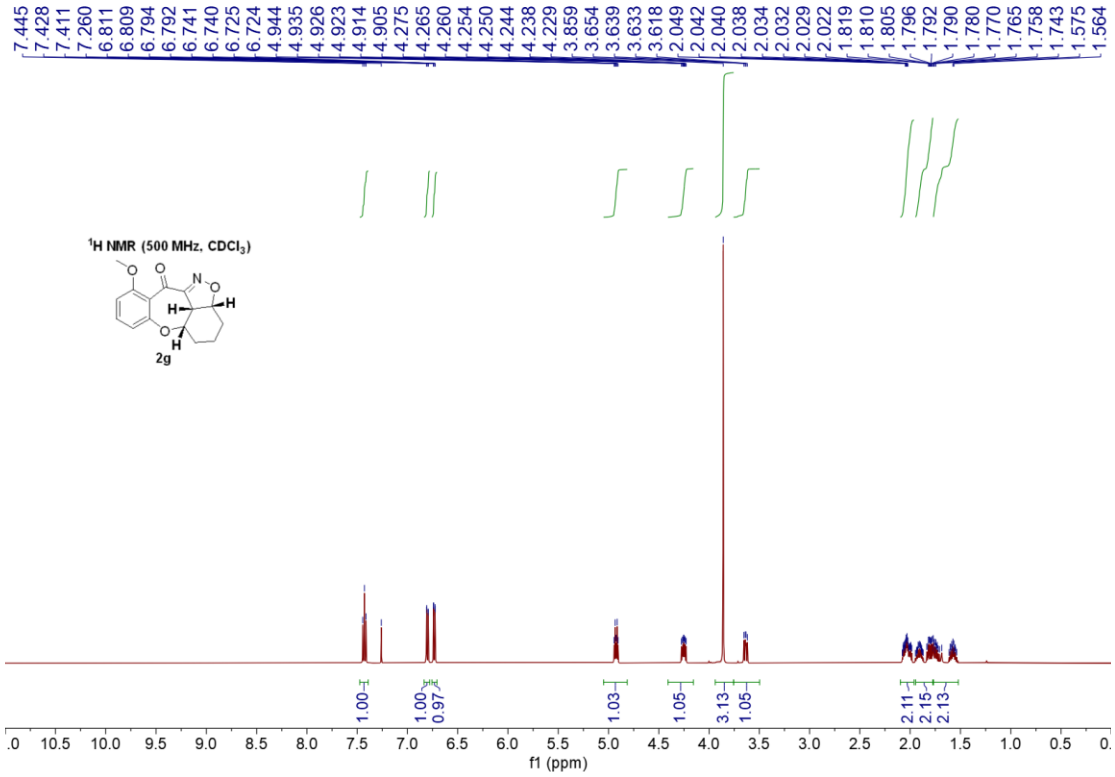

7.445
7.428
7.411
7.260
6.811
6.809
6.794
6.792
6.741
6.740
6.725
6.724
4.944
4.935
4.926
4.923
4.914
4.905
4.275
4.265
4.260
4.254
4.250
4.244
4.238
4.229
3.859
3.654
3.639
3.633
3.618
2.049
2.042
2.040
2.038
2.034
2.032
2.029
2.022
1.819
1.810
1.805
1.796
1.792
1.790
1.780
1.770
1.765
1.758
1.743
1.575
1.564
¹H NMR (500 MHz, CDCl₃)
2g
1.00
1.00
0.97
1.03
1.05
3.13
1.05
2.11
2.15
2.13
f1 (ppm)

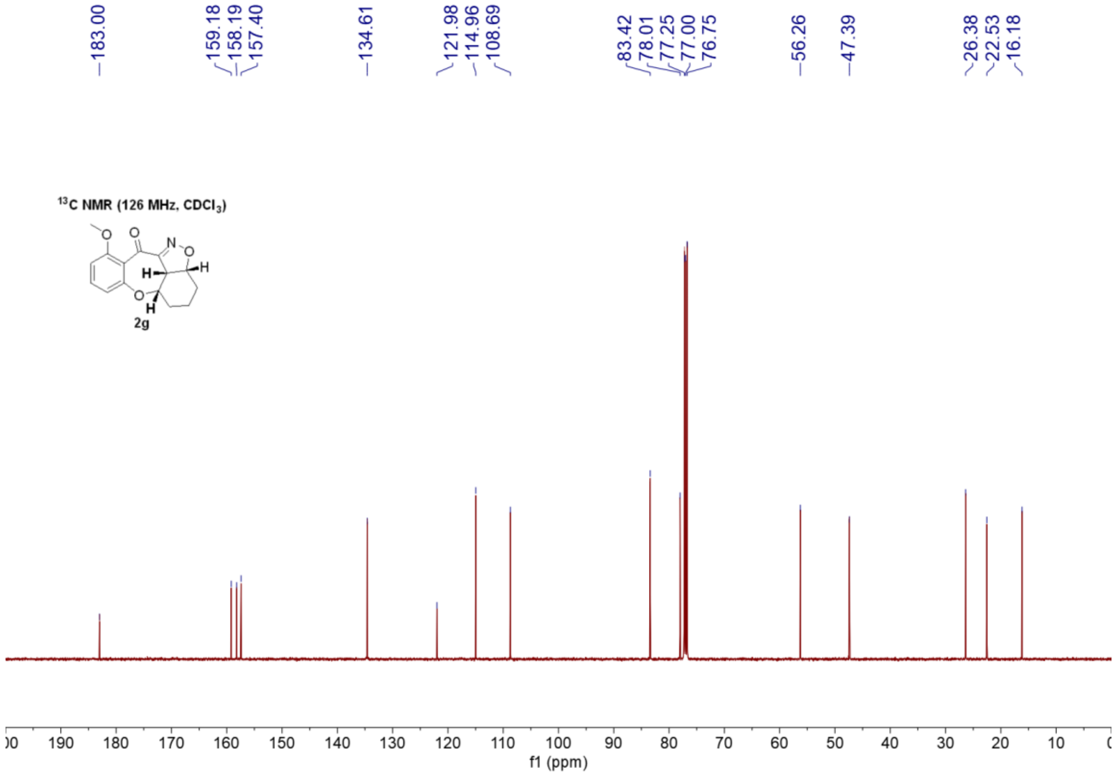

183.00
159.18
158.19
157.40
134.61
121.98
114.96
108.69
83.42
78.01
77.25
77.00
76.75
56.26
47.39
26.38
22.53
16.18
¹³C NMR (126 MHz, CDCl₃)
2g
f1 (ppm)

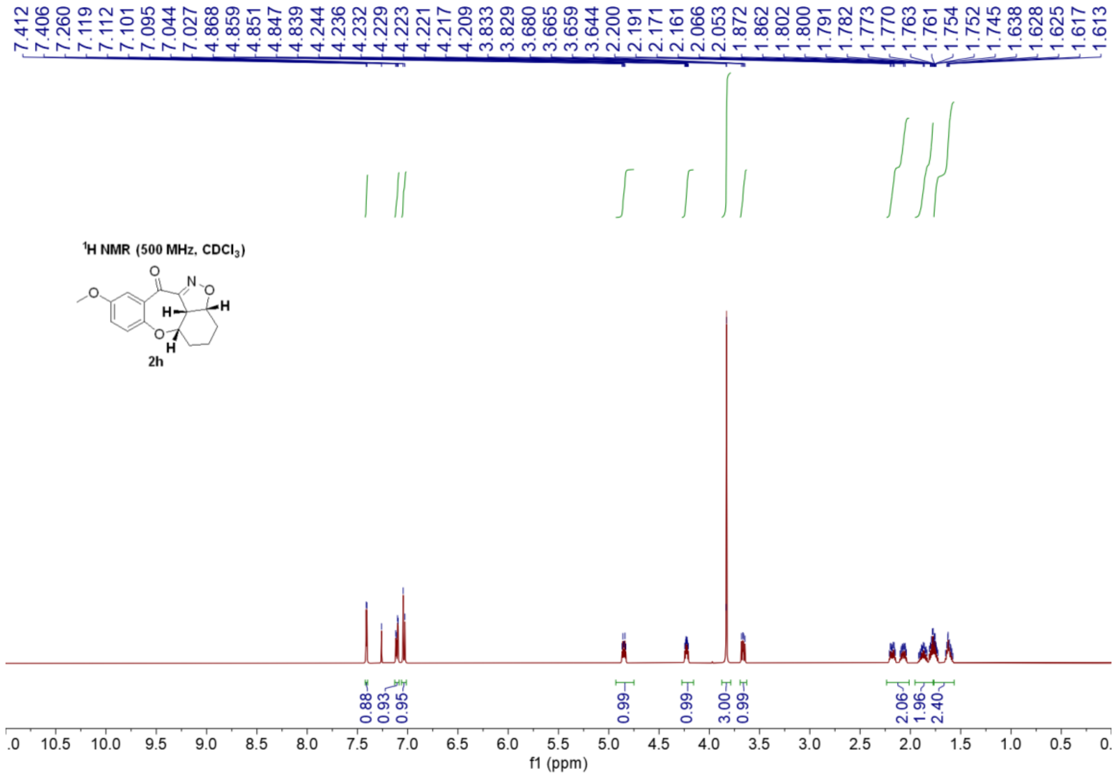
1H NMR (500 MHz, CDCl3)
2h
7.412
7.406
7.260
7.119
7.112
7.101
7.095
7.044
7.027
4.868
4.859
4.851
4.847
4.839
4.244
4.236
4.232
4.229
4.223
4.221
4.217
4.209
3.833
3.829
3.680
3.665
3.659
3.644
2.200
2.191
2.171
2.161
2.066
2.053
1.872
1.862
1.802
1.800
1.791
1.782
1.773
1.770
1.763
1.761
1.754
1.752
1.745
1.638
1.628
1.625
1.617
1.613
0.88
0.93
0.95
0.99
0.99
3.00
0.99
2.06
1.96
2.40
f1 (ppm)

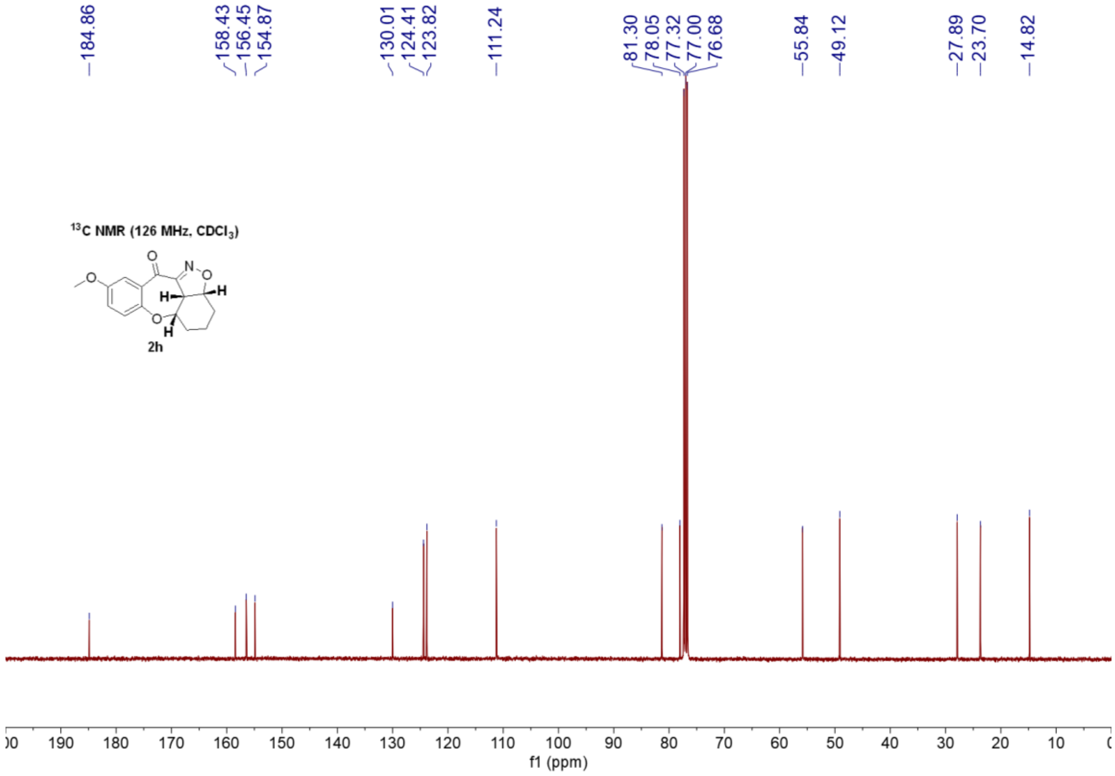
13C NMR (126 MHz, CDCl3)
2h
184.86
158.43
156.45
154.87
130.01
124.41
123.82
111.24
81.30
78.05
77.32
77.00
76.68
55.84
49.12
27.89
23.70
14.82
f1 (ppm)

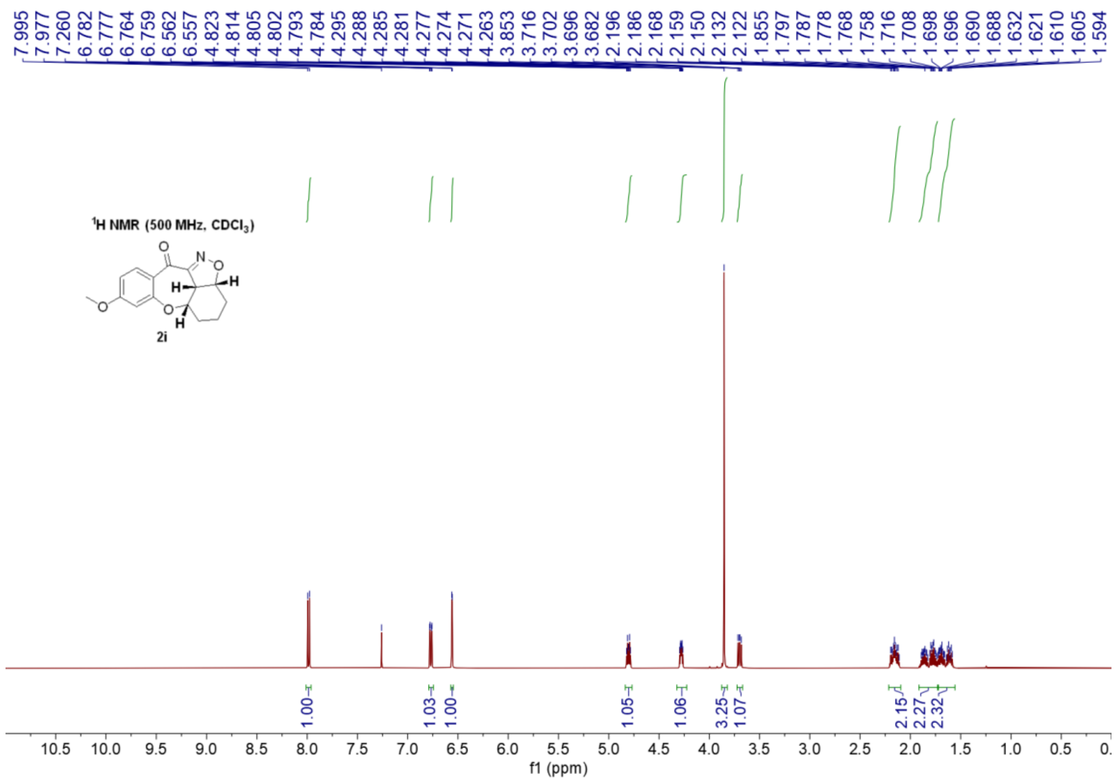

1H NMR (500 MHz, CDCl3)
2i
f1 (ppm)

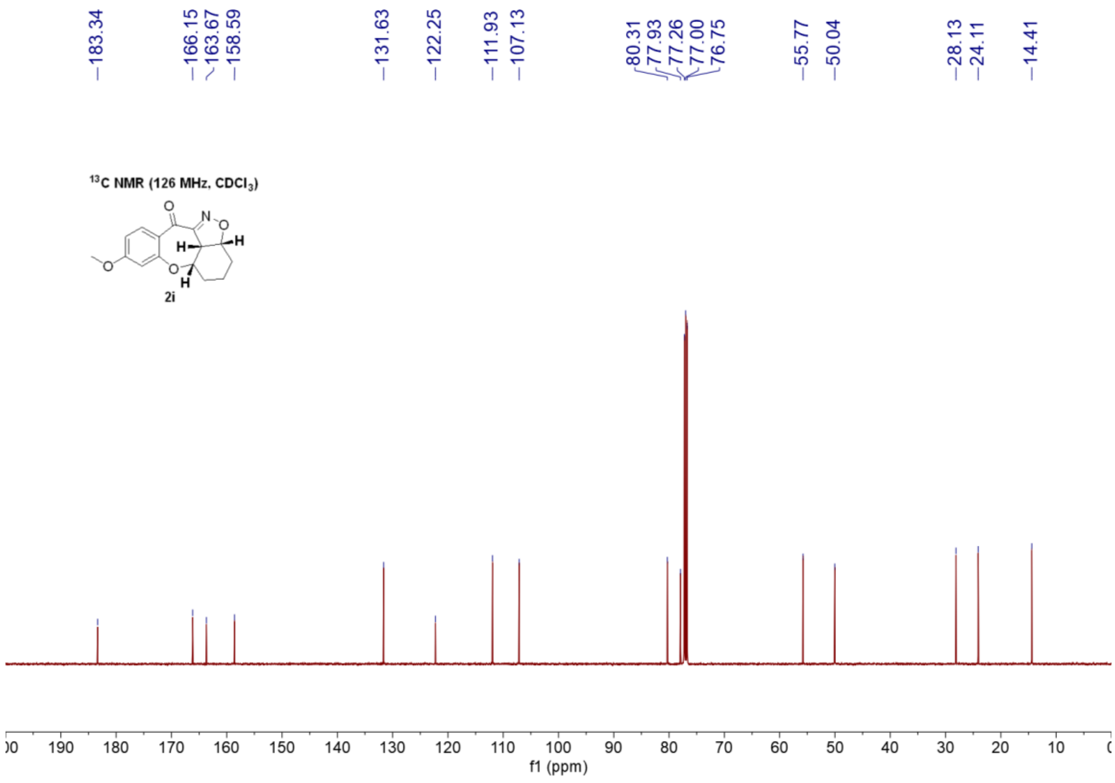

13C NMR (126 MHz, CDCl3)
2i
f1 (ppm)

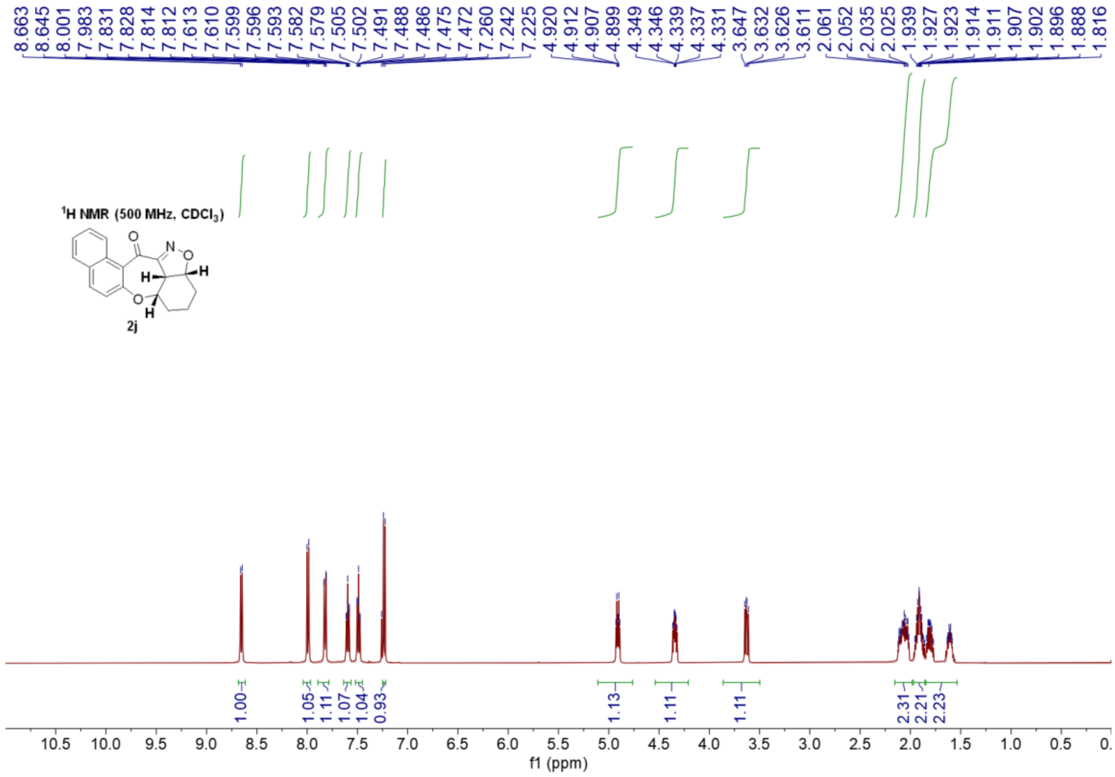
1H NMR (500 MHz, CDCl3)
2j
f1 (ppm)

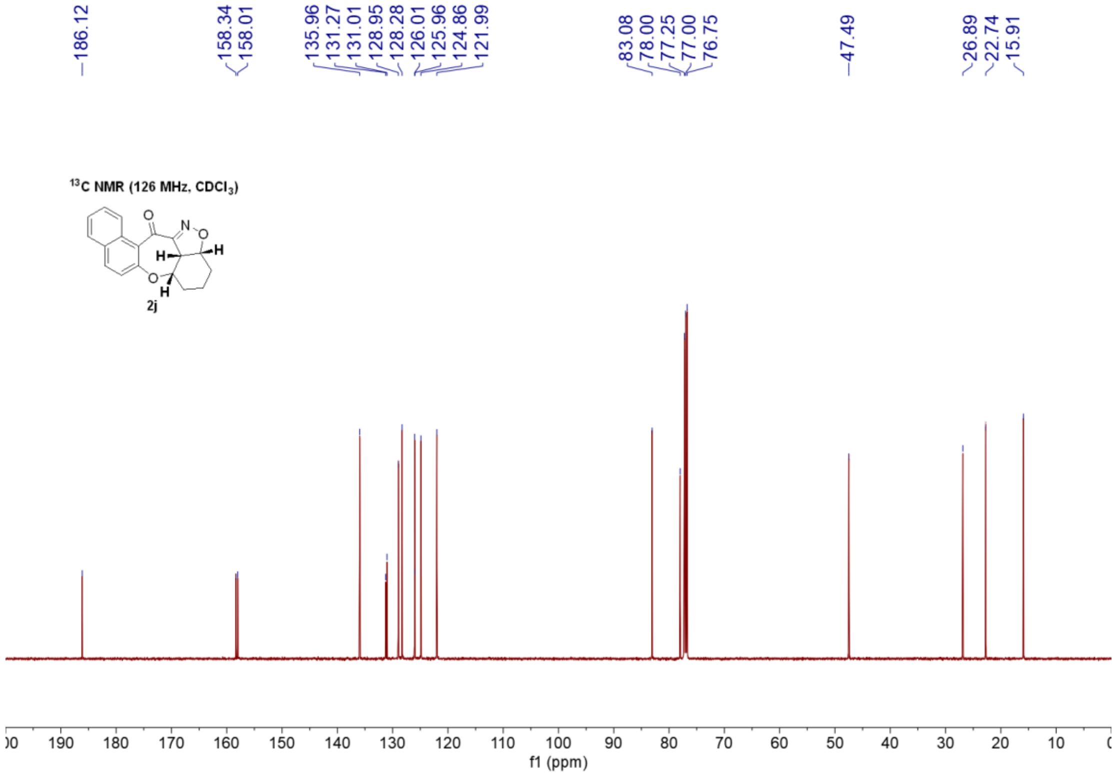
13C NMR (126 MHz, CDCl3)
2j
f1 (ppm)

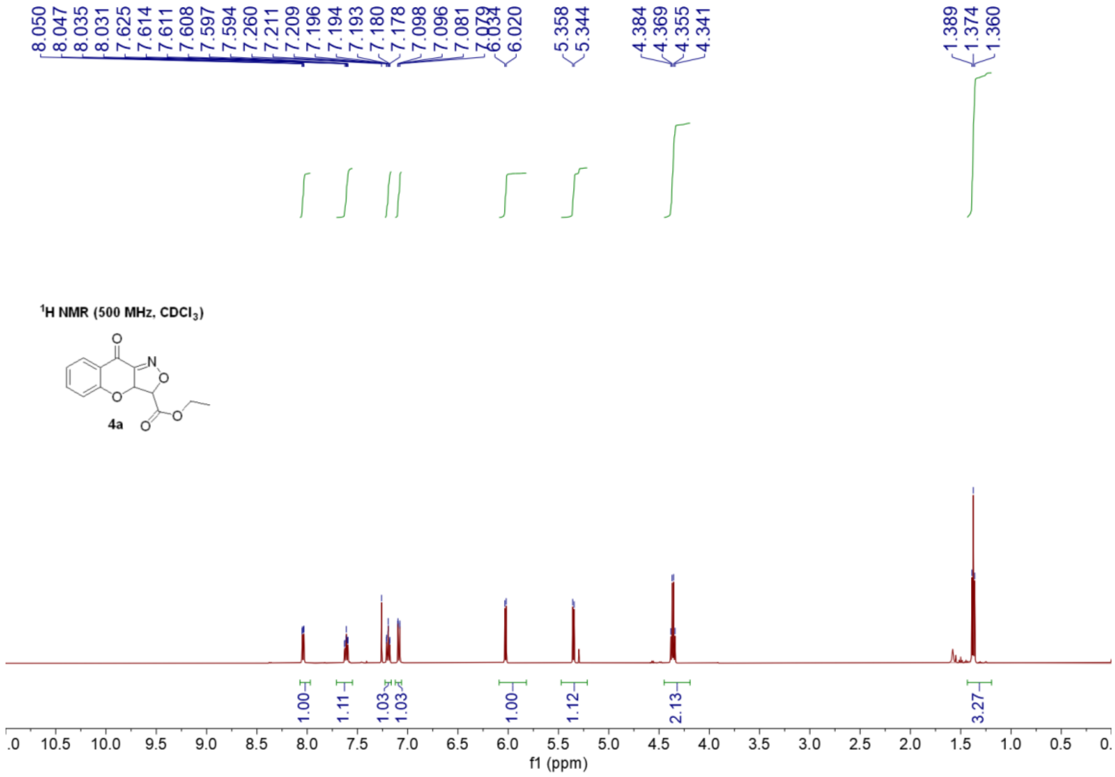
1H NMR (500 MHz, CDCl3)
4a
f1 (ppm)

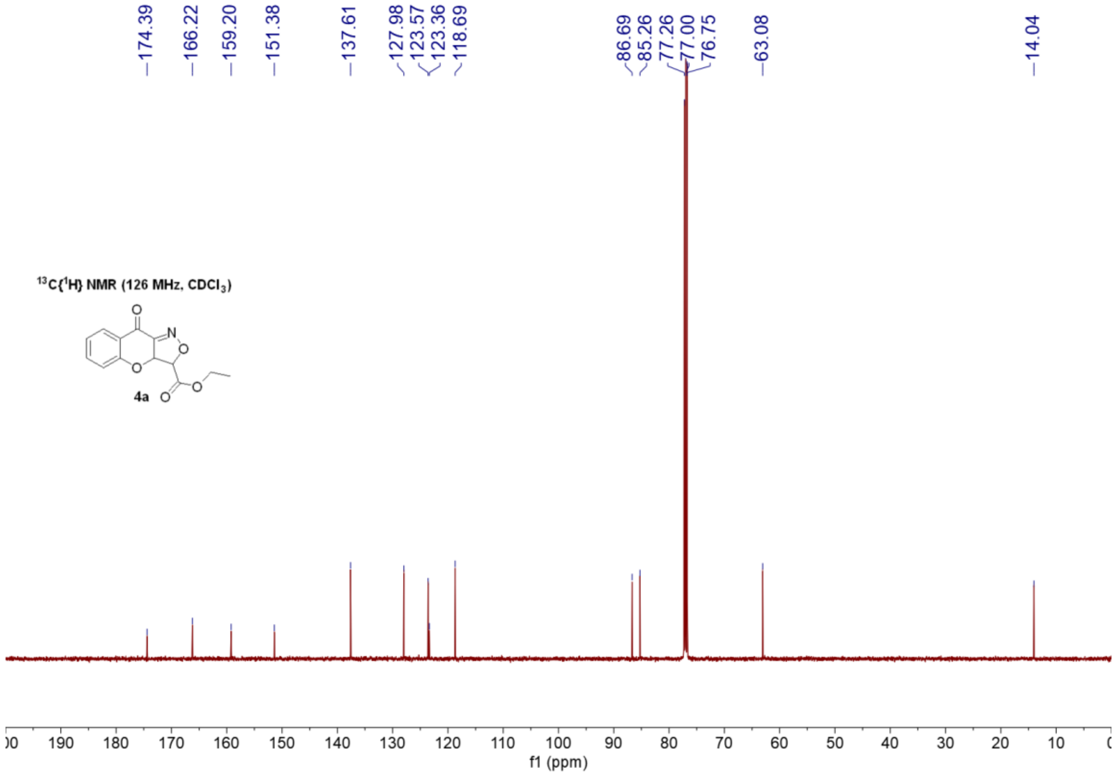
13C{1H} NMR (126 MHz, CDCl3)
4a
f1 (ppm)

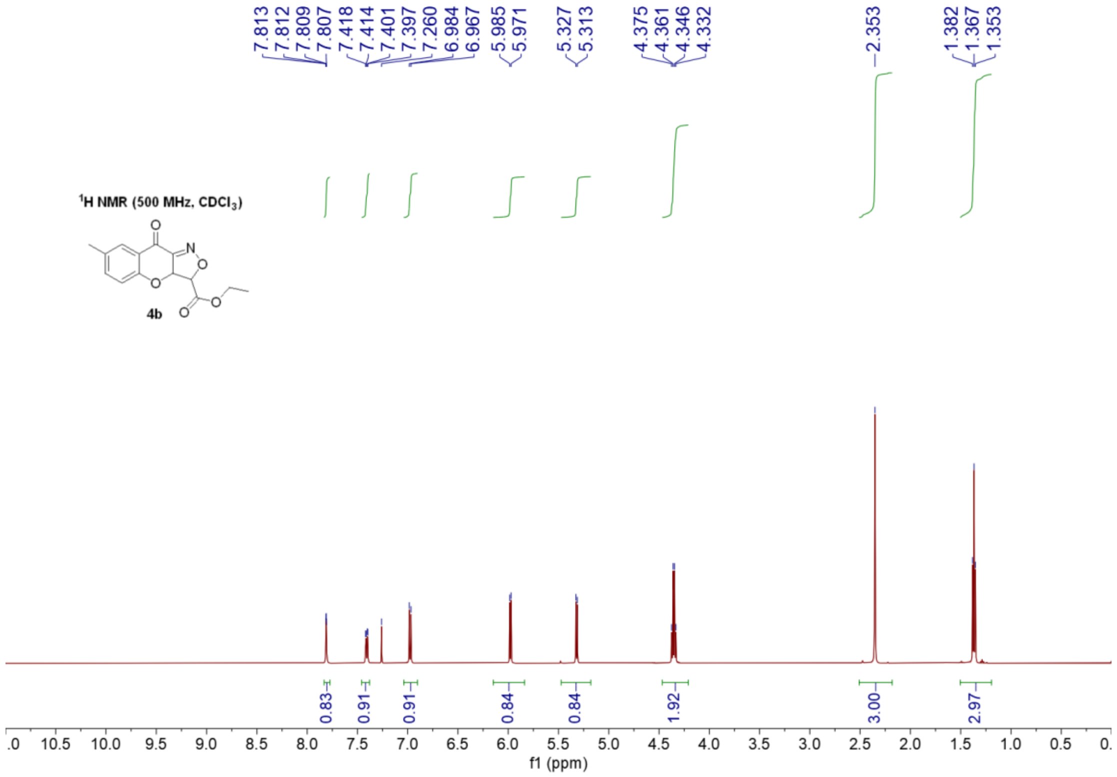

^{1}H NMR (500 MHz, CDCl$_3$)
4b
7.813
7.812
7.809
7.807
7.418
7.414
7.401
7.397
7.260
6.984
6.967
5.985
5.971
5.327
5.313
4.375
4.361
4.346
4.332
2.353
1.382
1.367
1.353
0.83
0.91
0.91
0.84
0.84
1.92
3.00
2.97
f1 (ppm)

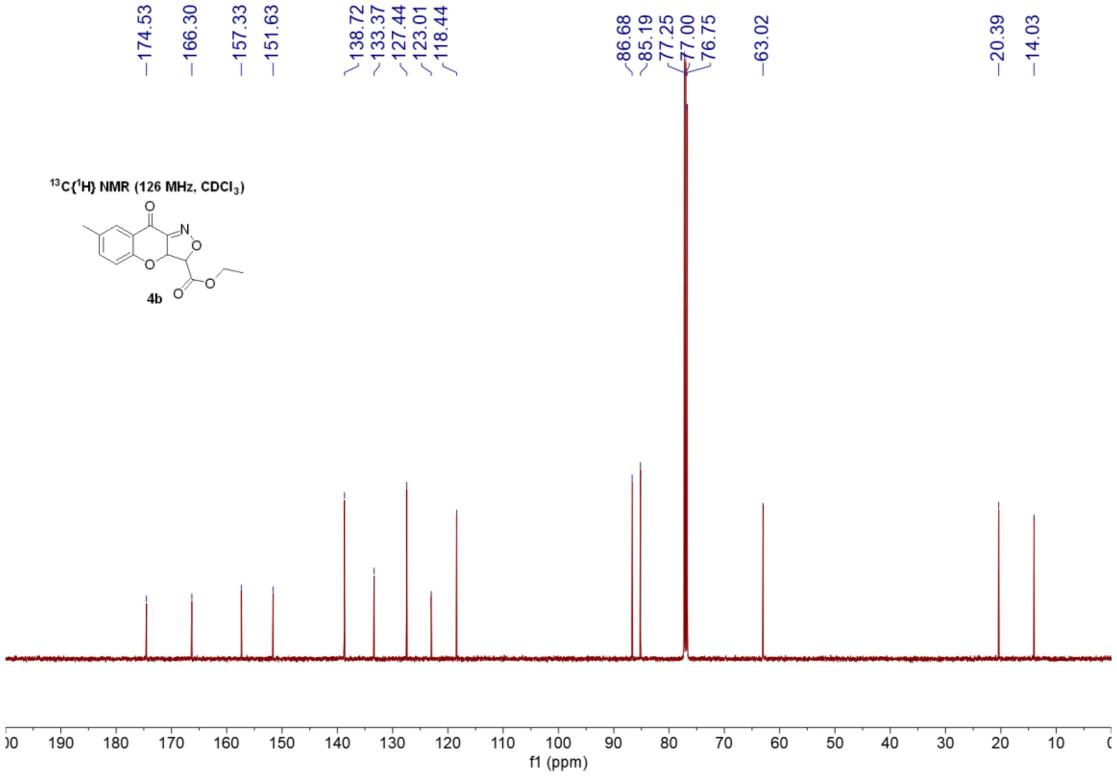

^{13}C{^{1}H} NMR (126 MHz, CDCl$_3$)
4b
174.53
166.30
157.33
151.63
138.72
133.37
127.44
123.01
118.44
86.68
85.19
77.25
77.00
76.75
63.02
20.39
14.03
f1 (ppm)

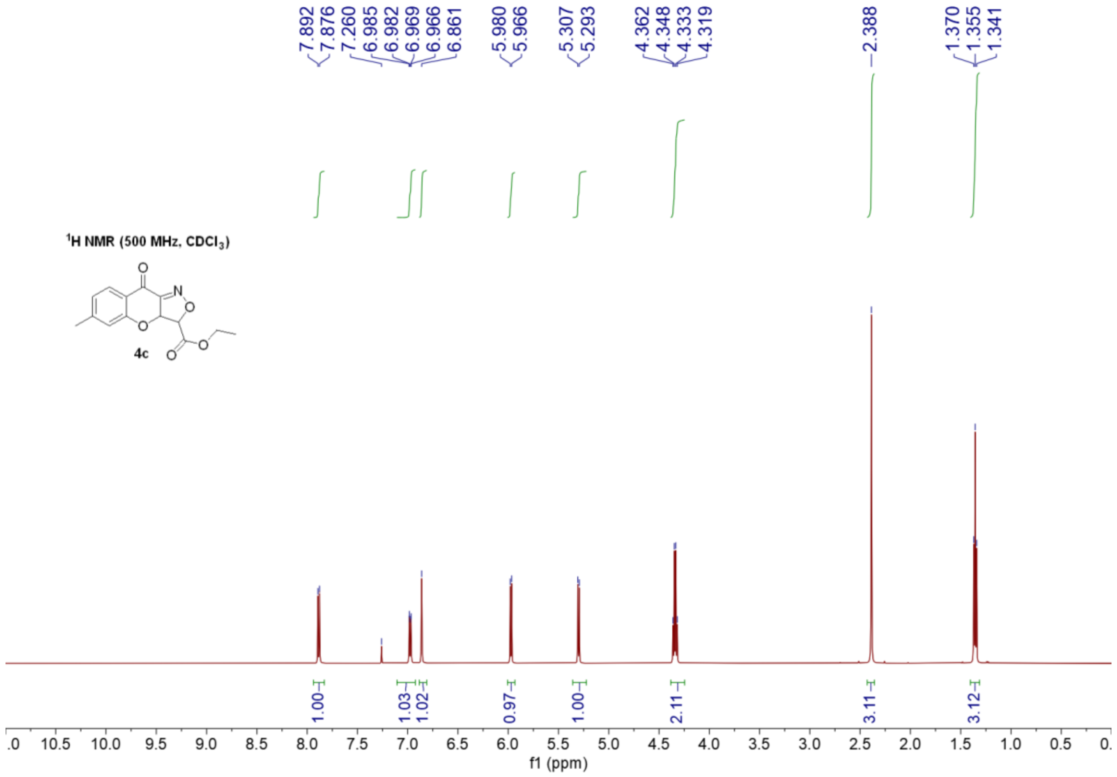

1H NMR (500 MHz, CDCl3)
4c
7.892 7.876 7.260 6.985 6.982 6.969 6.966 6.861 5.980 5.966 5.307 5.293 4.362 4.348 4.333 4.319 2.388 1.370 1.355 1.341
1.00 1.03 1.02 0.97 1.00 2.11 3.11 3.12
f1 (ppm)

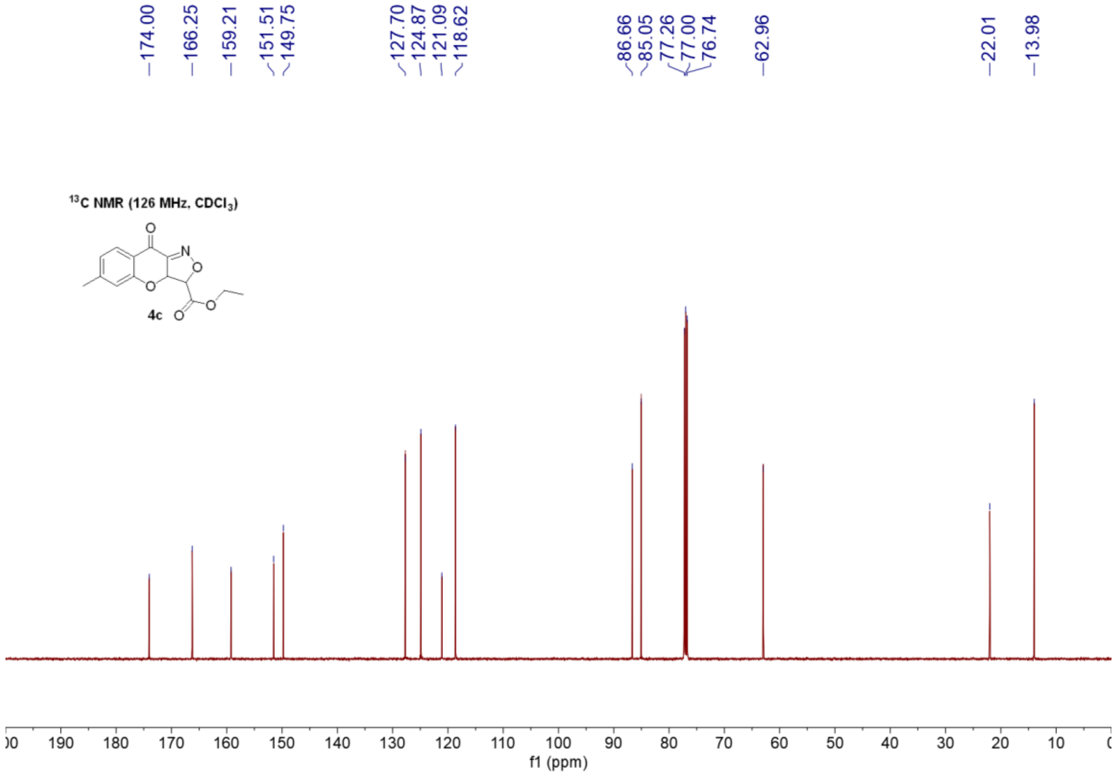

13C NMR (126 MHz, CDCl3)
4c
174.00 166.25 159.21 151.51 149.75 127.70 124.87 121.09 118.62 86.66 85.05 77.26 77.00 76.74 62.96 22.01 13.98
f1 (ppm)

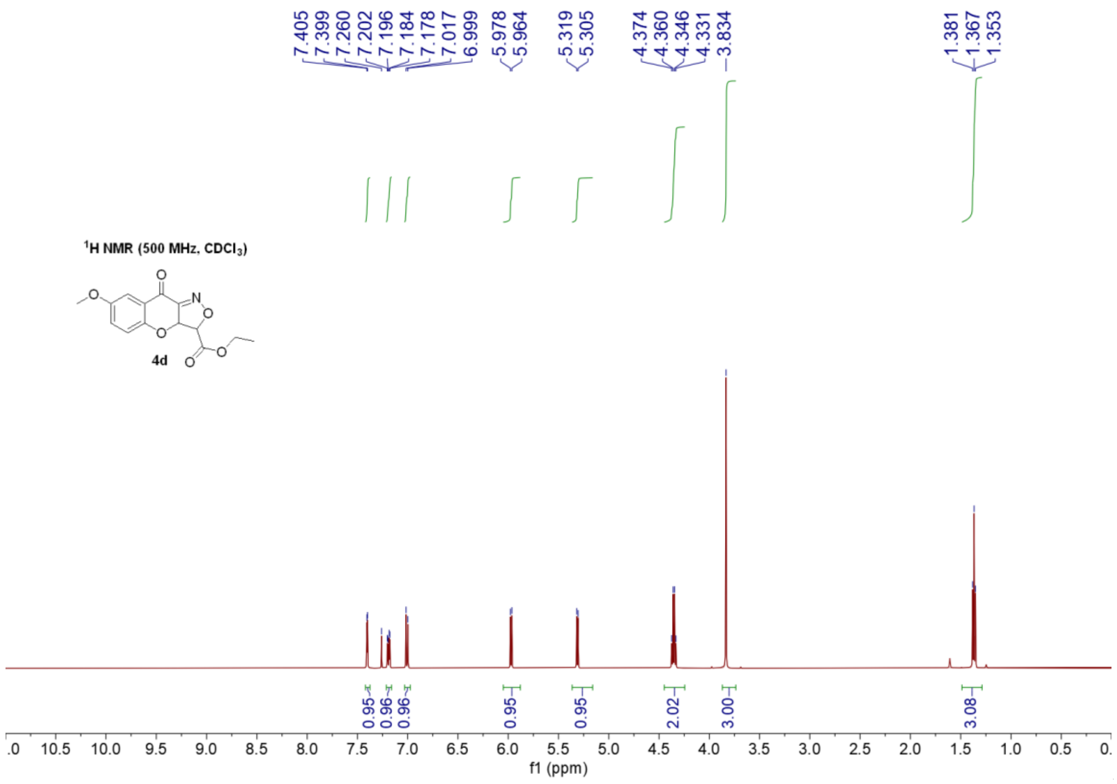
^{1}H NMR (500 MHz, CDCl$_3$)
4d
f1 (ppm)

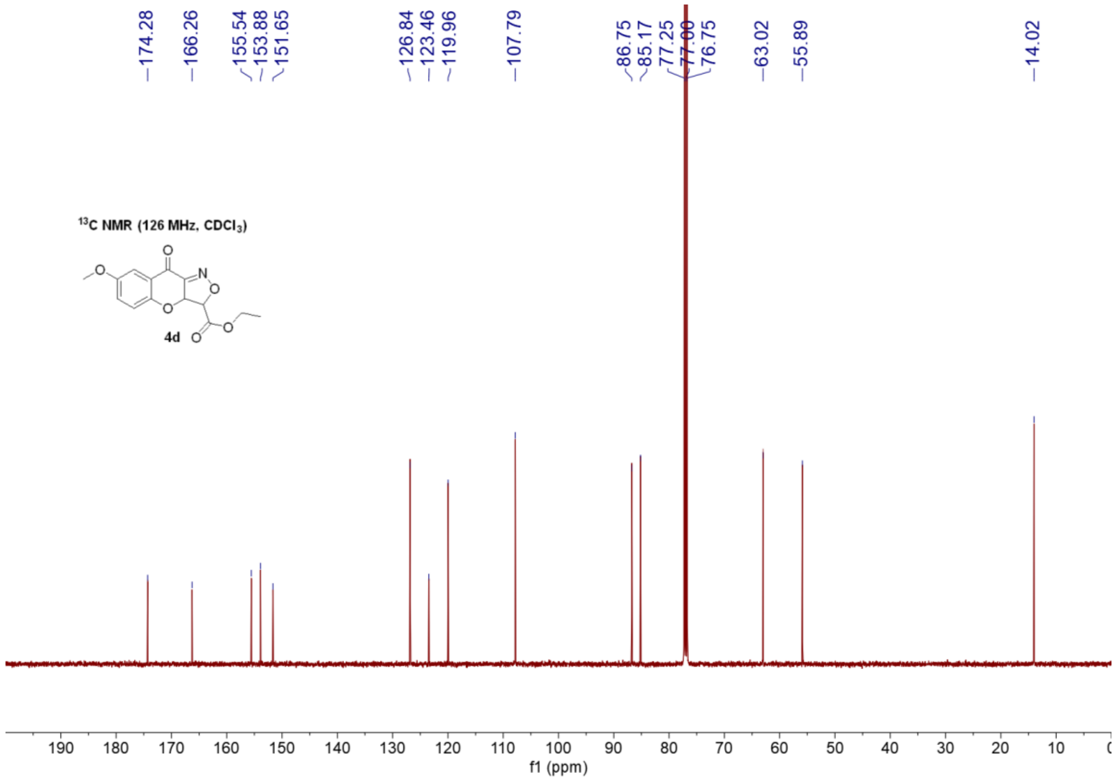
^{13}C NMR (126 MHz, CDCl$_3$)
4d
f1 (ppm)

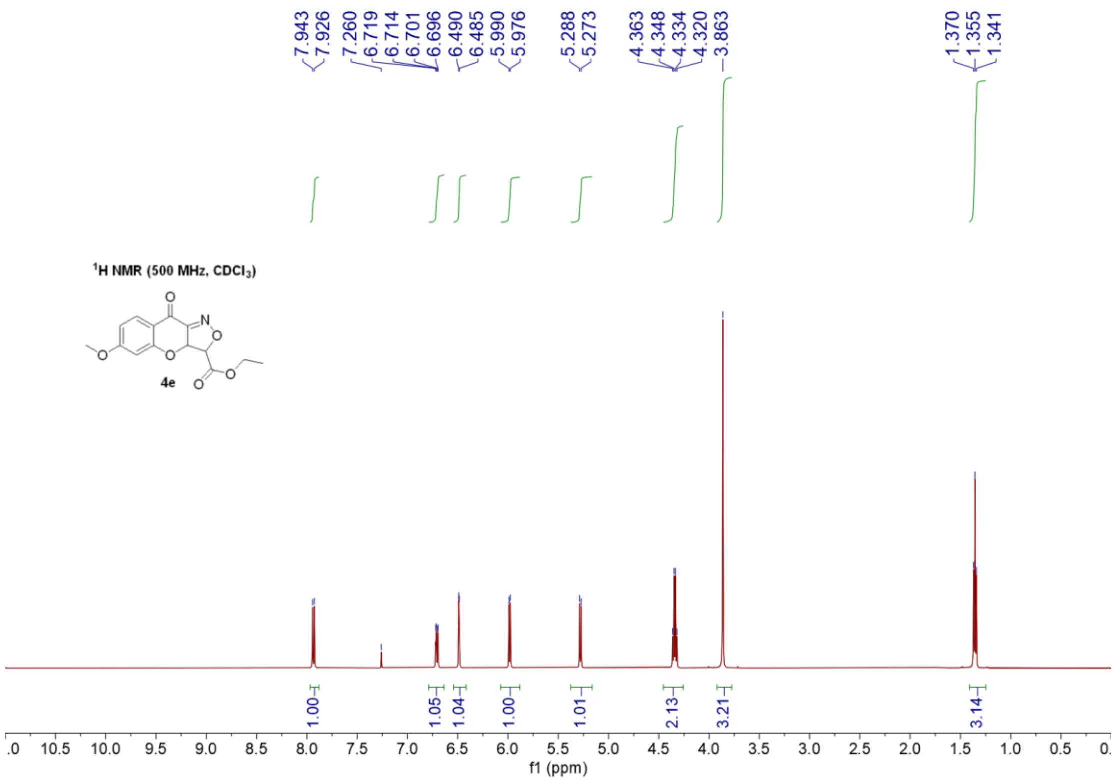

1H NMR (500 MHz, CDCl3)
4e
f1 (ppm)

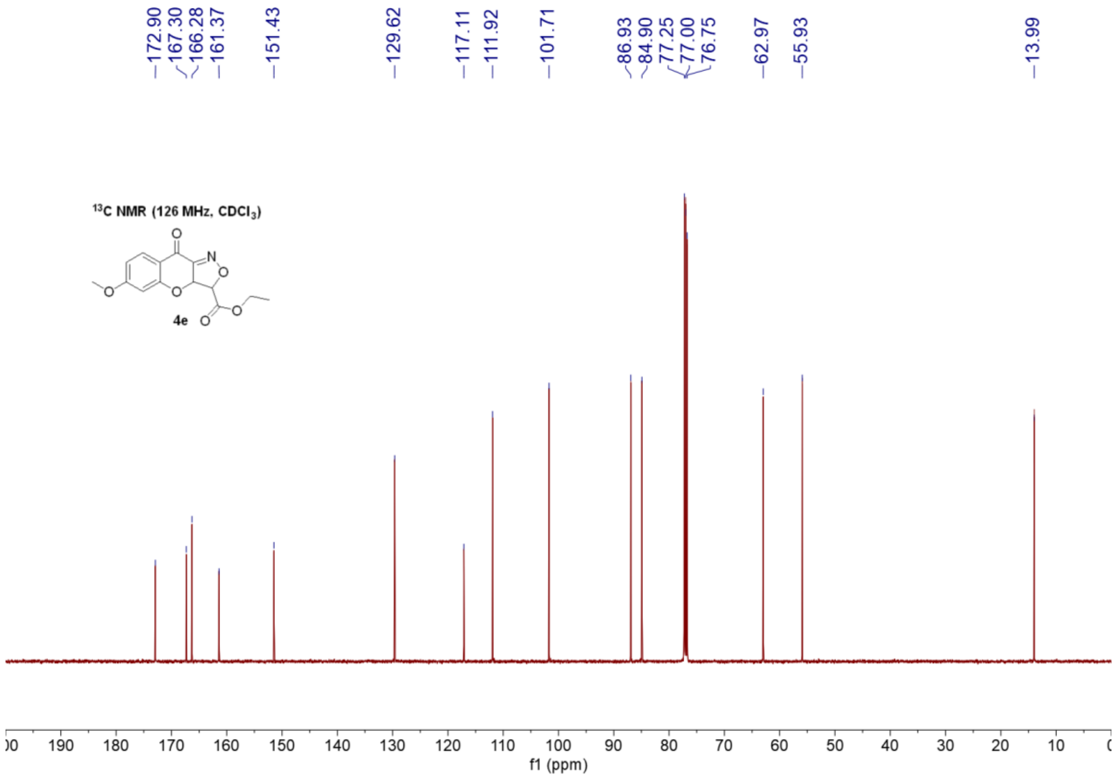

13C NMR (126 MHz, CDCl3)
4e
f1 (ppm)

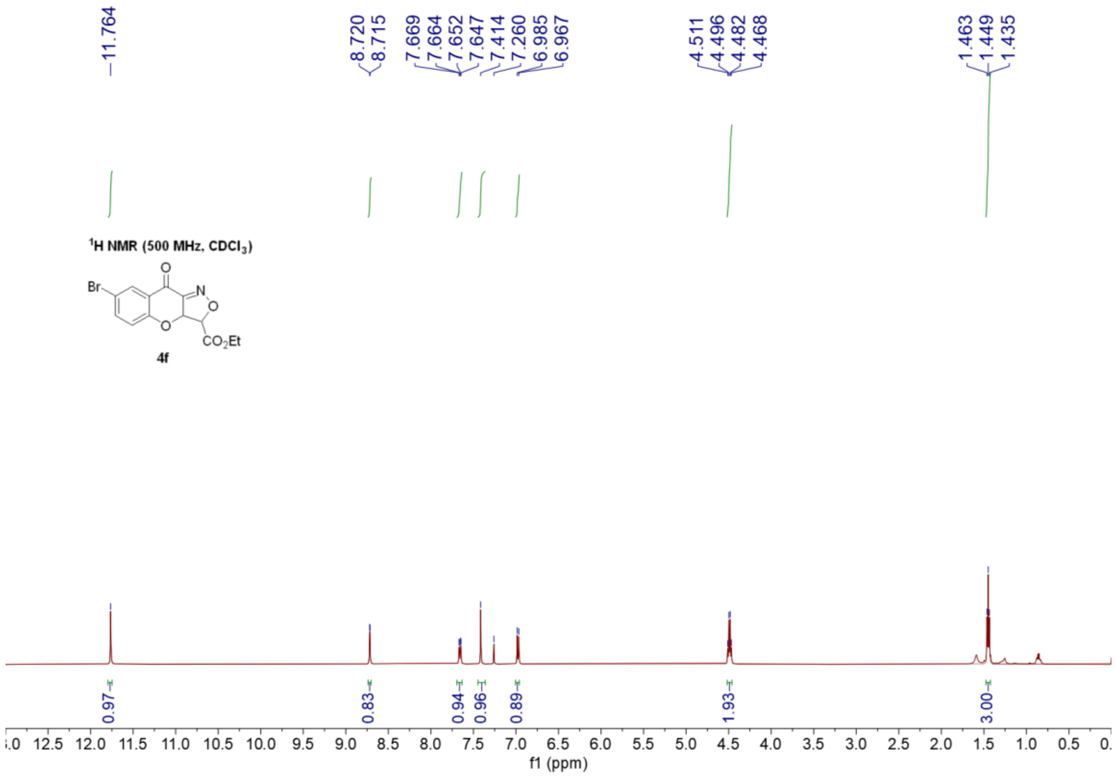
11.764
8.720
8.715
7.669
7.664
7.652
7.647
7.414
7.260
6.985
6.967
4.511
4.496
4.482
4.468
1.463
1.449
1.435
1H NMR (500 MHz, CDCl3)
Br
O
N
O
O
CO2Et
4f
0.97
0.83
0.94
0.96
0.89
1.93
3.00
f1 (ppm)

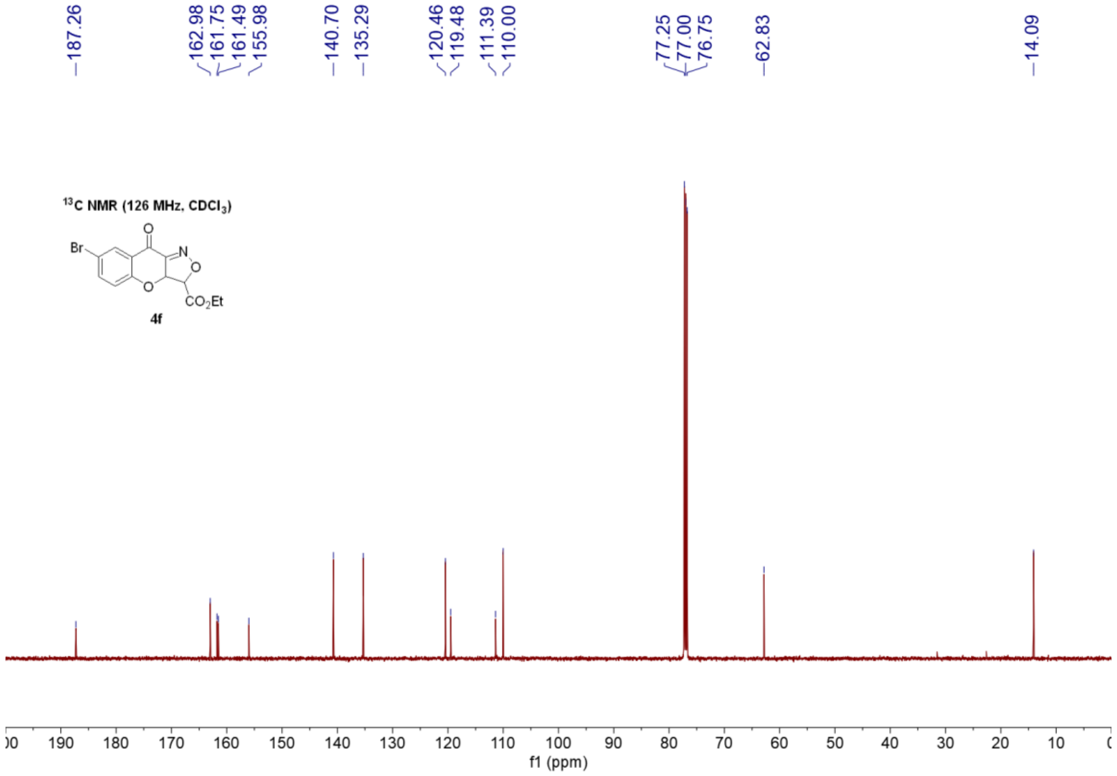
187.26
162.98
161.75
161.49
155.98
140.70
135.29
120.46
119.48
111.39
110.00
77.25
77.00
76.75
62.83
14.09
13C NMR (126 MHz, CDCl3)
Br
O
N
O
O
CO2Et
4f
f1 (ppm)

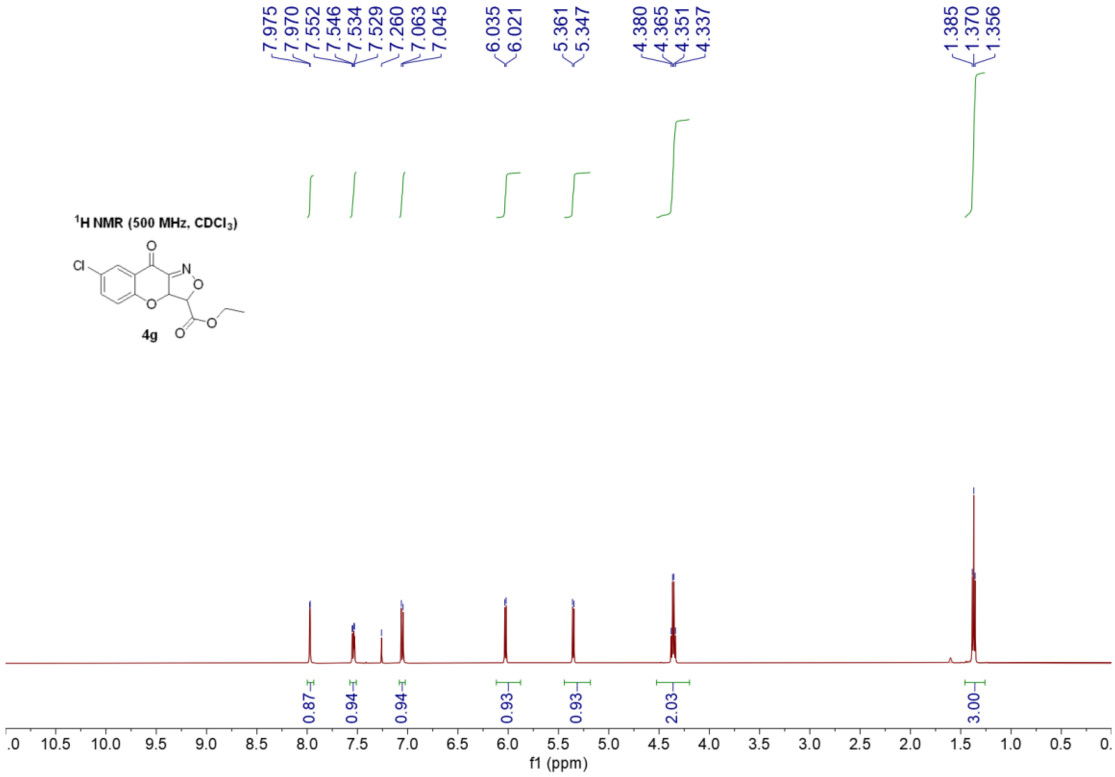
7.975
7.970
7.552
7.546
7.534
7.529
7.260
7.063
7.045
6.035
6.021
5.361
5.347
4.380
4.365
4.351
4.337
1.385
1.370
1.356
1H NMR (500 MHz, CDCl3)
Cl
O
N
O
O
O
O
4g
0.87
0.94
0.94
0.93
0.93
2.03
3.00
f1 (ppm)

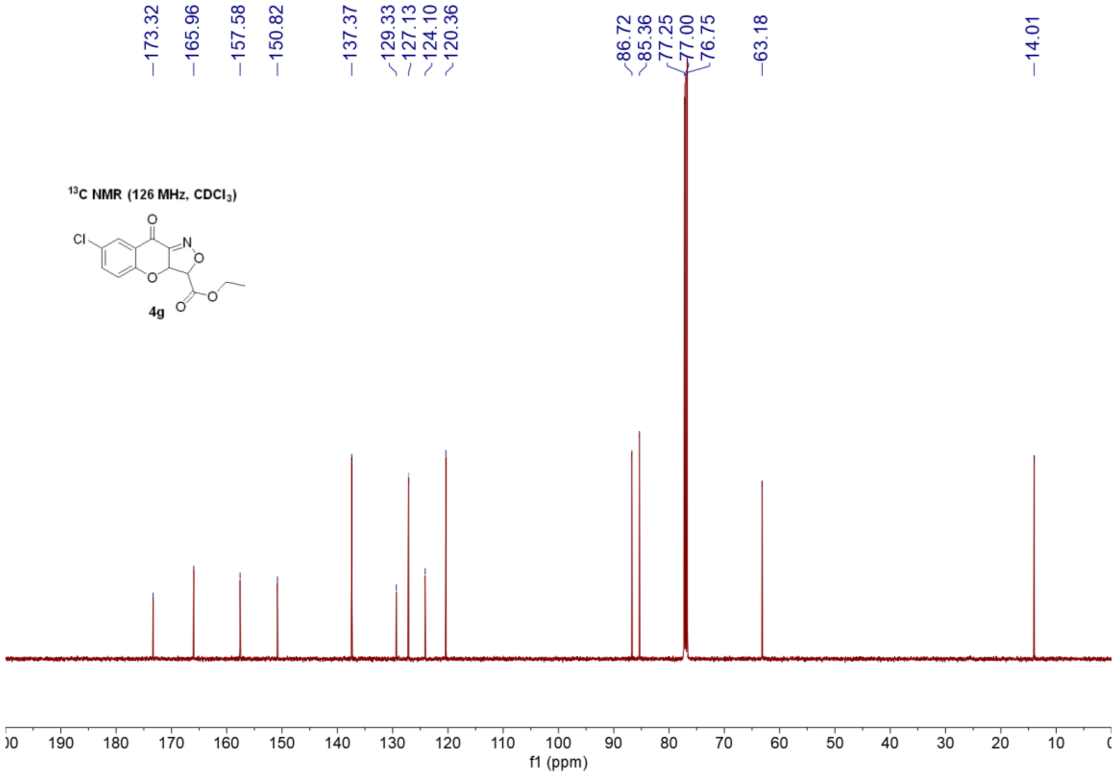
173.32
165.96
157.58
150.82
137.37
129.33
127.13
124.10
120.36
86.72
85.36
77.25
77.00
76.75
63.18
14.01
13C NMR (126 MHz, CDCl3)
Cl
O
N
O
O
O
O
4g
f1 (ppm)

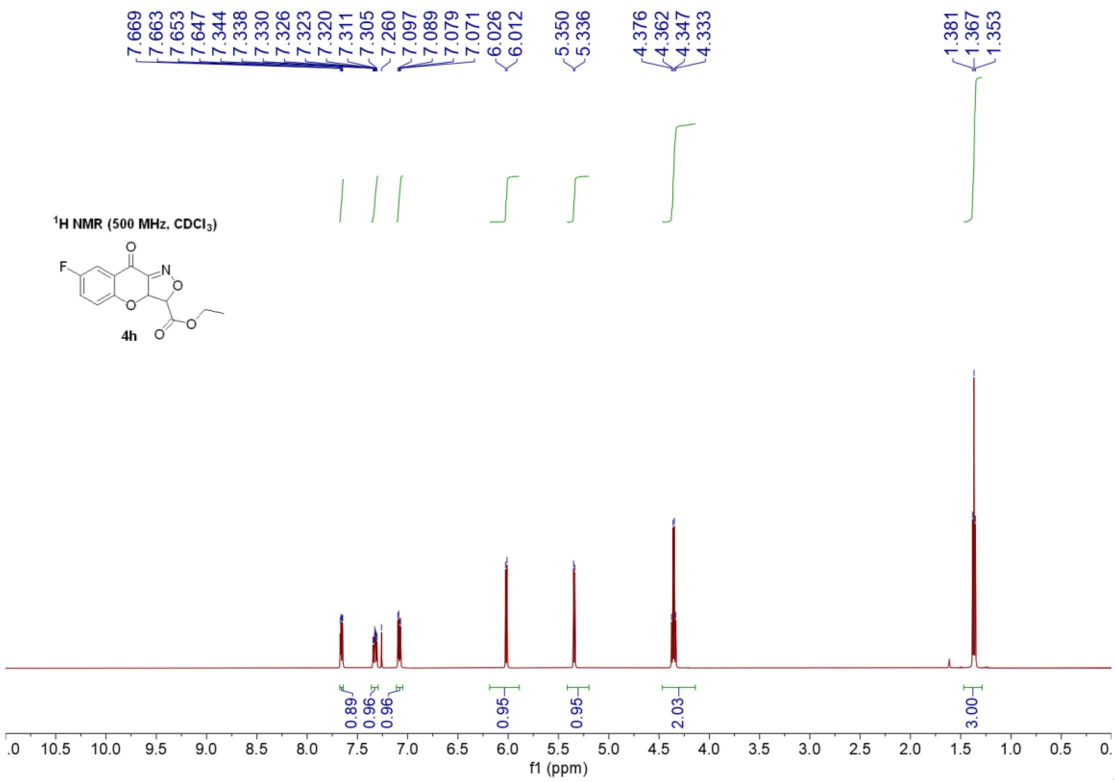

7.669
7.663
7.653
7.647
7.344
7.338
7.330
7.326
7.323
7.320
7.311
7.305
7.260
7.097
7.089
7.079
7.071
6.026
6.012
5.350
5.336
4.376
4.362
4.347
4.333
1.381
1.367
1.353
^{1}H NMR (500 MHz, CDCl$_3$)
4h
0.89
0.96
0.96
0.95
0.95
2.03
3.00
f1 (ppm)

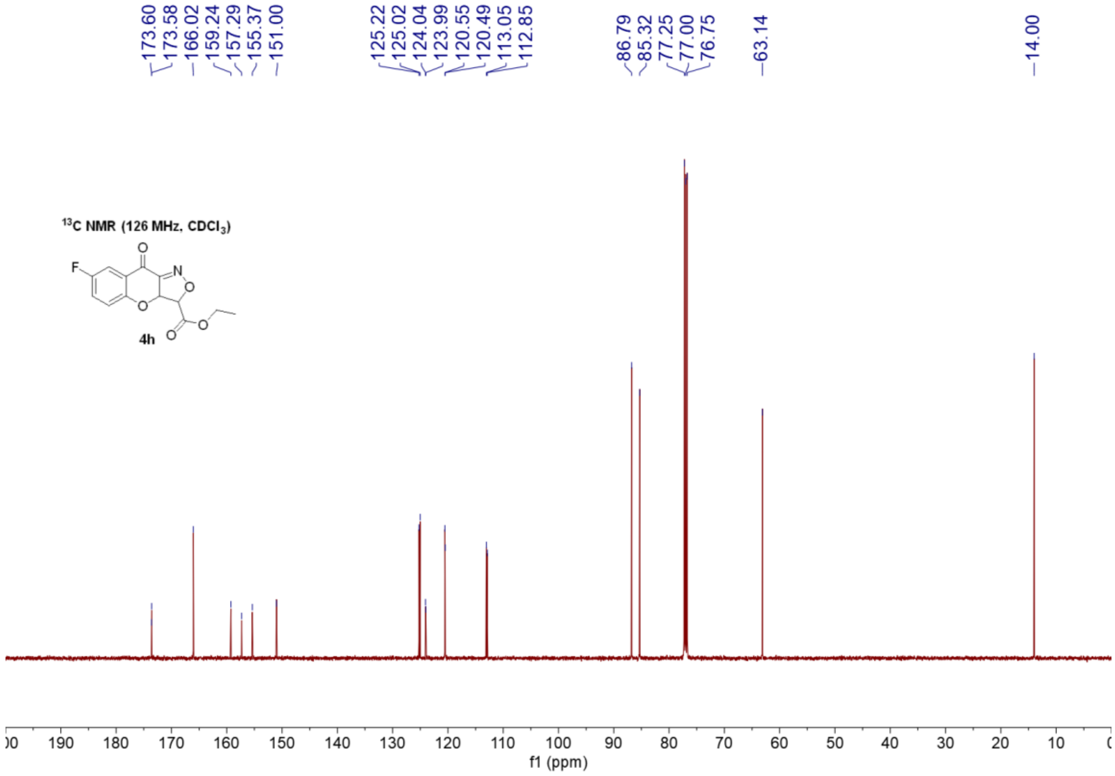

173.60
173.58
166.02
159.24
157.29
155.37
151.00
125.22
125.02
124.04
123.99
120.55
120.49
113.05
112.85
86.79
85.32
77.25
77.00
76.75
63.14
14.00
^{13}C NMR (126 MHz, CDCl$_3$)
4h
f1 (ppm)

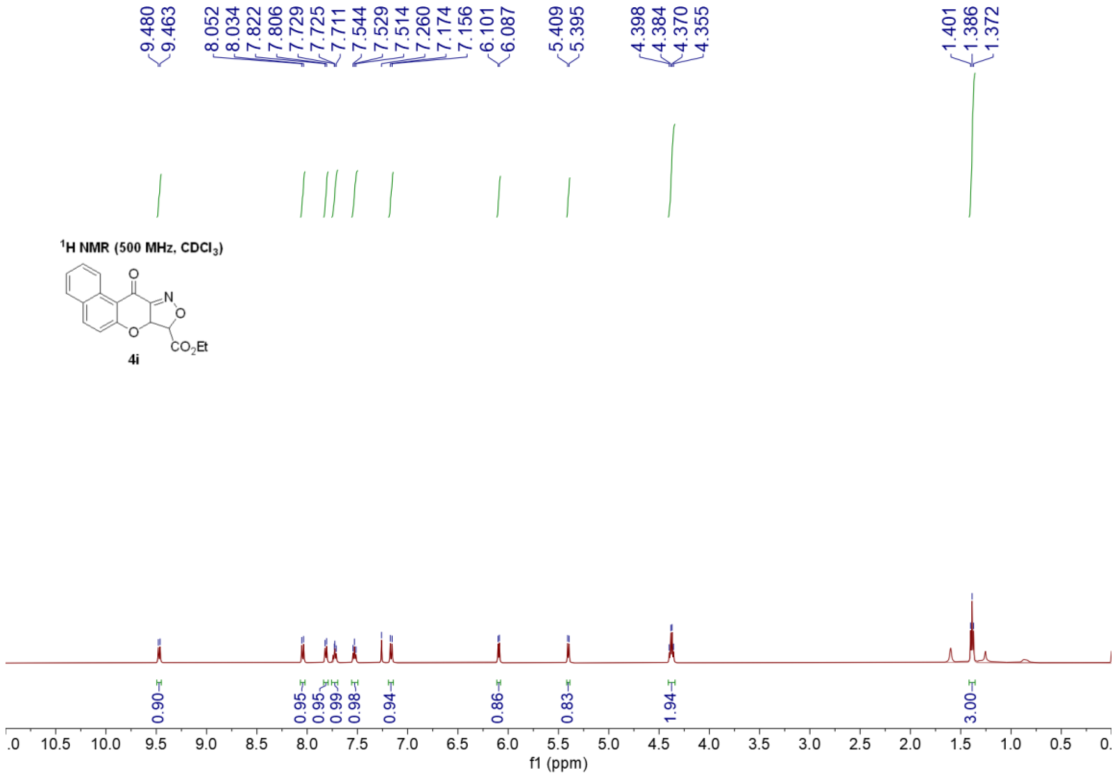

9.480
9.463
8.052
8.034
7.822
7.806
7.729
7.725
7.711
7.544
7.529
7.514
7.260
7.174
7.156
6.101
6.087
5.409
5.395
4.398
4.384
4.370
4.355
1.401
1.386
1.372
1H NMR (500 MHz, CDCl3)
4i
0.90
0.95
0.95
0.99
0.98
0.94
0.86
0.83
1.94
3.00
f1 (ppm)

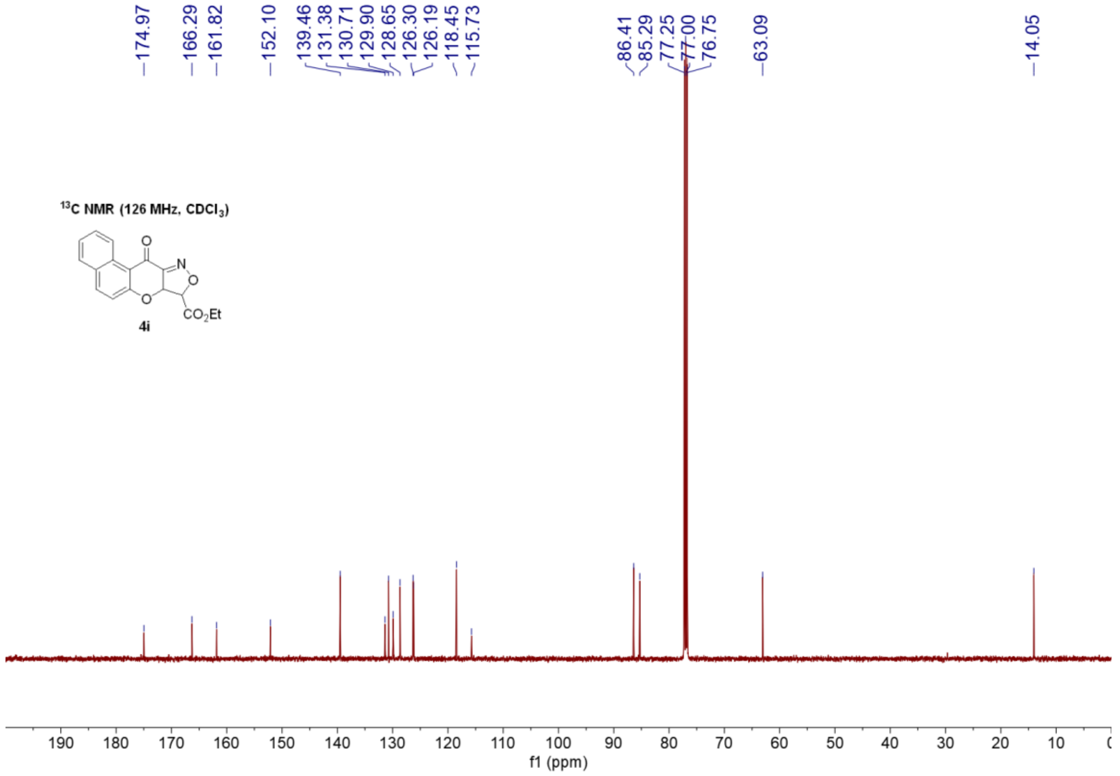

174.97
166.29
161.82
152.10
139.46
131.38
130.71
129.90
128.65
126.30
126.19
118.45
115.73
86.41
85.29
77.25
77.00
76.75
63.09
14.05
13C NMR (126 MHz, CDCl3)
4i
f1 (ppm)

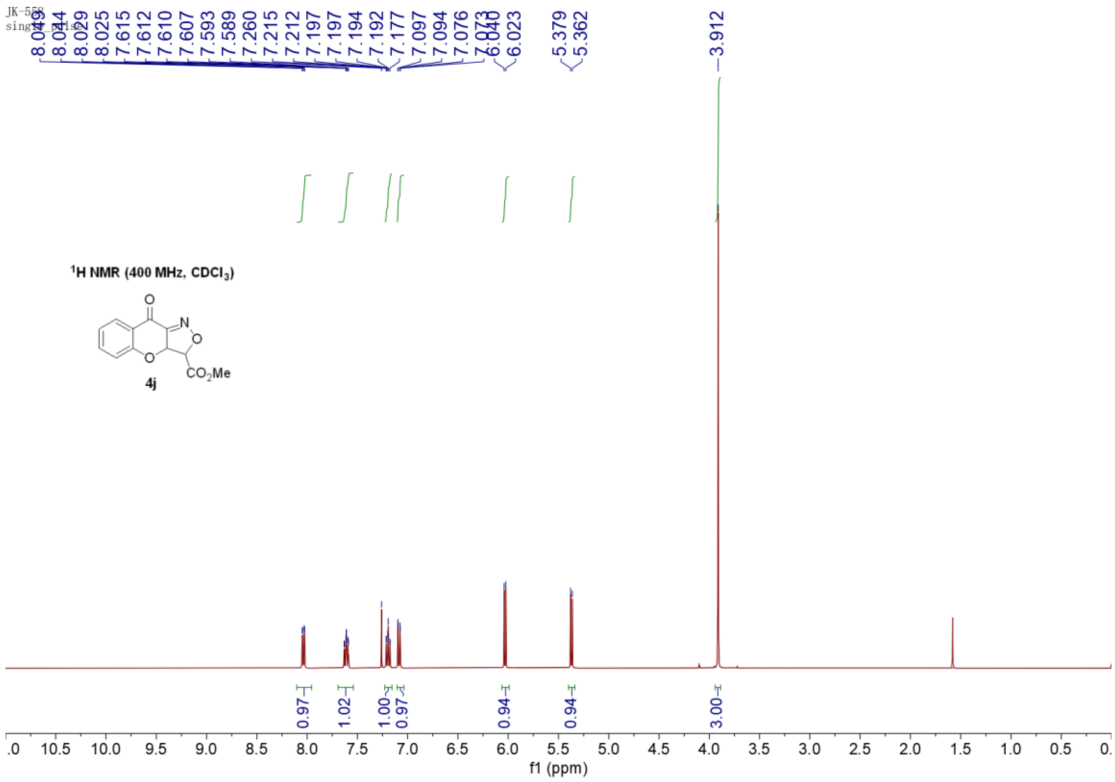

1H NMR (400 MHz, CDCl3)
4j
CO2Me
8.049
8.044
8.029
8.025
7.615
7.612
7.610
7.607
7.593
7.589
7.260
7.215
7.212
7.197
7.197
7.194
7.192
7.177
7.097
7.094
7.076
7.073
6.040
6.023
5.379
5.362
3.912
0.97
1.02
1.00
0.97
0.94
0.94
3.00
f1 (ppm)

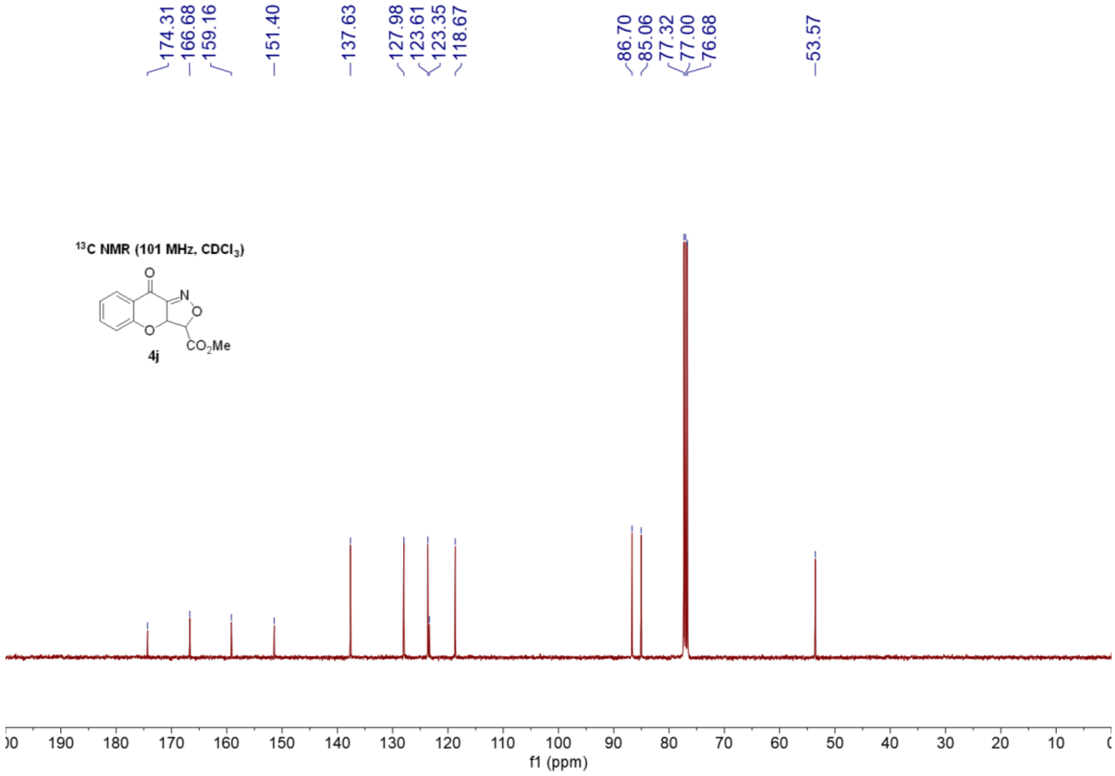

13C NMR (101 MHz, CDCl3)
4j
CO2Me
174.31
166.68
159.16
151.40
137.63
127.98
123.61
123.35
118.67
86.70
85.06
77.32
77.00
76.68
53.57
f1 (ppm)

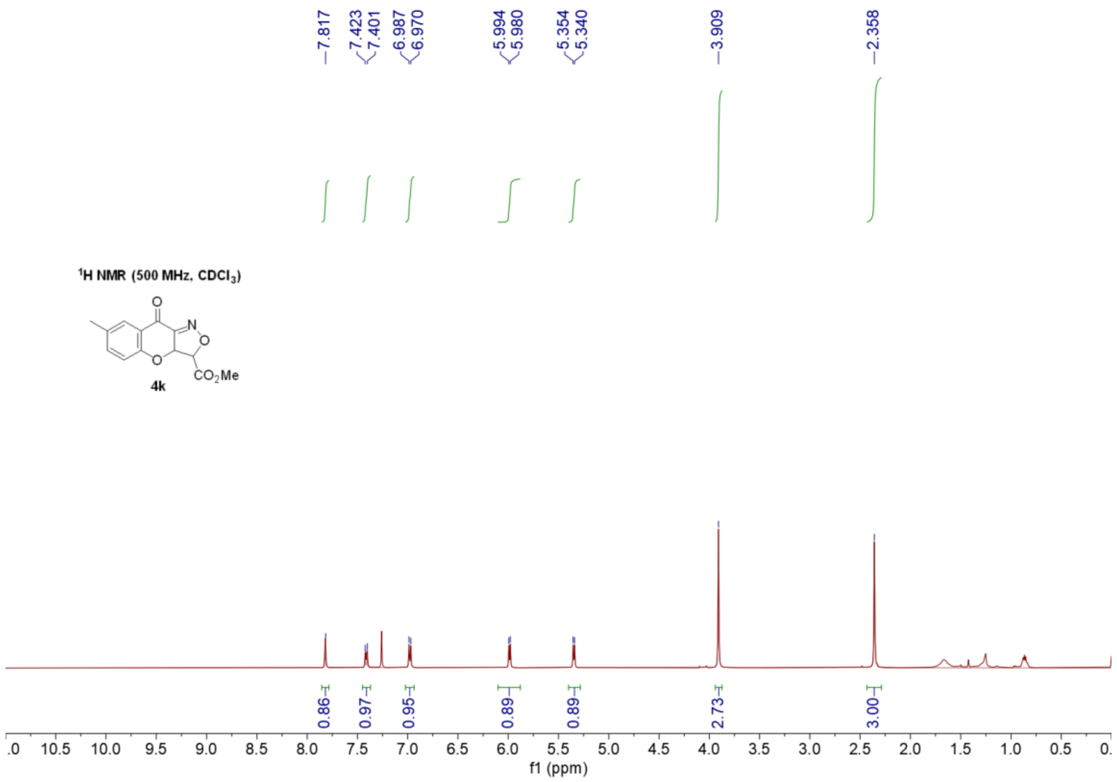

¹H NMR (500 MHz, CDCl₃)
4k
CO₂Me
7.817
7.423
7.401
6.987
6.970
5.994
5.980
5.354
5.340
3.909
2.358
0.86
0.97
0.95
0.89
0.89
2.73
3.00
f1 (ppm)

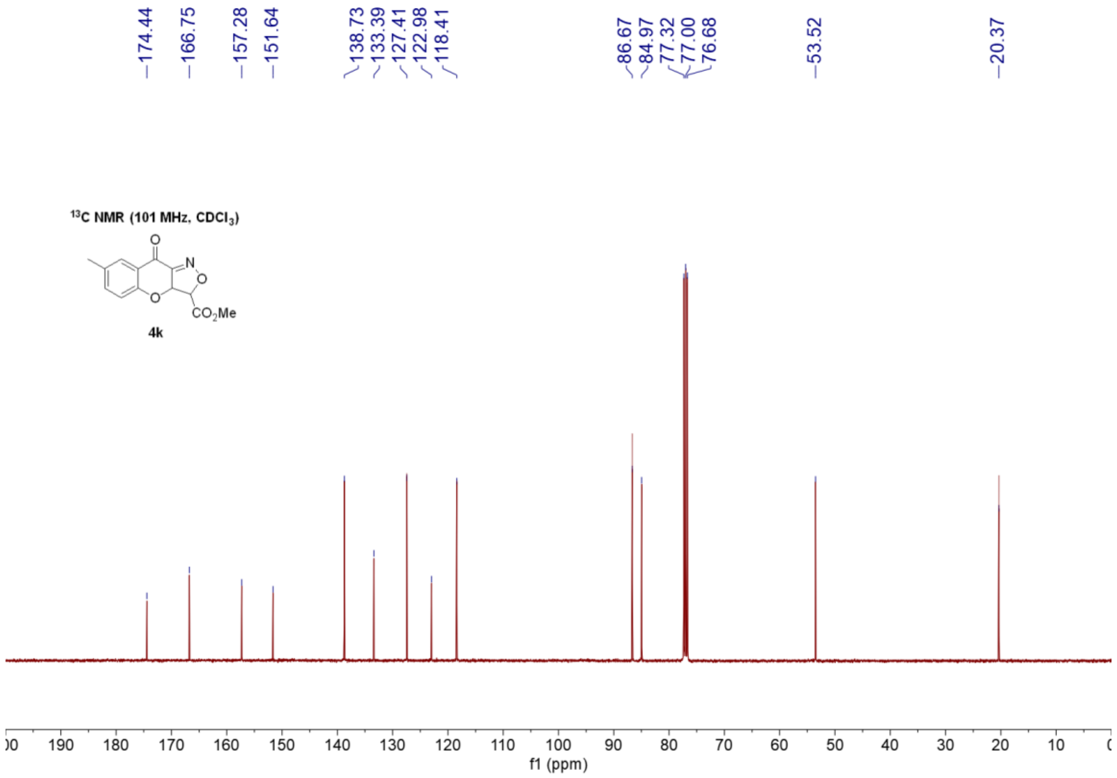

¹³C NMR (101 MHz, CDCl₃)
4k
CO₂Me
174.44
166.75
157.28
151.64
138.73
133.39
127.41
122.98
118.41
86.67
84.97
77.32
77.00
76.68
53.52
20.37
f1 (ppm)

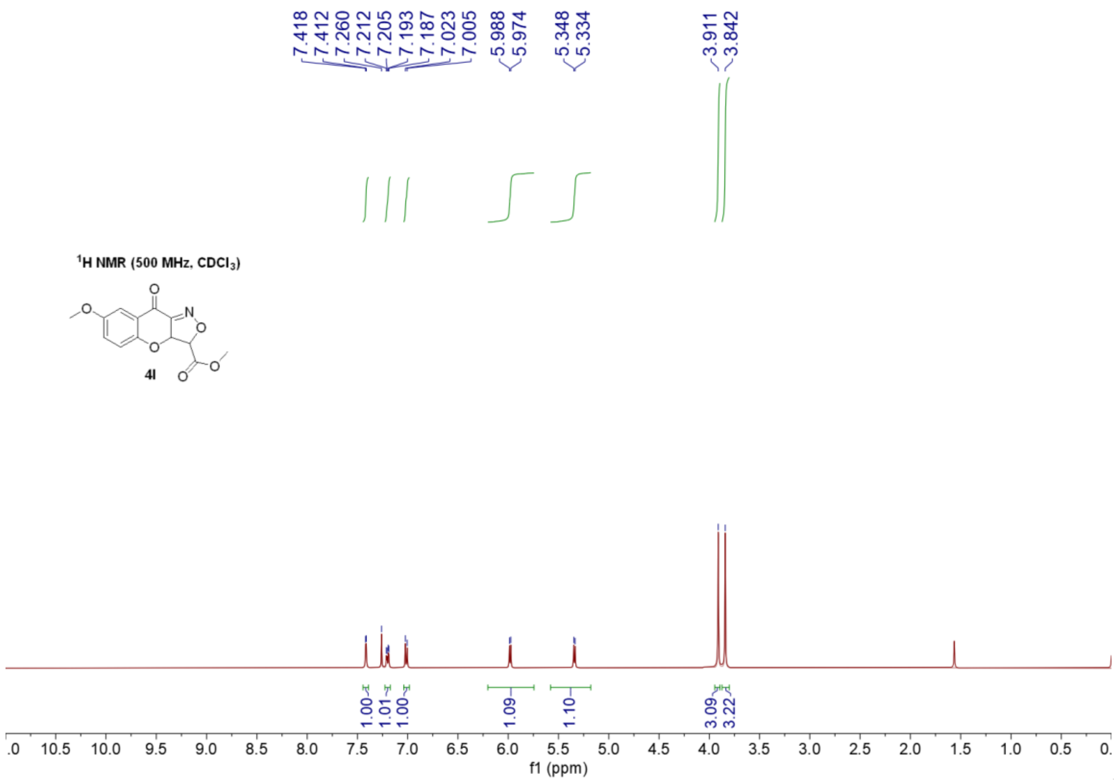
1H NMR (500 MHz, CDCl3)
4l
f1 (ppm)

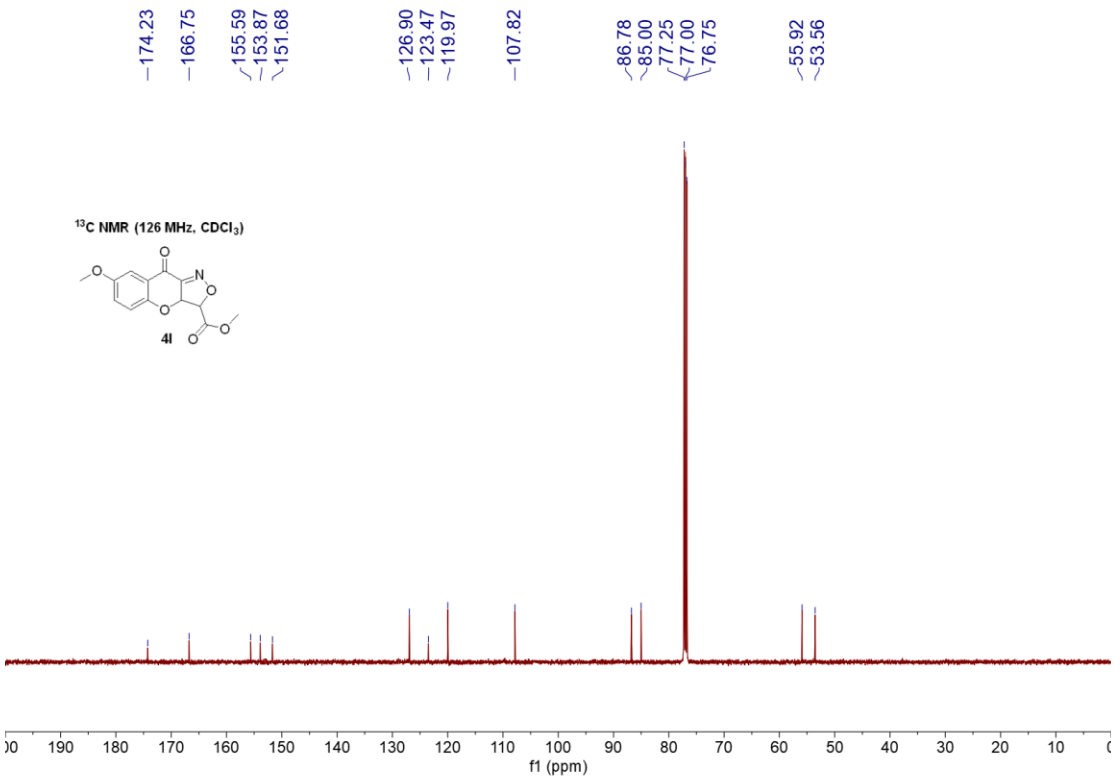
13C NMR (126 MHz, CDCl3)
4l
f1 (ppm)

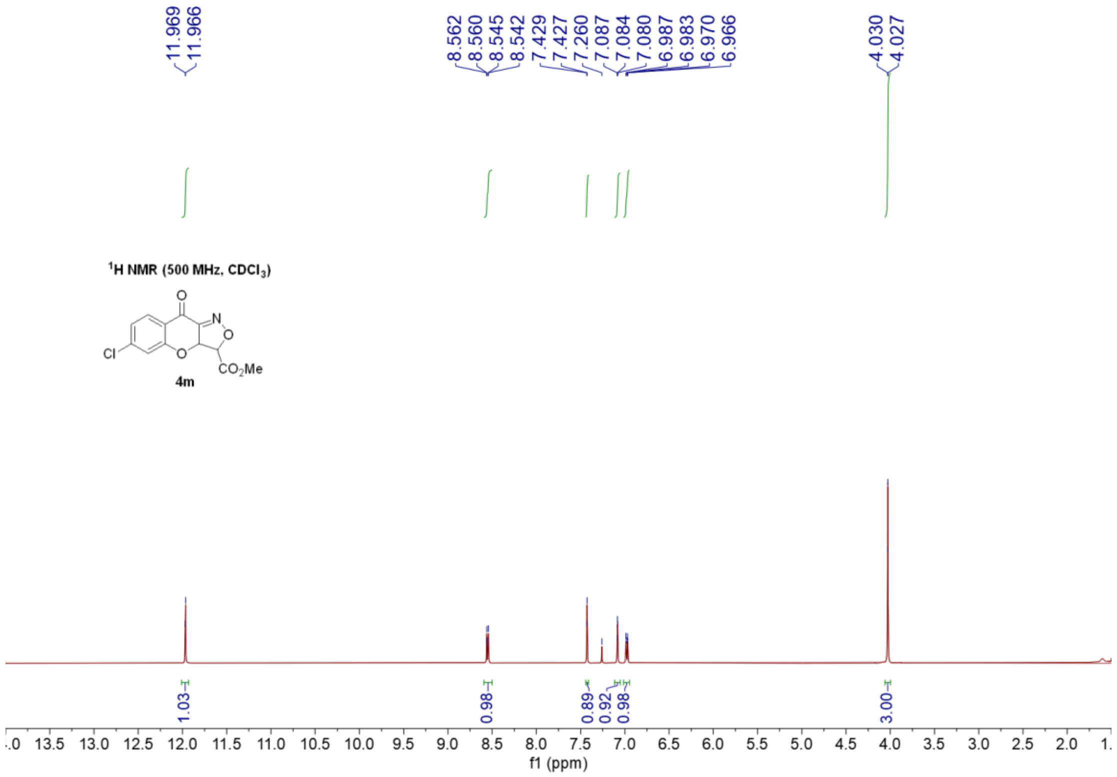
1H NMR (500 MHz, CDCl3)
4m
11.969
11.966
8.562
8.560
8.545
8.542
7.429
7.427
7.260
7.087
7.084
7.080
6.987
6.983
6.970
6.966
4.030
4.027
1.03
0.98
0.89
0.92
0.98
3.00
f1 (ppm)

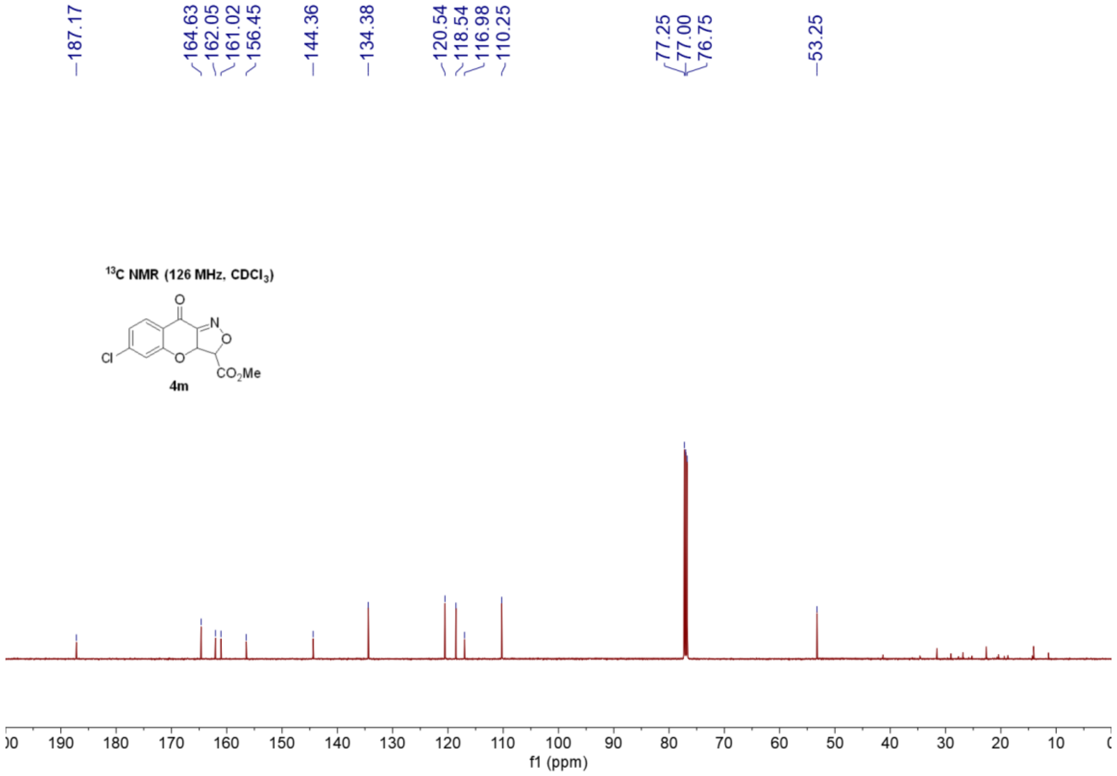
13C NMR (126 MHz, CDCl3)
4m
187.17
164.63
162.05
161.02
156.45
144.36
134.38
120.54
118.54
116.98
110.25
77.25
77.00
76.75
53.25
f1 (ppm)

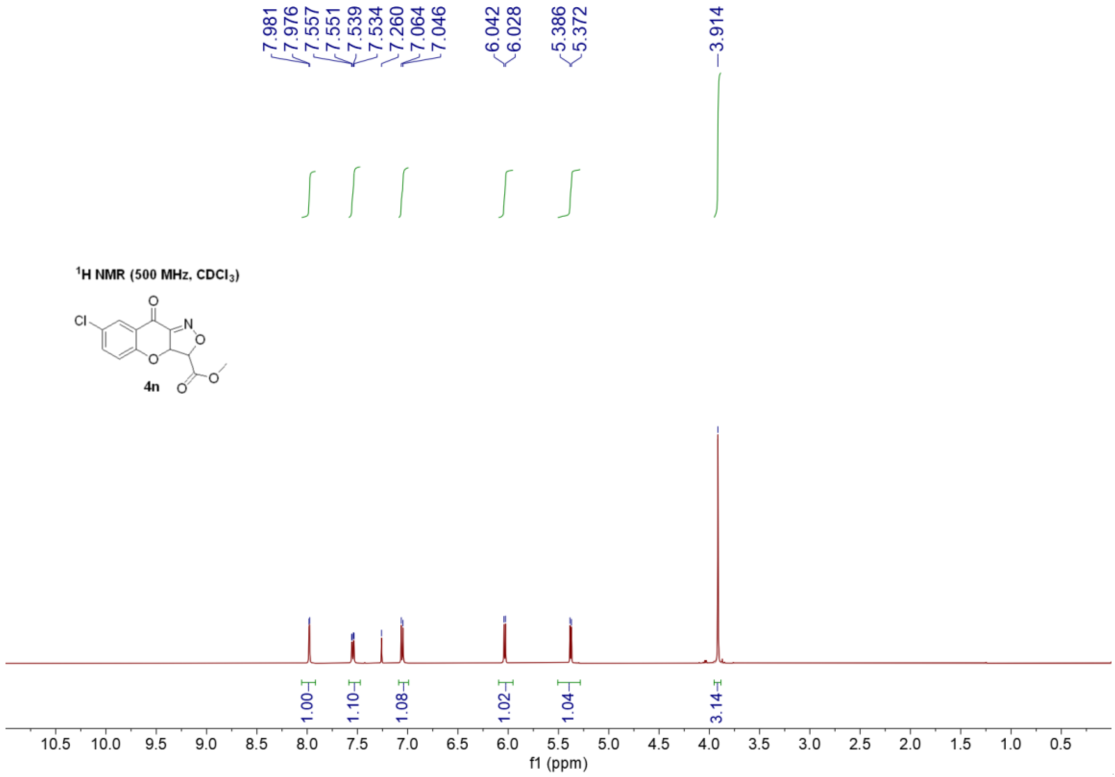

7.981
7.976
7.557
7.551
7.539
7.534
7.260
7.064
7.046
6.042
6.028
5.386
5.372
3.914
1H NMR (500 MHz, CDCl3)
4n
1.00
1.10
1.08
1.02
1.04
3.14
f1 (ppm)

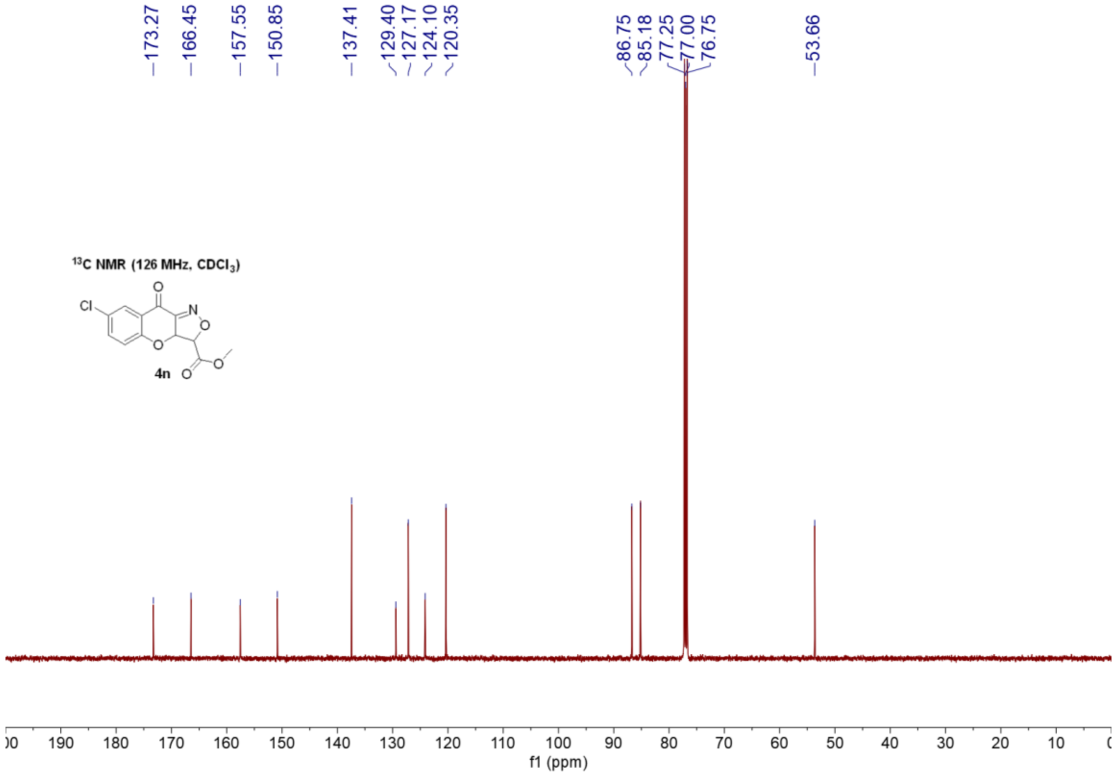

173.27
166.45
157.55
150.85
137.41
129.40
127.17
124.10
120.35
86.75
85.18
77.25
77.00
76.75
53.66
13C NMR (126 MHz, CDCl3)
4n
f1 (ppm)

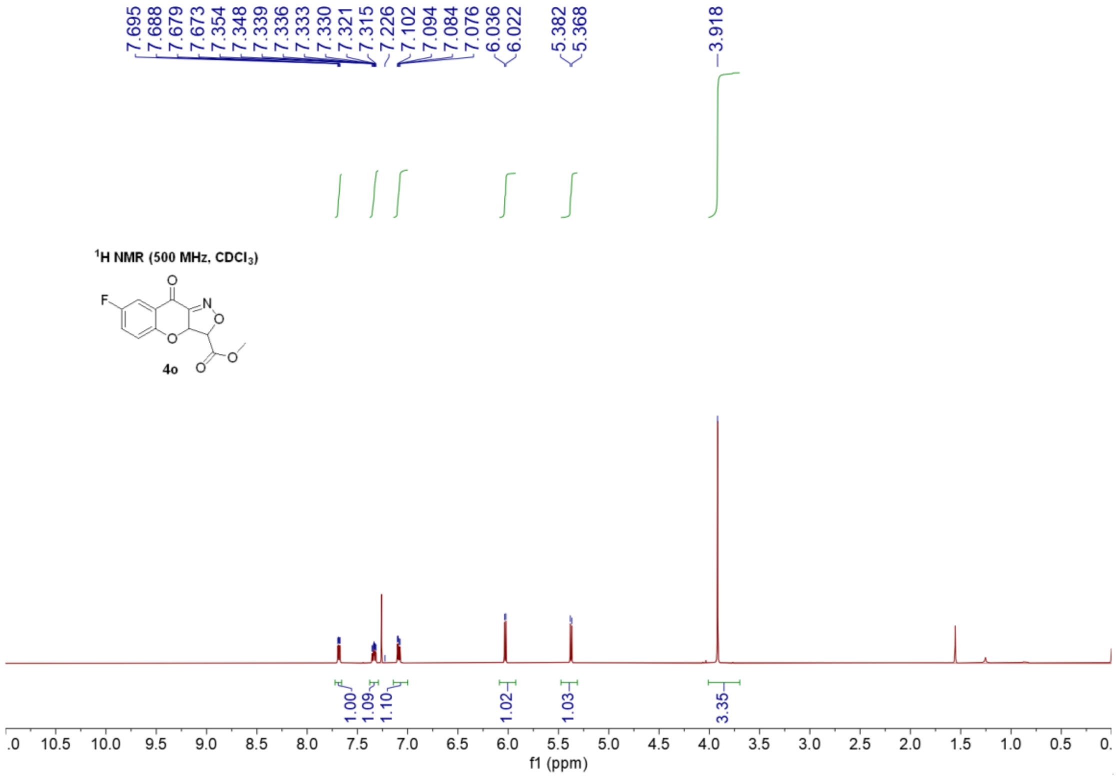
7.695
7.688
7.679
7.673
7.354
7.348
7.339
7.336
7.333
7.330
7.321
7.315
7.226
7.102
7.094
7.084
7.076
6.036
6.022
5.382
5.368
3.918
1H NMR (500 MHz, CDCl3)
F
O
N
O
O
O
O
4o
1.00
1.09
1.10
1.02
1.03
3.35
f1 (ppm)

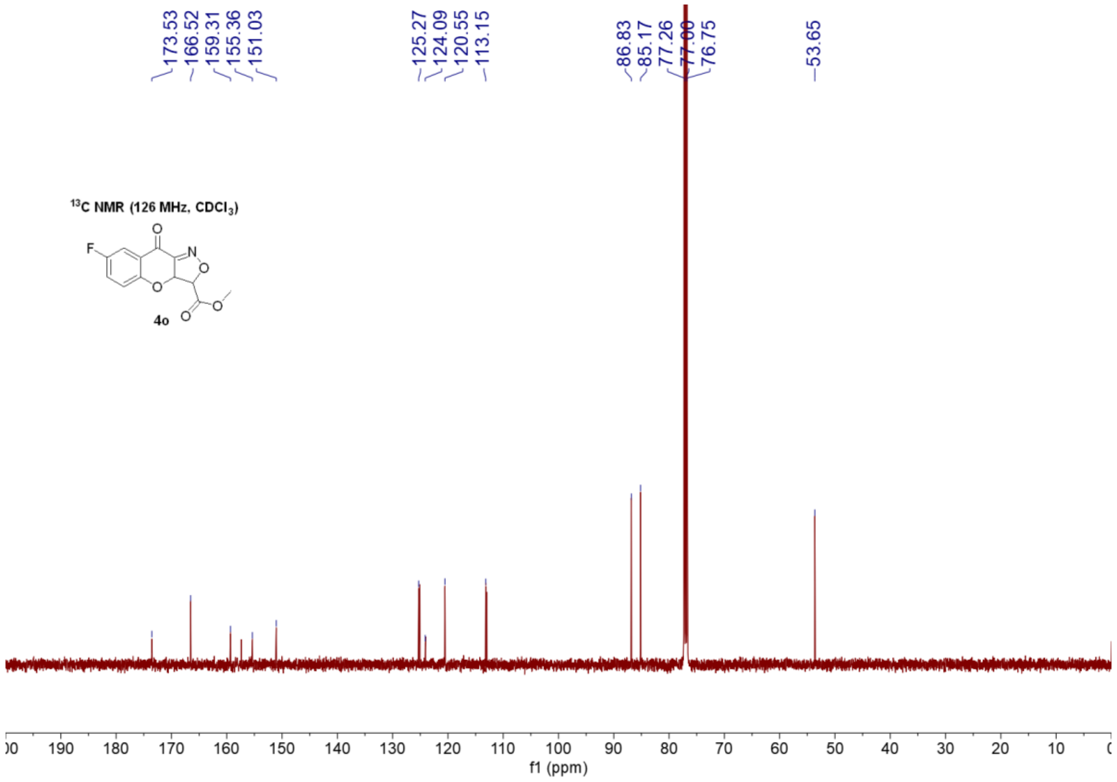
173.53
166.52
159.31
155.36
151.03
125.27
124.09
120.55
113.15
86.83
85.17
77.26
77.00
76.75
53.65
13C NMR (126 MHz, CDCl3)
F
O
N
O
O
O
O
4o
f1 (ppm)

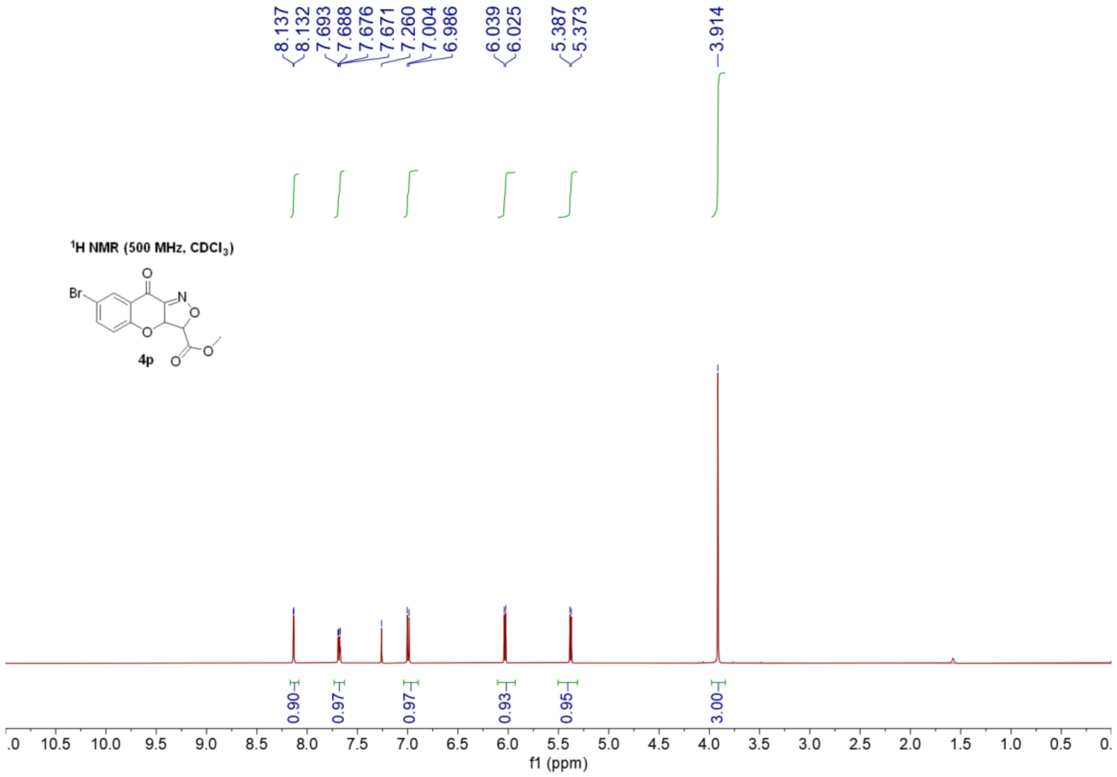
8.137
8.132
7.693
7.688
7.676
7.671
7.260
7.004
6.986
6.039
6.025
5.387
5.373
3.914
^{1}H NMR (500 MHz, $CDCl_3$)
Br
O
N
O
O
4p
O
O
0.90
0.97
0.97
0.93
0.95
3.00
f1 (ppm)

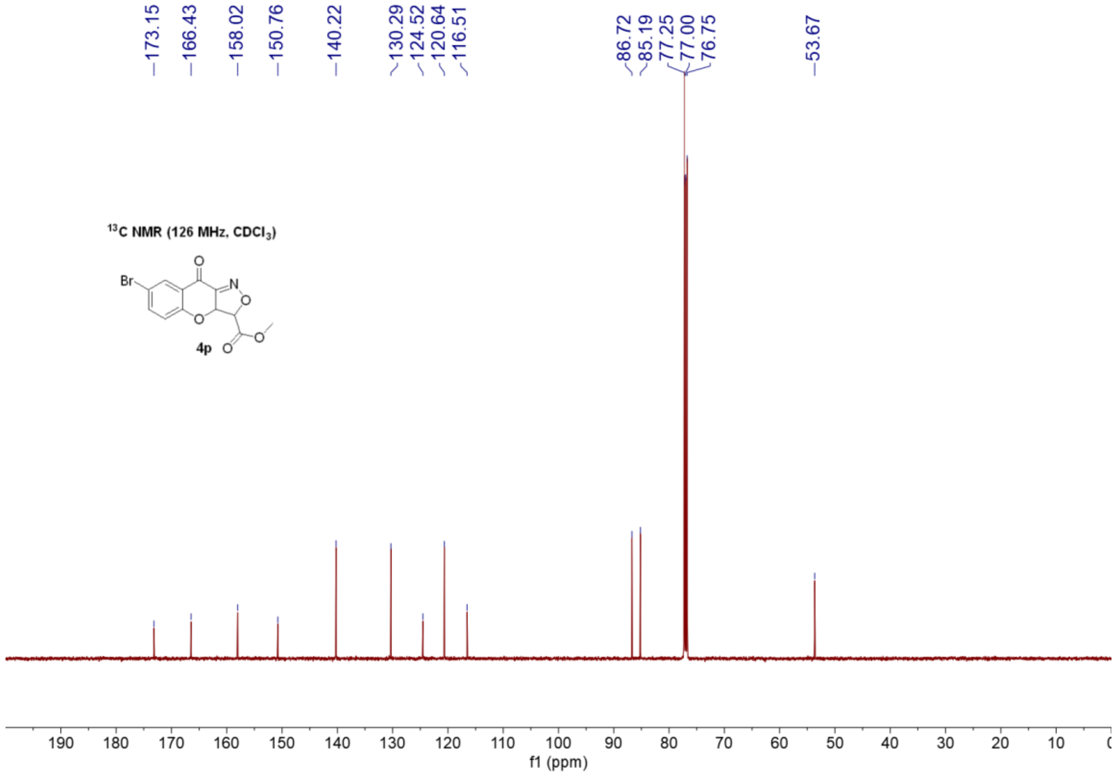
173.15
166.43
158.02
150.76
140.22
130.29
124.52
120.64
116.51
86.72
85.19
77.25
77.00
76.75
53.67
^{13}C NMR (126 MHz, $CDCl_3$)
Br
O
N
O
O
4p
O
O
f1 (ppm)

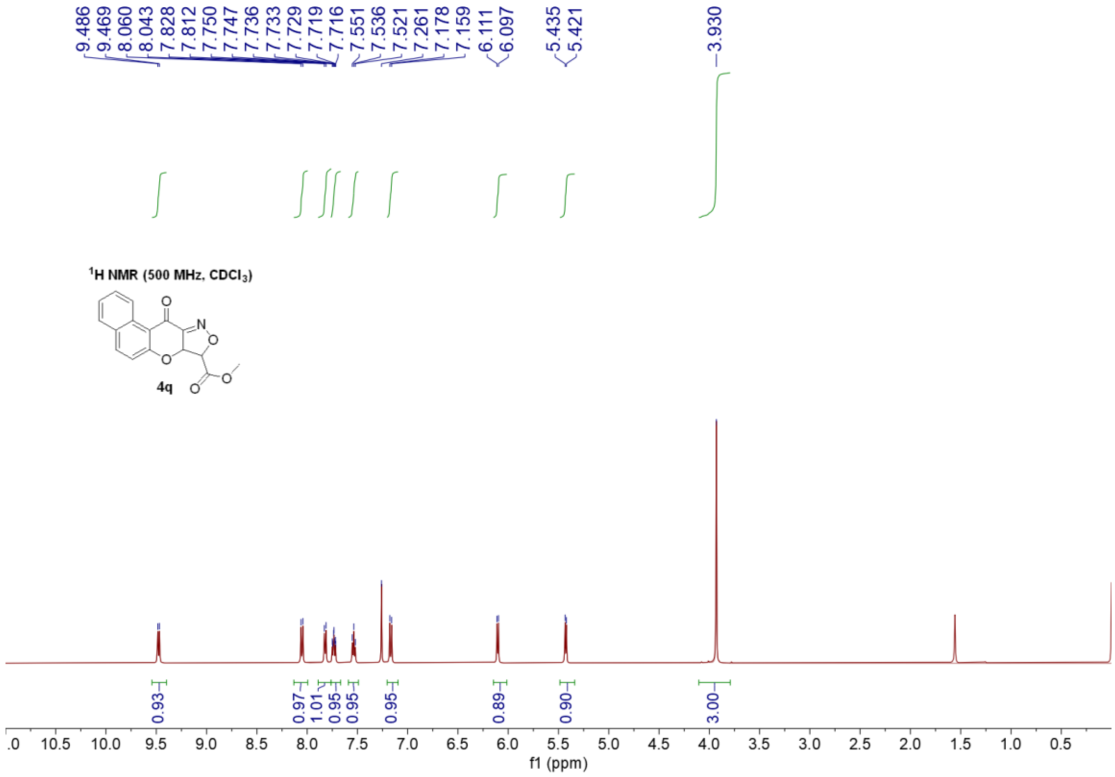
9.486 9.469 8.060 8.043 7.828 7.812 7.750 7.747 7.736 7.733 7.729 7.719 7.716 7.551 7.536 7.521 7.261 7.178 7.159 6.111 6.097 5.435 5.421
3.930
1H NMR (500 MHz, CDCl3)
O
N
O
O
4q
O
O
0.93
0.97
1.01
0.95
0.95
0.95
0.89
0.90
3.00
.0 10.5 10.0 9.5 9.0 8.5 8.0 7.5 7.0 6.5 6.0 5.5 5.0 4.5 4.0 3.5 3.0 2.5 2.0 1.5 1.0 0.5
f1 (ppm)

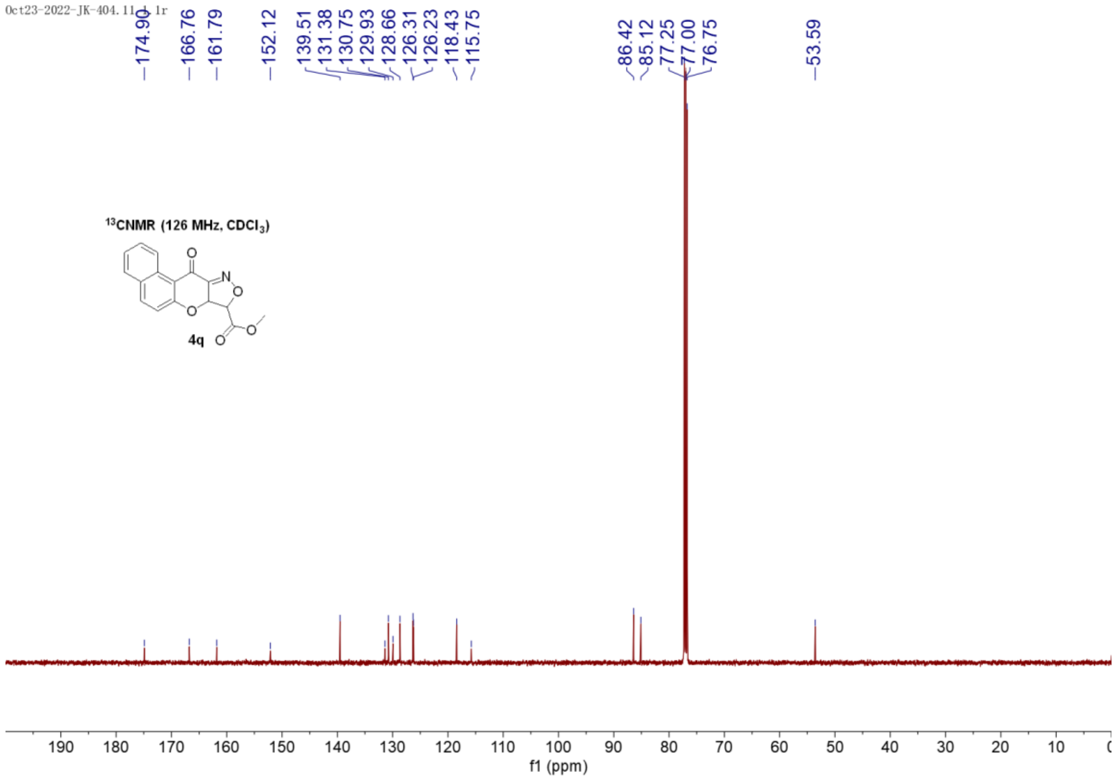
Oct23-2022-JK-404.11.1.1r
174.90
166.76
161.79
152.12
139.51 131.38 130.75 129.93 128.66 126.31 126.23 118.43 115.75
86.42 85.12 77.25 77.00 76.75
53.59
13CNMR (126 MHz, CDCl3)
O
N
O
O
4q
O
O
190 180 170 160 150 140 130 120 110 100 90 80 70 60 50 40 30 20 10
f1 (ppm)

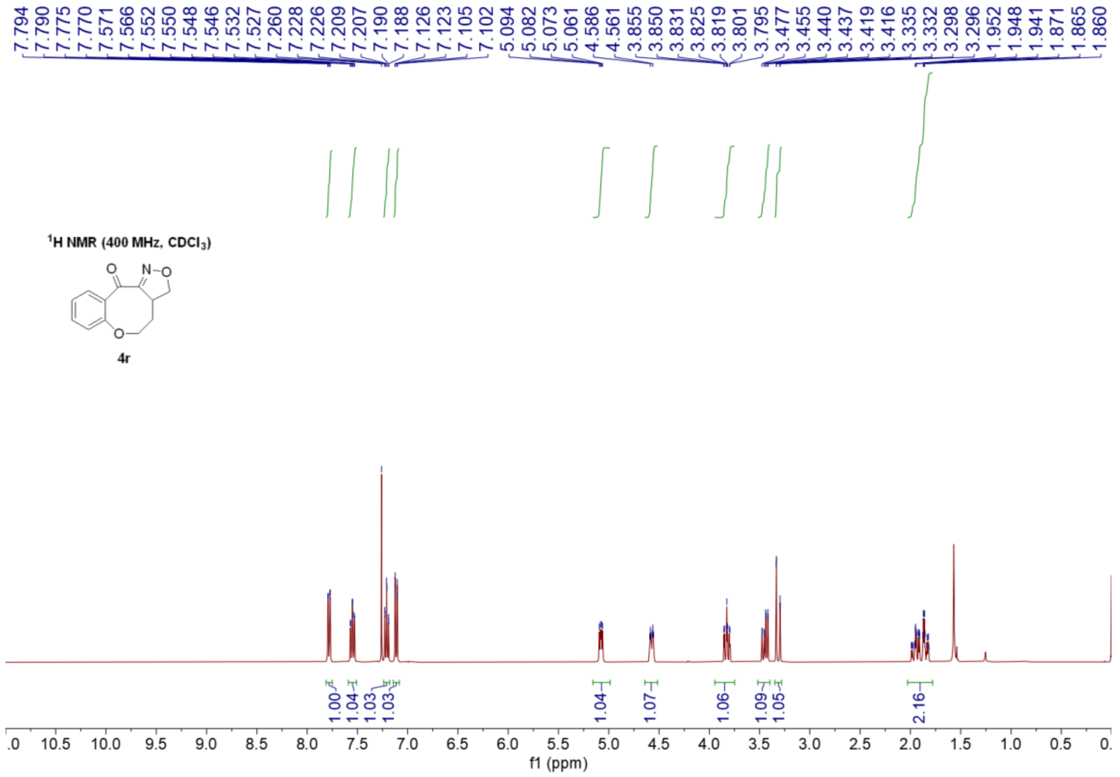

7.794 7.790 7.775 7.770 7.571 7.566 7.552 7.550 7.548 7.546 7.532 7.527 7.260 7.228 7.226 7.209 7.207 7.190 7.188 7.126 7.123 7.105 7.102 5.094 5.082 5.073 5.061 4.586 4.561 3.855 3.850 3.831 3.825 3.819 3.801 3.795 3.477 3.455 3.440 3.437 3.419 3.416 3.335 3.332 3.298 3.296 1.952 1.948 1.941 1.871 1.865 1.860
1H NMR (400 MHz, CDCl3)
N–O
O
4r
1.00 1.04 1.03 1.03 1.04 1.07 1.06 1.09 1.05 2.16
f1 (ppm)

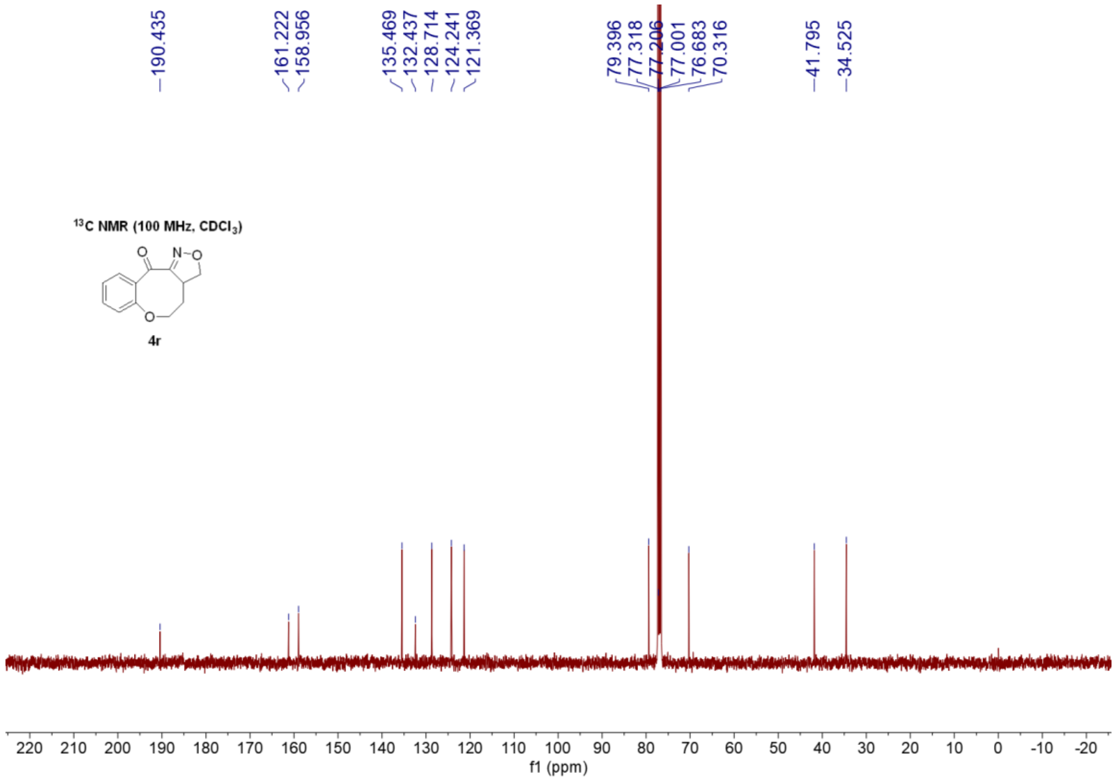

190.435
161.222 158.956
135.469 132.437 128.714 124.241 121.369
79.396 77.318 77.206 77.001 76.683 70.316
41.795 34.525
13C NMR (100 MHz, CDCl3)
N–O
O
4r
f1 (ppm)

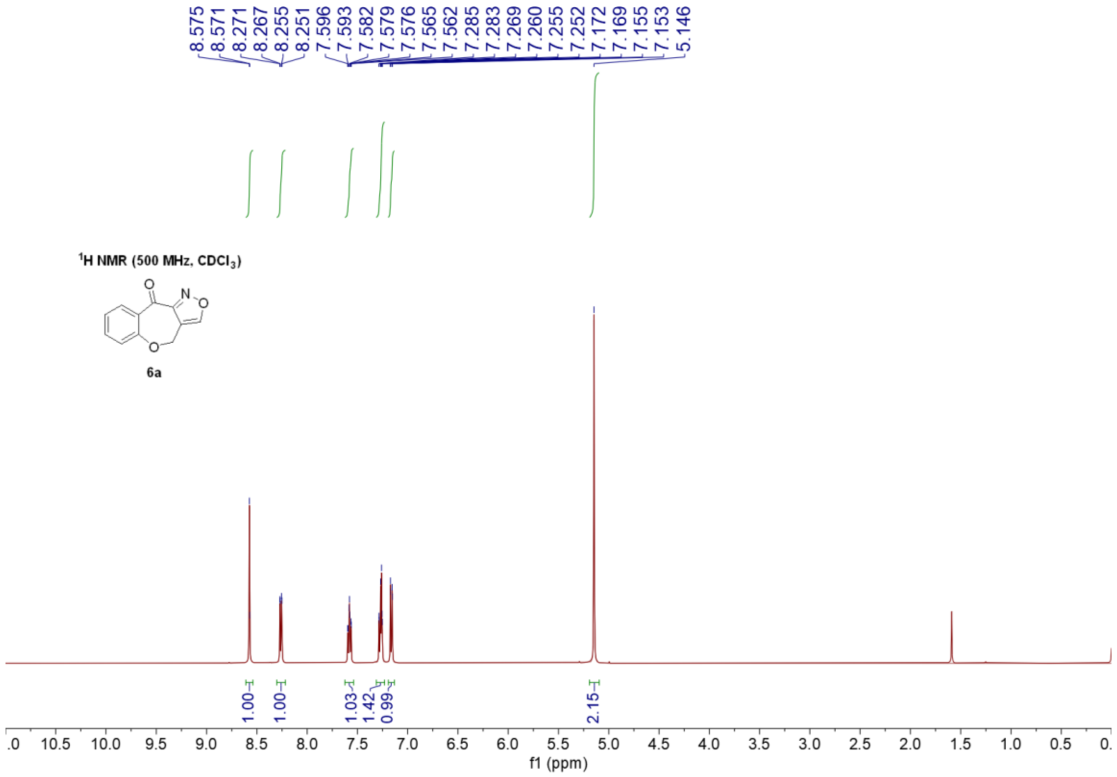
8.575
8.571
8.271
8.267
8.255
8.251
7.596
7.593
7.582
7.579
7.576
7.565
7.562
7.285
7.283
7.269
7.260
7.255
7.252
7.172
7.169
7.155
7.153
5.146
^1H NMR (500 MHz, $CDCl_3$)
6a
1.00
1.00
1.03
1.42
0.99
2.15
f1 (ppm)

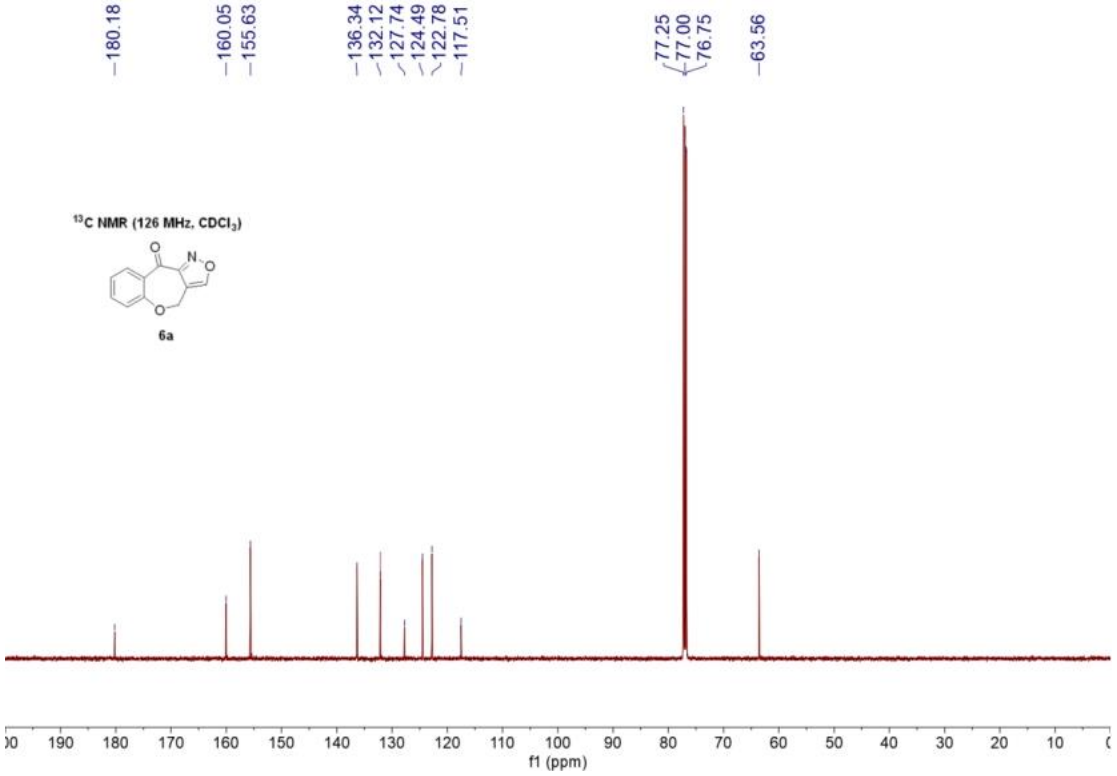
180.18
160.05
155.63
136.34
132.12
127.74
124.49
122.78
117.51
77.25
77.00
76.75
63.56
^{13}C NMR (126 MHz, $CDCl_3$)
6a
f1 (ppm)

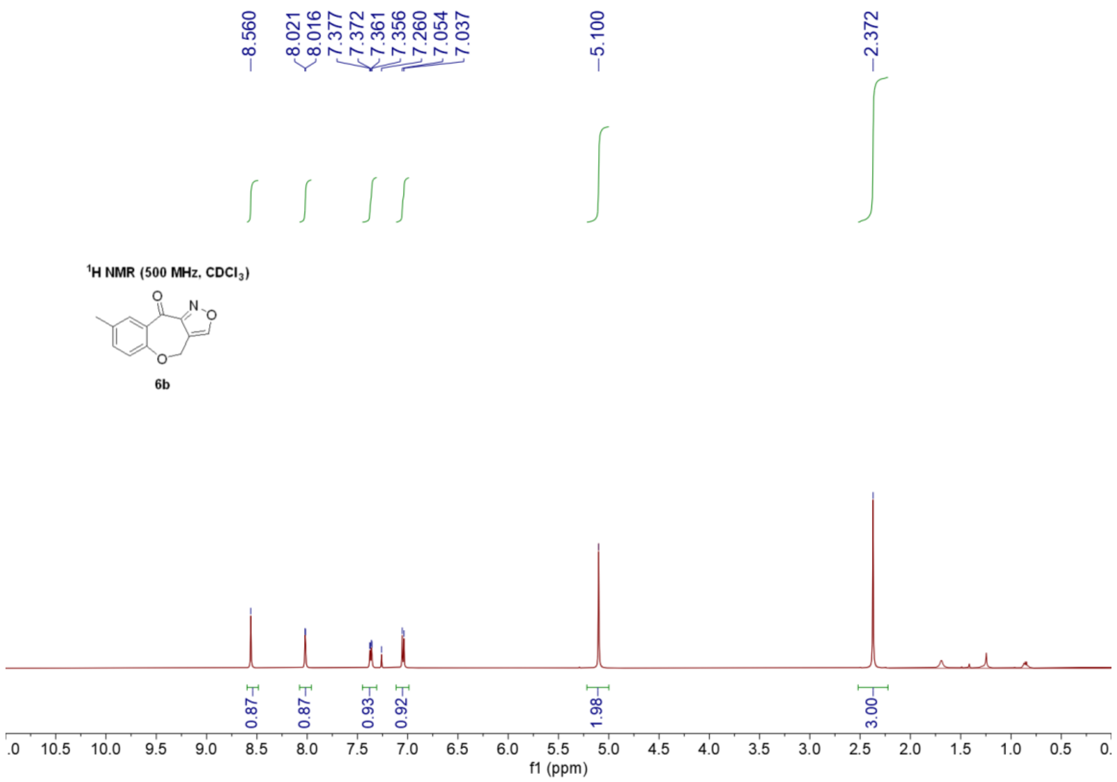

^{1}H NMR (500 MHz, CDCl$_3$)
6b
8.560
8.021
8.016
7.377
7.372
7.361
7.356
7.260
7.054
7.037
5.100
2.372
0.87
0.87
0.93
0.92
1.98
3.00
f1 (ppm)

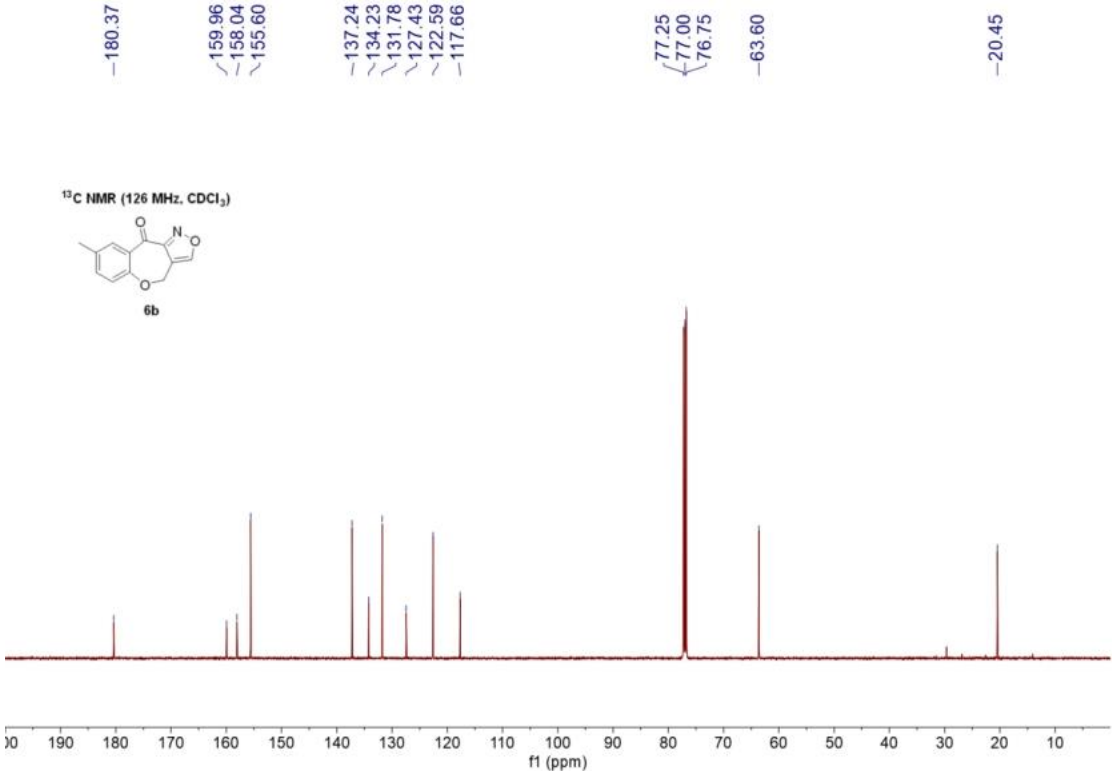

^{13}C NMR (126 MHz, CDCl$_3$)
6b
180.37
159.96
158.04
155.60
137.24
134.23
131.78
127.43
122.59
117.66
77.25
77.00
76.75
63.60
20.45
f1 (ppm)

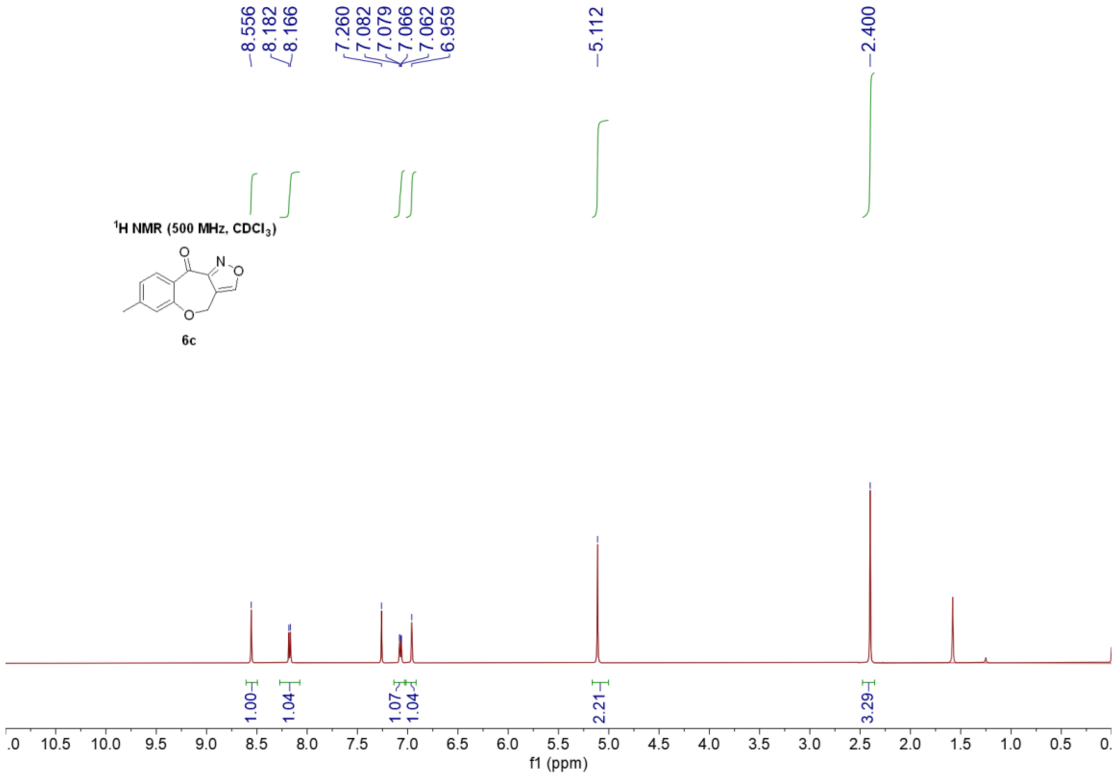
8.556
8.182
8.166
7.260
7.082
7.079
7.066
7.062
6.959
5.112
2.400
1H NMR (500 MHz, CDCl3)
6c
1.00
1.04
1.07
1.04
2.21
3.29
f1 (ppm)

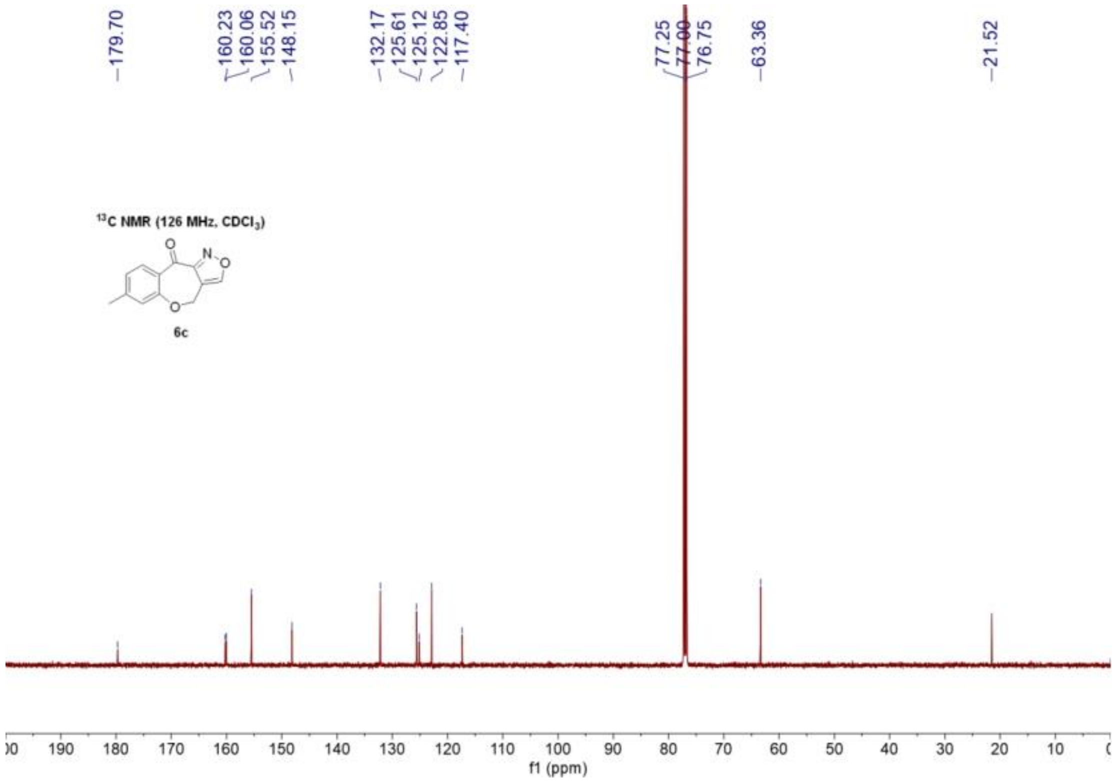
179.70
160.23
160.06
155.52
148.15
132.17
125.61
125.12
122.85
117.40
77.25
77.00
76.75
63.36
21.52
13C NMR (126 MHz, CDCl3)
6c
f1 (ppm)

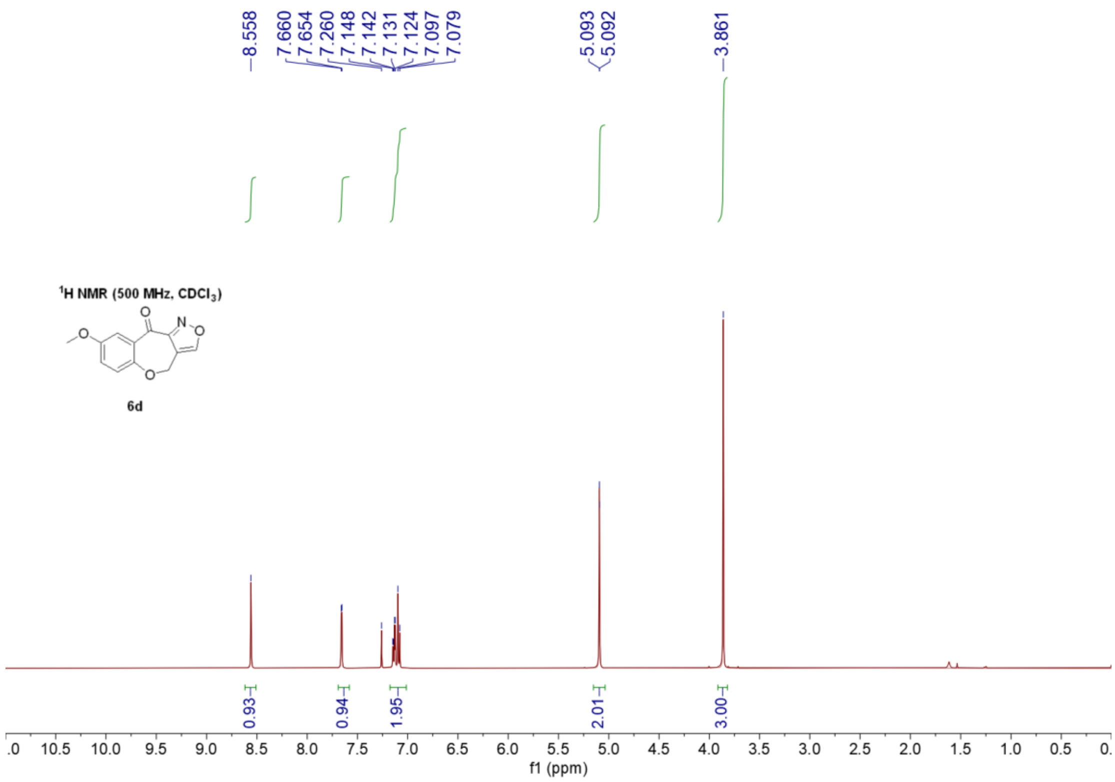

8.558
7.660
7.654
7.260
7.148
7.142
7.131
7.124
7.097
7.079
5.093
5.092
3.861
1H NMR (500 MHz, CDCl3)
6d
0.93
0.94
1.95
2.01
3.00
f1 (ppm)

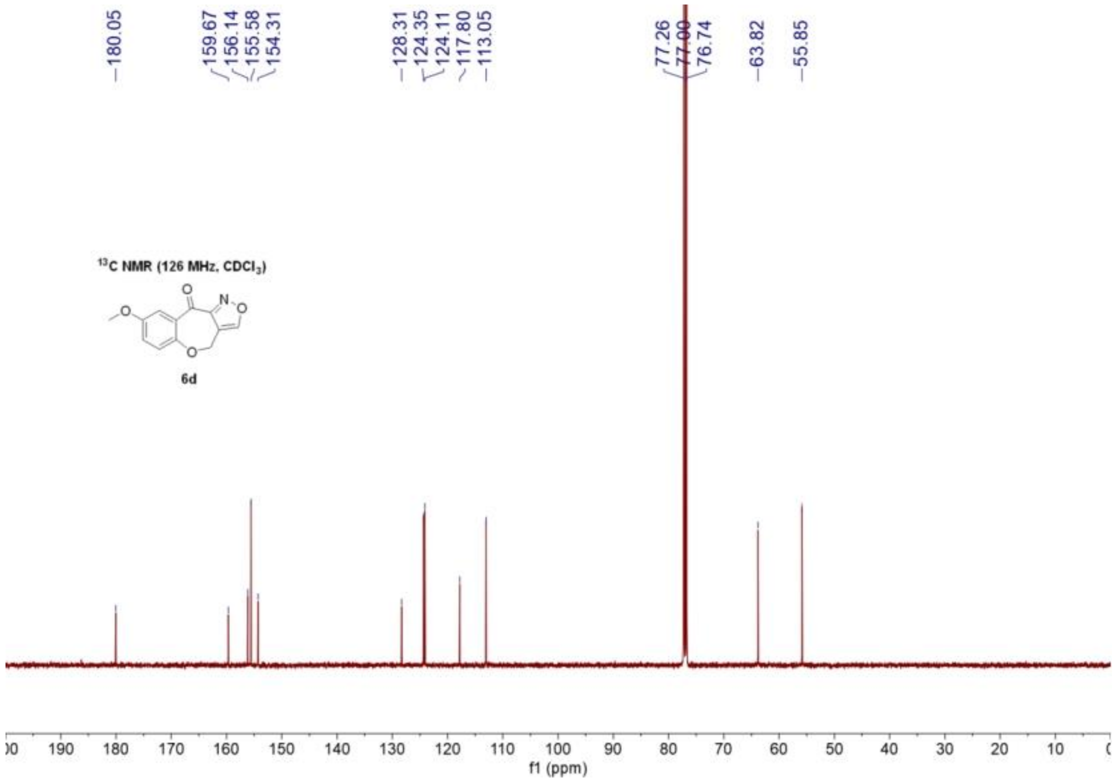

180.05
159.67
156.14
155.58
154.31
128.31
124.35
124.11
117.80
113.05
77.26
77.00
76.74
63.82
55.85
13C NMR (126 MHz, CDCl3)
6d
f1 (ppm)

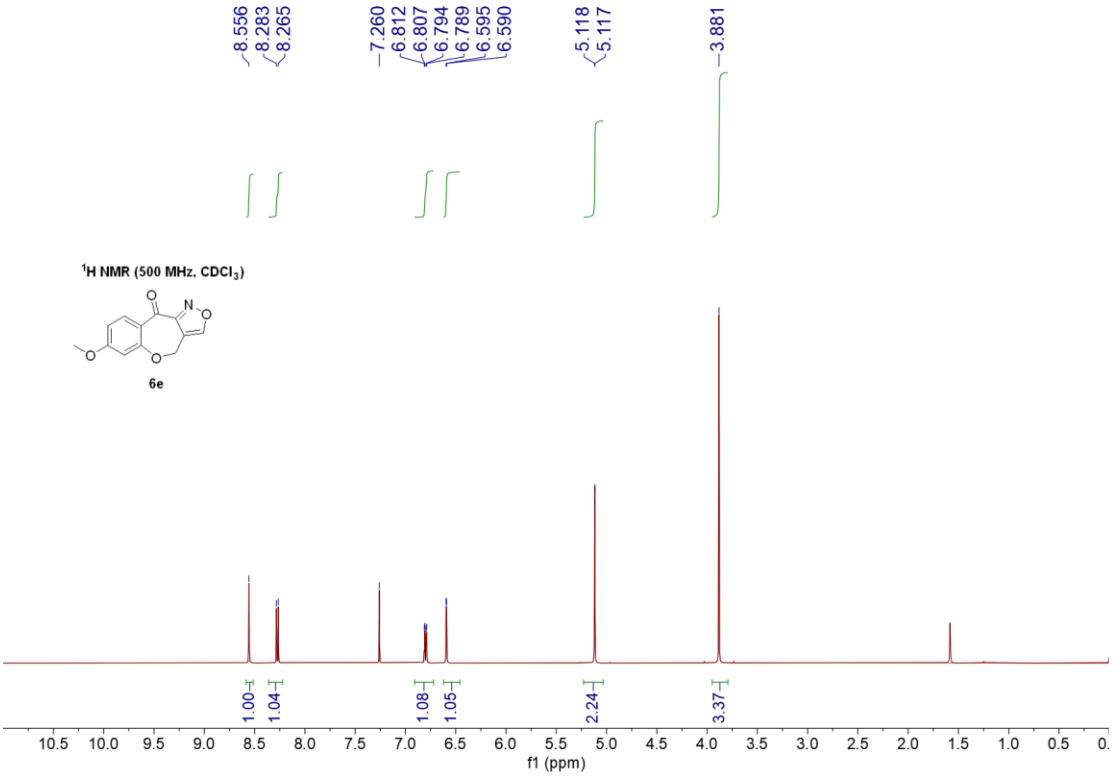
8.556
8.283
8.265
7.260
6.812
6.807
6.794
6.789
6.595
6.590
5.118
5.117
3.881
1H NMR (500 MHz, CDCl3)
O
N
O
O
O
6e
1.00
1.04
1.08
1.05
2.24
3.37
f1 (ppm)

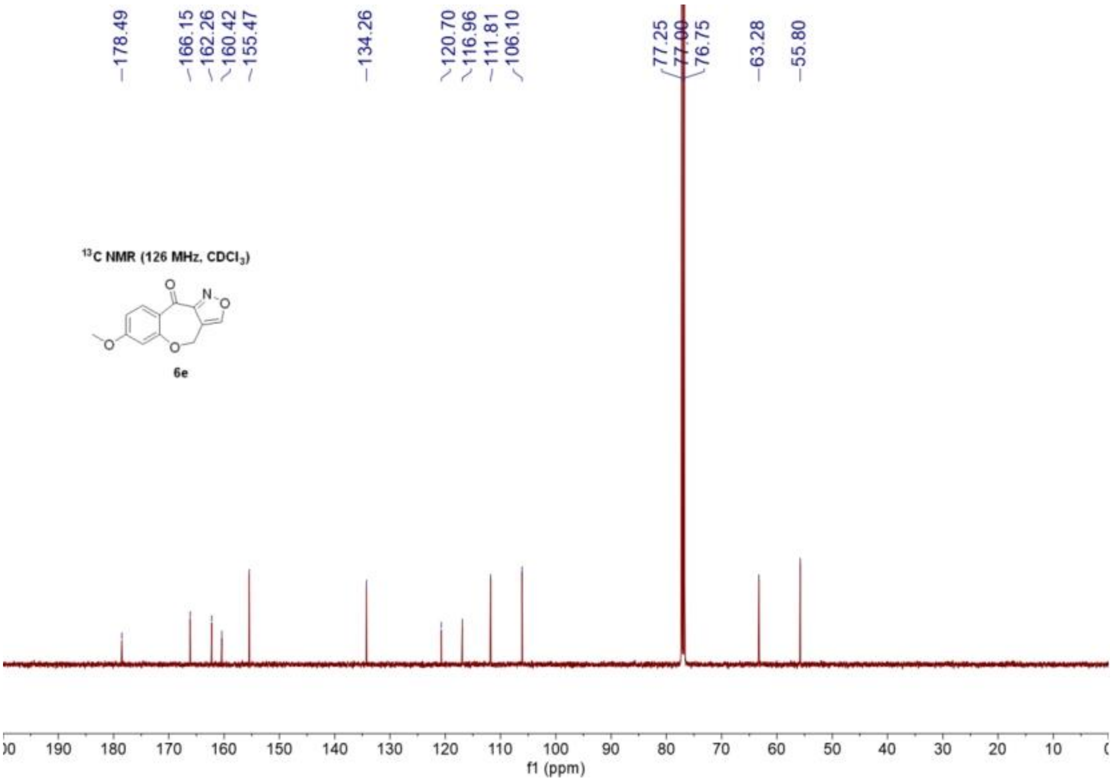
178.49
166.15
162.26
160.42
155.47
134.26
120.70
116.96
111.81
106.10
77.25
77.00
76.75
63.28
55.80
13C NMR (126 MHz, CDCl3)
O
N
O
O
O
6e
f1 (ppm)

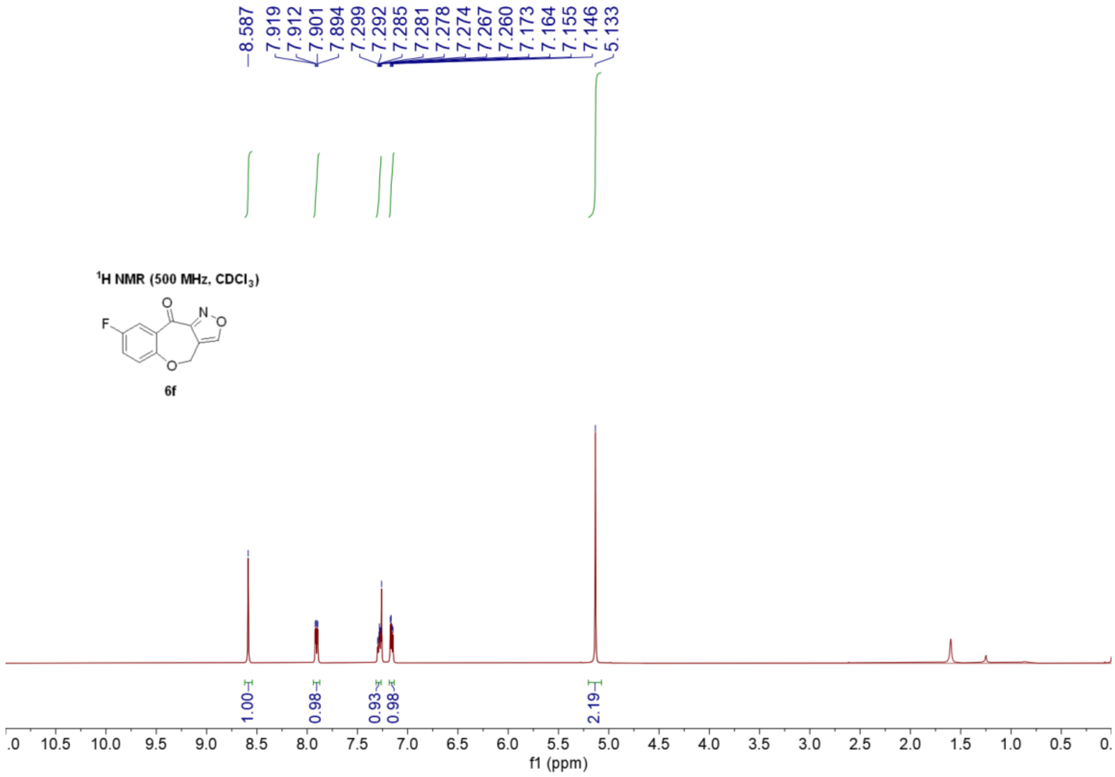
8.587
7.919
7.912
7.901
7.894
7.299
7.292
7.285
7.281
7.278
7.274
7.267
7.260
7.173
7.164
7.155
7.146
5.133
¹H NMR (500 MHz, CDCl₃)
6f
1.00
0.98
0.93
0.98
2.19
f1 (ppm)

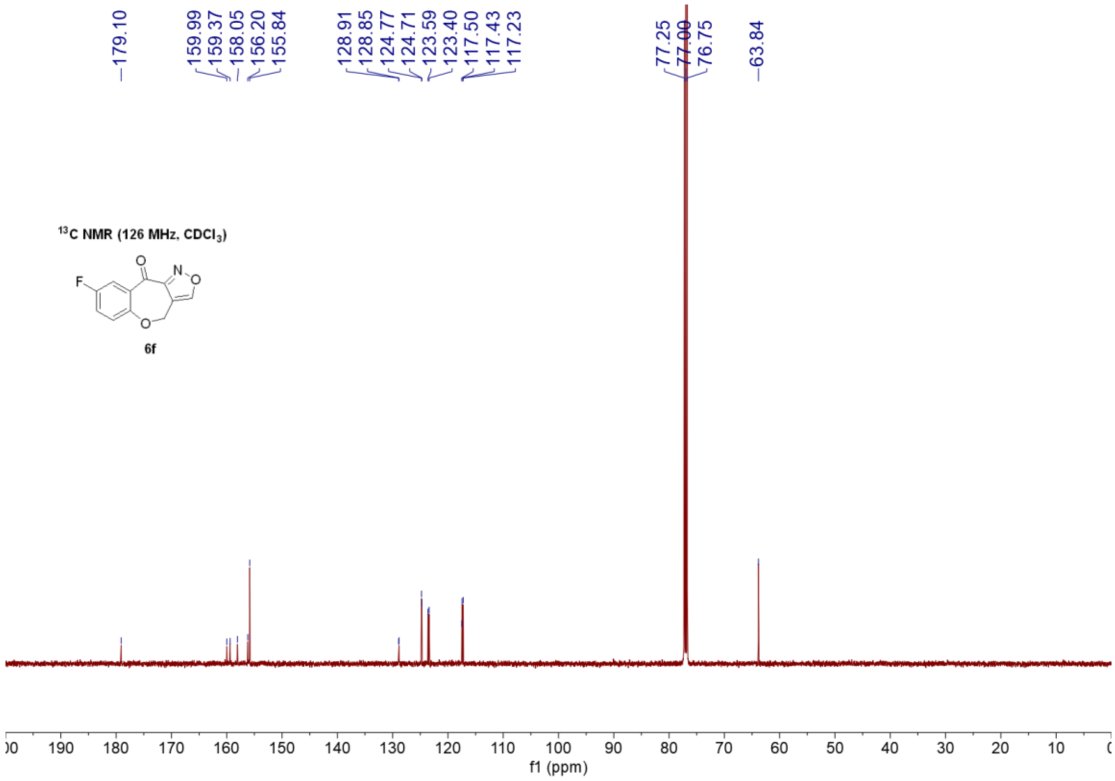
179.10
159.99
159.37
158.05
156.20
155.84
128.91
128.85
124.77
124.71
123.59
123.40
117.50
117.43
117.23
77.25
77.00
76.75
63.84
¹³C NMR (126 MHz, CDCl₃)
6f
f1 (ppm)

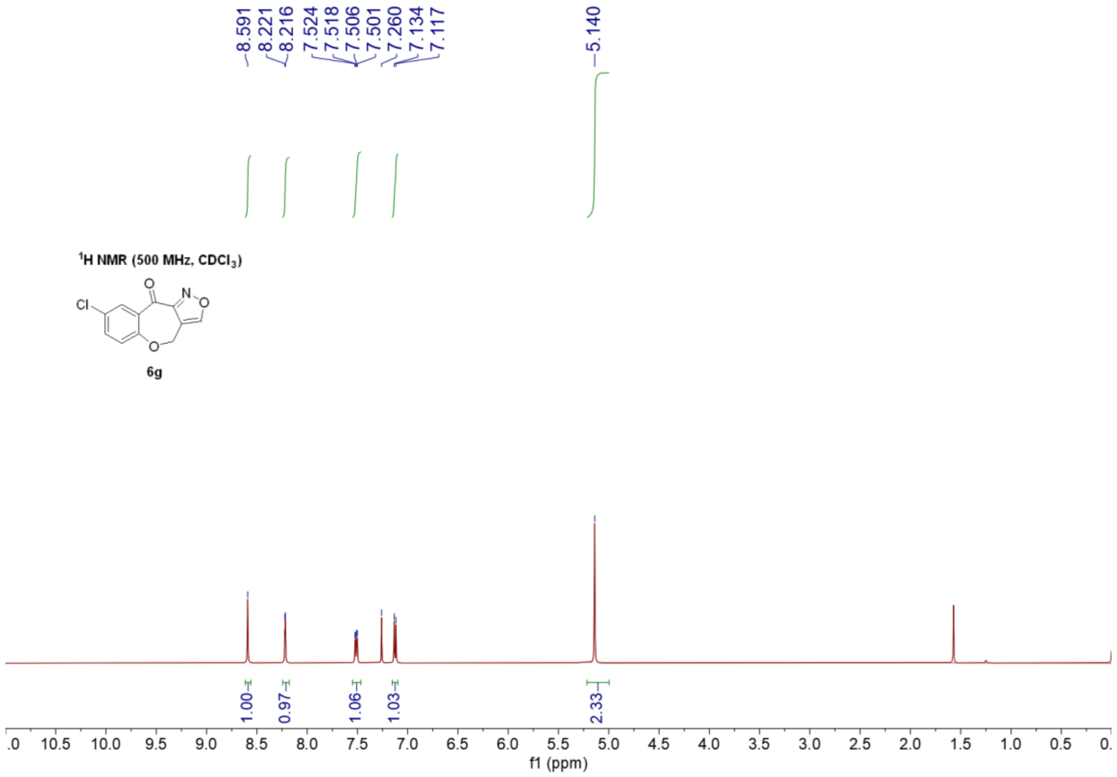
8.591
8.221
8.216
7.524
7.518
7.506
7.501
7.260
7.134
7.117
5.140
1H NMR (500 MHz, CDCl3)
Cl
O
N
O
O
6g
1.00
0.97
1.06
1.03
2.33
f1 (ppm)

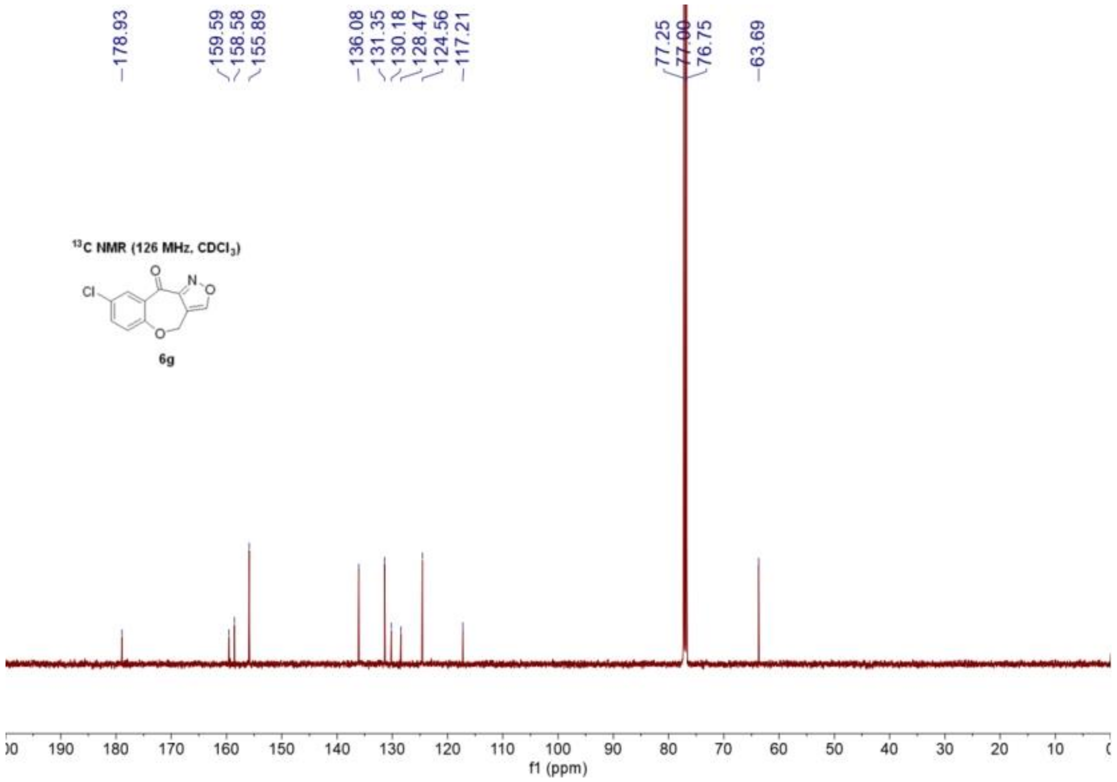
178.93
159.59
158.58
155.89
136.08
131.35
130.18
128.47
124.56
117.21
77.25
77.00
76.75
63.69
13C NMR (126 MHz, CDCl3)
Cl
O
N
O
O
6g
f1 (ppm)

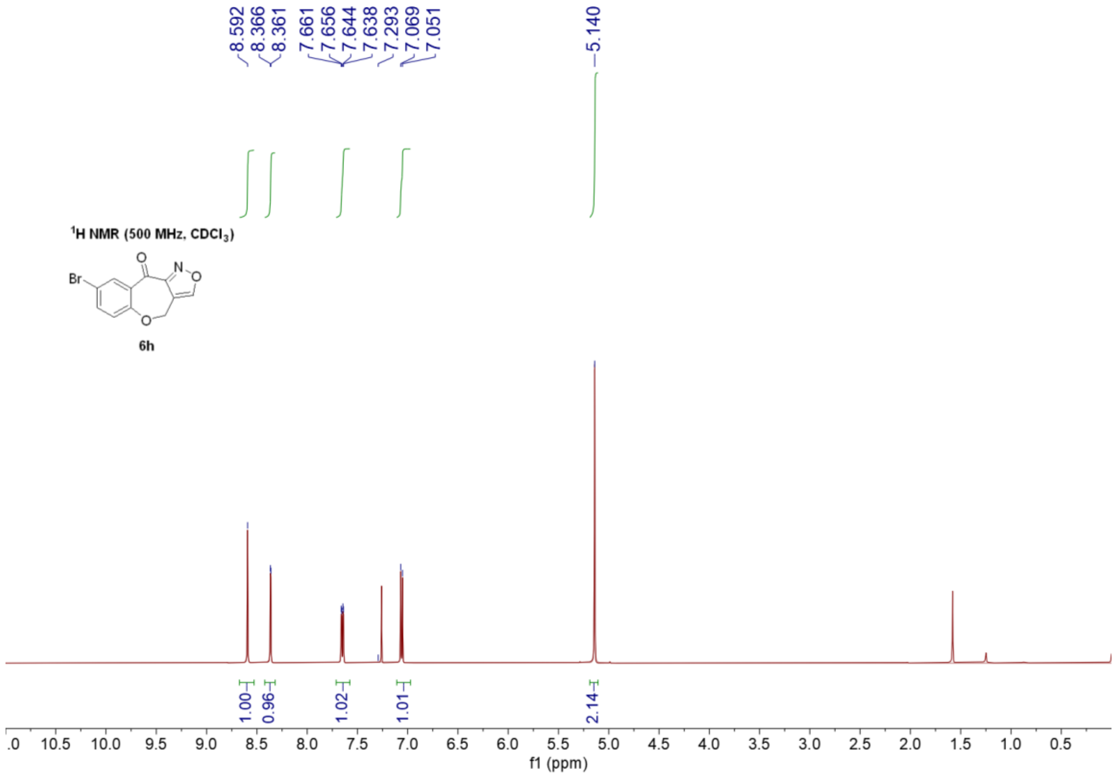
8.592
8.366
8.361
7.661
7.656
7.644
7.638
7.293
7.069
7.051
5.140
1H NMR (500 MHz, CDCl3)
6h
1.00
0.96
1.02
1.01
2.14
f1 (ppm)

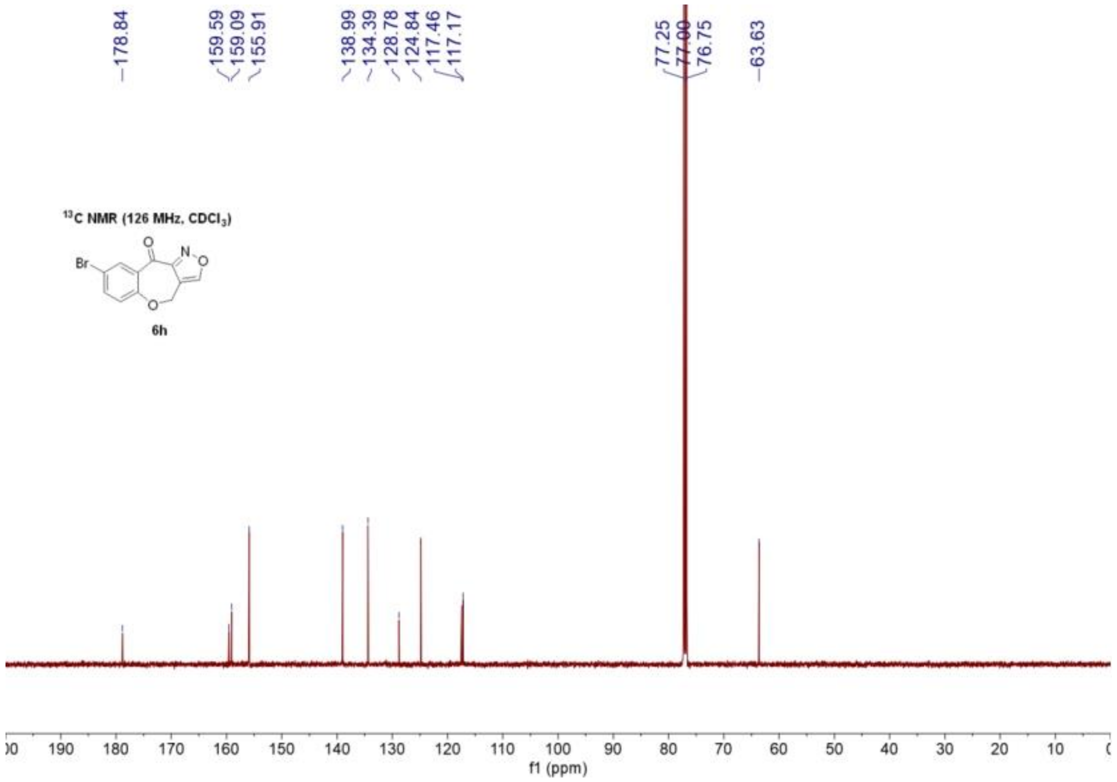
178.84
159.59
159.09
155.91
138.99
134.39
128.78
124.84
117.46
117.17
77.25
77.00
76.75
63.63
13C NMR (126 MHz, CDCl3)
6h
f1 (ppm)

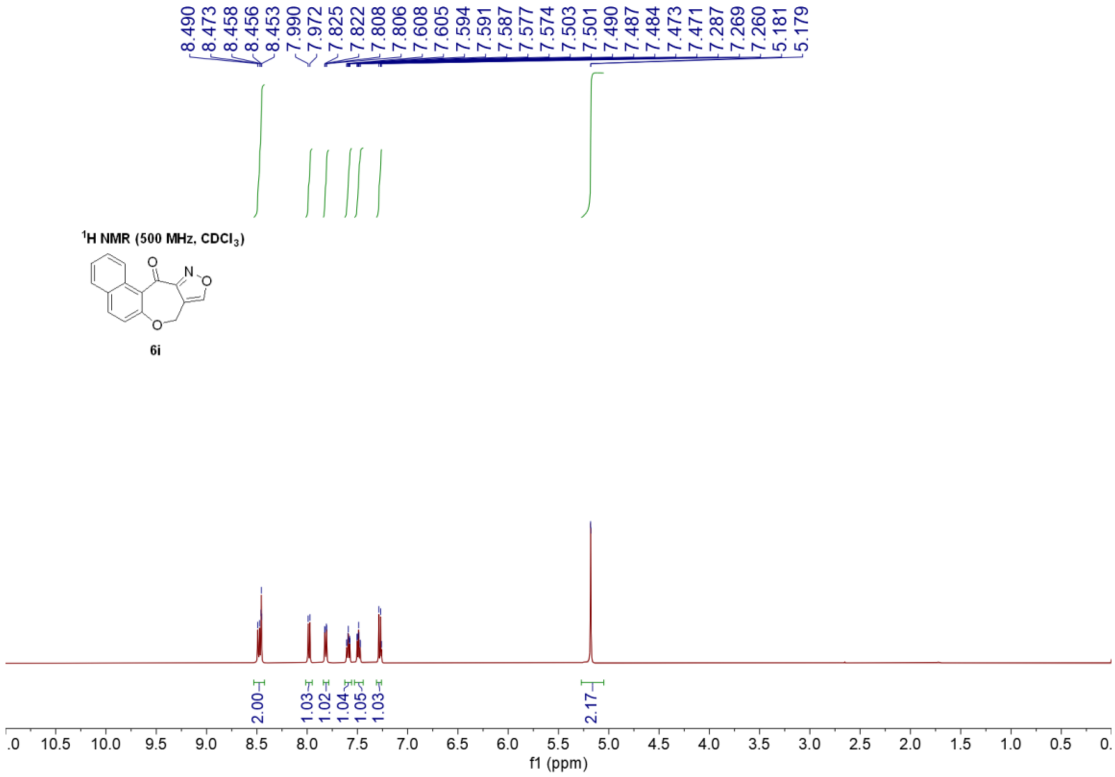
8.490
8.473
8.458
8.456
8.453
7.990
7.972
7.825
7.822
7.808
7.806
7.608
7.605
7.594
7.591
7.587
7.577
7.574
7.503
7.501
7.490
7.487
7.484
7.473
7.471
7.287
7.269
7.260
5.181
5.179
1H NMR (500 MHz, CDCl3)
6i
2.00
1.03
1.02
1.04
1.05
1.03
2.17
f1 (ppm)

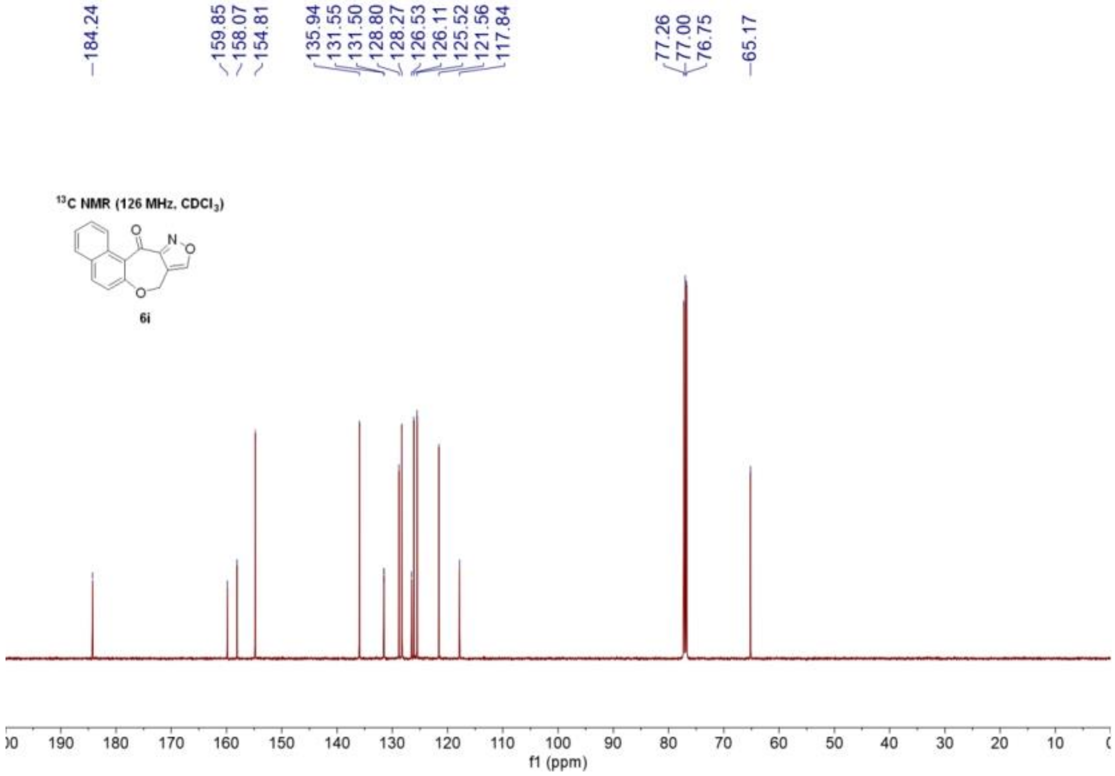
184.24
159.85
158.07
154.81
135.94
131.55
131.50
128.80
128.27
126.53
126.11
125.52
121.56
117.84
77.26
77.00
76.75
65.17
13C NMR (126 MHz, CDCl3)
6i
f1 (ppm)

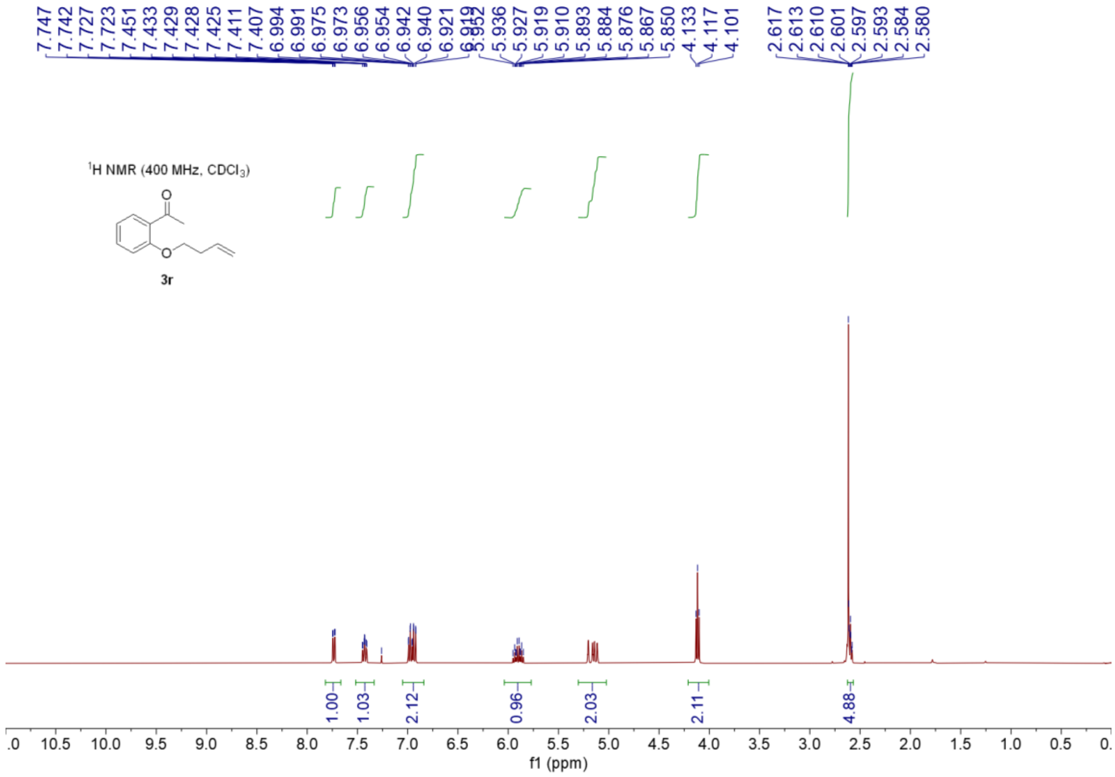
1H NMR (400 MHz, CDCl3)
3r
7.747 7.742 7.727 7.723 7.451 7.433 7.429 7.428 7.425 7.411 7.407 6.994 6.991 6.975 6.973 6.956 6.954 6.942 6.940 6.921 6.919 5.952 5.936 5.927 5.919 5.910 5.893 5.884 5.876 5.867 5.850 4.133 4.117 4.101 2.617 2.613 2.610 2.601 2.597 2.593 2.584 2.580
1.00 1.03 2.12 0.96 2.03 2.11 4.88
f1 (ppm)

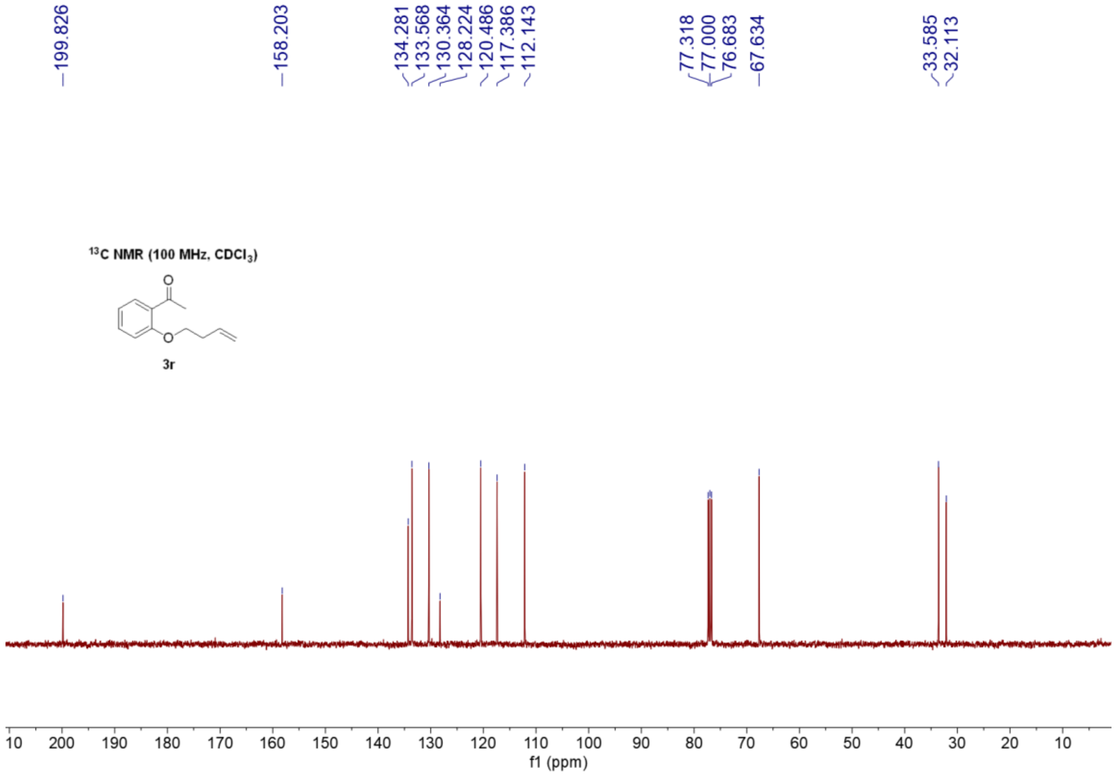
199.826 158.203 134.281 133.568 130.364 128.224 120.486 117.386 112.143 77.318 77.000 76.683 67.634 33.585 32.113
13C NMR (100 MHz, CDCl3)
3r
f1 (ppm)

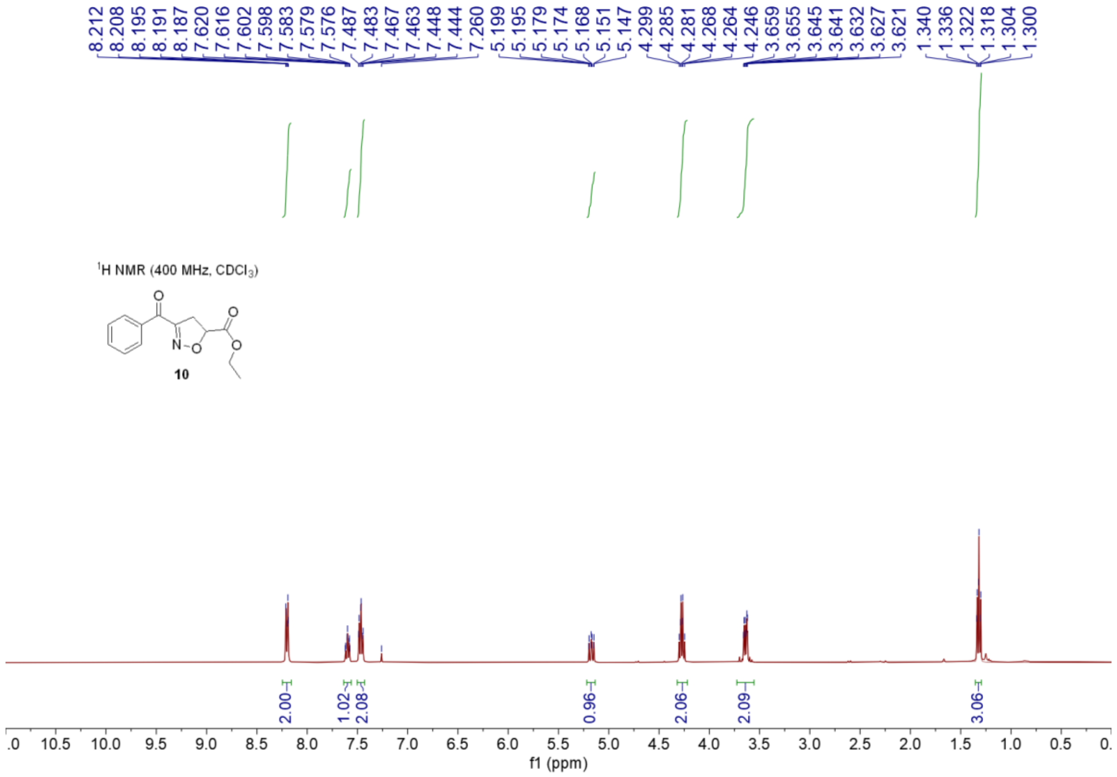

8.212 8.208 8.195 8.191 8.187 7.620 7.616 7.602 7.598 7.583 7.579 7.576 7.487 7.483 7.467 7.463 7.448 7.444 7.260 5.199 5.195 5.179 5.174 5.168 5.151 5.147 4.299 4.285 4.281 4.268 4.264 4.246 3.659 3.655 3.645 3.641 3.632 3.627 3.621 1.340 1.336 1.322 1.318 1.304 1.300
1H NMR (400 MHz, CDCl3)
O
N-O
O
O
10
2.00 1.02 2.08 0.96 2.06 2.09 3.06
10.5 10.0 9.5 9.0 8.5 8.0 7.5 7.0 6.5 6.0 5.5 5.0 4.5 4.0 3.5 3.0 2.5 2.0 1.5 1.0 0.5
f1 (ppm)

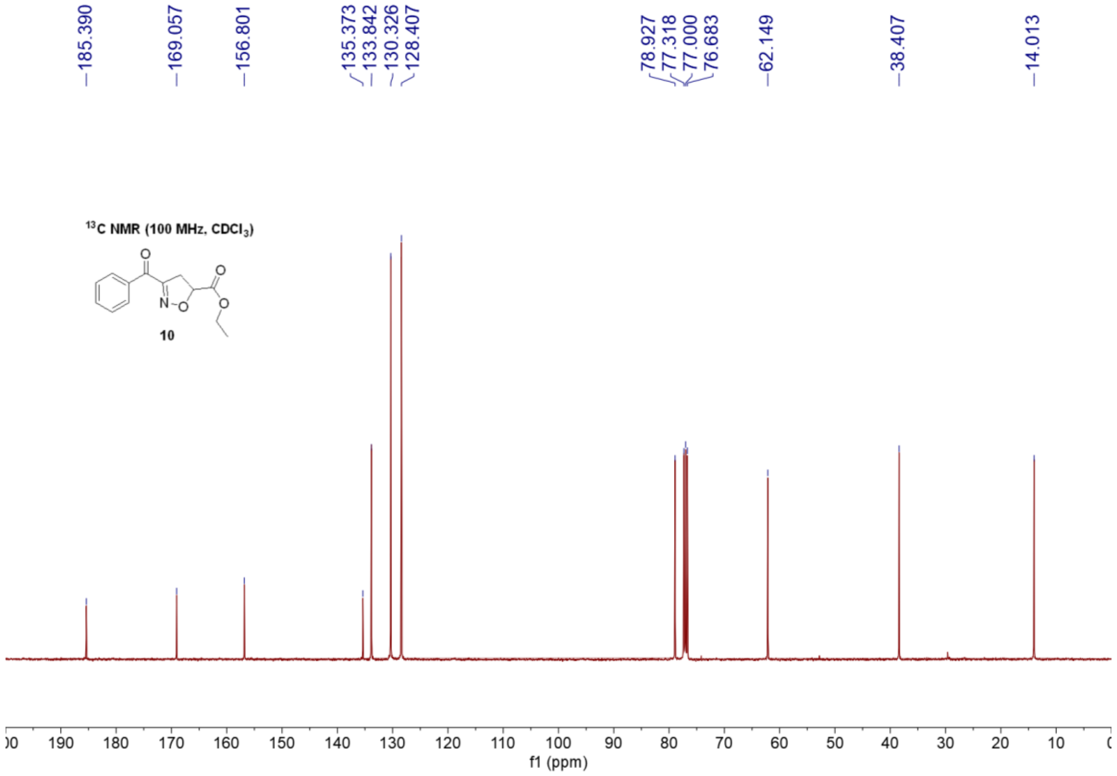

185.390 169.057 156.801 135.373 133.842 130.326 128.407 78.927 77.318 77.000 76.683 62.149 38.407 14.013
13C NMR (100 MHz, CDCl3)
O
N-O
O
O
10
190 180 170 160 150 140 130 120 110 100 90 80 70 60 50 40 30 20 10
f1 (ppm)

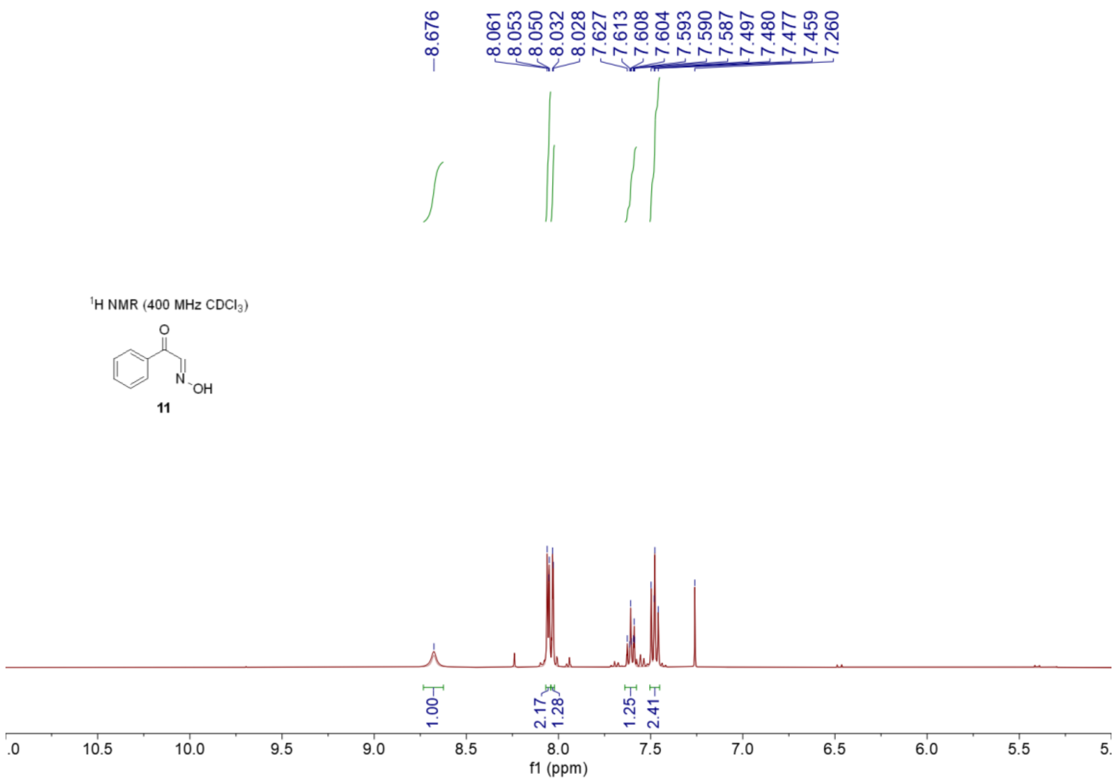

8.676
8.061
8.053
8.050
8.032
8.028
7.627
7.613
7.608
7.604
7.593
7.590
7.587
7.497
7.480
7.477
7.459
7.260
^{1}H NMR (400 MHz CDCl$_3$)
O
N
OH
11
1.00
2.17
1.28
1.25
2.41
f1 (ppm)

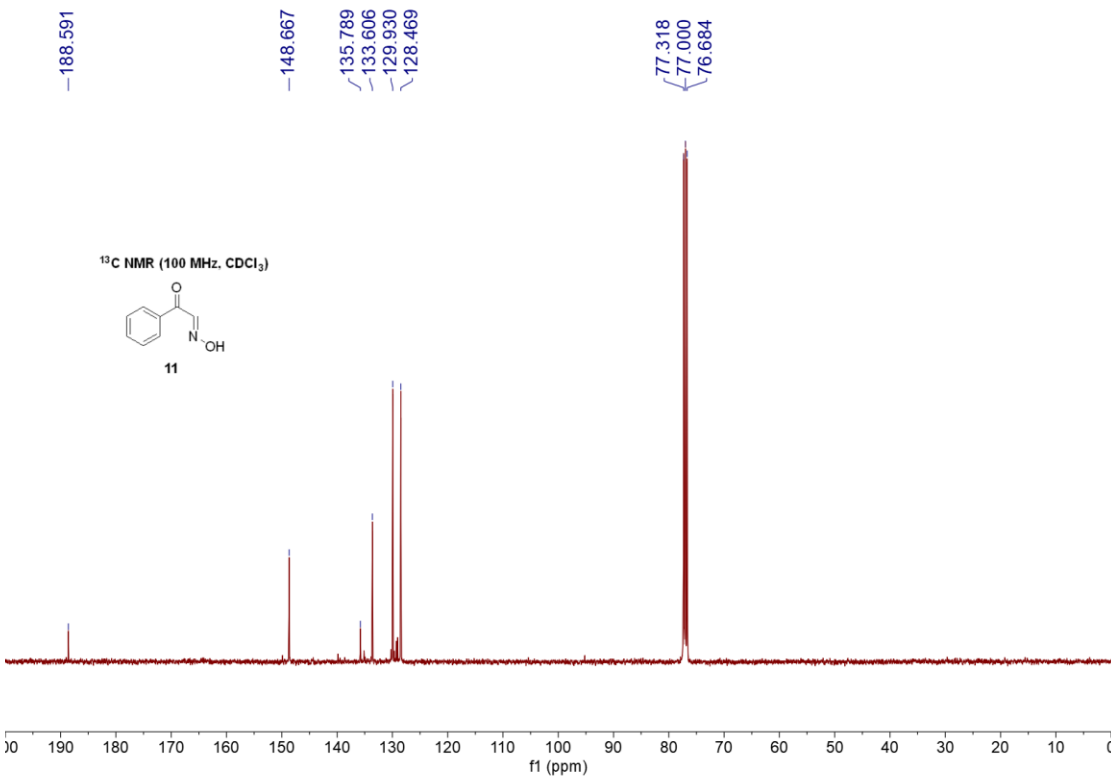

188.591
148.667
135.789
133.606
129.930
128.469
77.318
77.000
76.684
^{13}C NMR (100 MHz, CDCl$_3$)
O
N
OH
11
f1 (ppm)
